# Life expectancy can increase by up to 10 years following sustained shifts towards healthier diets in the United Kingdom

**DOI:** 10.1038/s43016-023-00868-w

**Published:** 2023-11-20

**Authors:** Lars T. Fadnes, Carlos Celis-Morales, Jan-Magnus Økland, Solange Parra-Soto, Katherine M. Livingstone, Frederick K. Ho, Jill P. Pell, Rajiv Balakrishna, Elaheh Javadi Arjmand, Kjell Arne Johansson, Øystein A. Haaland, John C. Mathers

**Affiliations:** 1 Department of Global Public Health and Primary Care, University of Bergen, Bergen, Norway; 2 Bergen Addiction Research, Department of Addiction Medicine, Haukeland University Hospital, Bergen, Norway; 3 School of Cardiovascular and Metabolic Health, University of Glasgow, Glasgow, UK; 4 Education, Physical Activity and Health Research Unit, University Católica del Maule, Talca, Chile; 5 Bergen Centre for Ethics and Priority Setting, University of Bergen, Bergen, Norway; 6 Department of Nutrition and Public Health, Universidad del Bío-Bío, Chillán, Chile; 7 Institute for Physical Activity and Nutrition (IPAN), School of Exercise and Nutrition Sciences, Deakin University, Geelong, Victoria Australia; 8 School of Health and Wellbeing, University of Glasgow, Glasgow, UK; 9 Human Nutrition and Exercise Research Centre, Centre for Healthier Lives, Population Health Sciences Institute, Newcastle University, Newcastle upon Tyne, UK

**Keywords:** Risk factors, Nutrition disorders

## Abstract

Adherence to healthy dietary patterns can prevent the development of non-communicable diseases and affect life expectancy. Here, using a prospective population-based cohort data from the UK Biobank, we show that sustained dietary change from unhealthy dietary patterns to the Eatwell Guide dietary recommendations is associated with 8.9 and 8.6 years gain in life expectancy for 40-year-old males and females, respectively. In the same population, sustained dietary change from unhealthy to longevity-associated dietary patterns is associated with 10.8 and 10.4 years gain in life expectancy in males and females, respectively. The largest gains are obtained from consuming more whole grains, nuts and fruits and less sugar-sweetened beverages and processed meats. Understanding the contribution of sustained dietary changes to life expectancy can provide guidance for the development of health policies.

## Abstract (web summary)

The UK Government aims to reduce premature deaths from non-communicable diseases by one-third by 2030 (SDG 3). Using life table methodology and data from the UK Biobank, this study indicates that sustained dietary changes—from median or unhealthy to a longevity-associated dietary pattern or to the Eatwell Guide recommendations—could lead to substantial life expectancy gains.

## Main

In the United Kingdom, unhealthy diets are estimated to cause more than 75,000 premature deaths each year, including almost 17,000 deaths in the age group 15–70 years^1^. Evidence on the mortality benefits of food choices is essential for the United Kingdom to achieve Sustainable Development Goal target 3.4, which is to reduce premature mortality from non-communicable diseases by one-third by 2030 (ref. ^2^) Internationally, the Global Burden of Diseases and Injuries consortium and the EAT–*Lancet* commission encourage healthy eating and quantify the population health that is associated with unhealthy eating^3–5^. Furthermore, Public Health England and the UK Government encourage the population to follow the diet pattern recommended in the Eatwell Guide to achieve a healthy and balanced diet^6^.

Life expectancy is a measure of expected years an individual has left to live and is a commonly used metric for population health. Higher adherence to the recommendations of the Eatwell Guide is associated with reduced mortality in the United Kingdom^7^, but it is not known how a sustained improvement in dietary patterns translates into gains in life expectancy at different stages of life. Estimating such gains in life expectancy would provide policymakers with a measure of the health gains that are possible in a population and provide guidance on which policies would be the most effective. Furthermore, health personnel would also benefit in identifying key risks related to unhealthy dietary patterns with the highest potential for gain when guiding people to prioritize relevant behaviour changes. In addition, most people do not adhere to healthy eating guidelines^8^, with research showing that less than 0.1% of the UK population adheres to all recommendations of the Eatwell Guide^7^. Therefore, it is important to estimate the gains in life expectancy that are expected from different types of dietary change and various degrees of adherence to the recommendations.

Recently, we developed a model for estimating age- and sex-specific gains or losses in life expectancy following sustained change (that is, dietary changes for remaining lifespan) in the consumption of major food groups with measured intakes^9^. In this paper, we use this model to estimate life expectancy gains from a sustained change from median or unhealthy dietary patterns in the United Kingdom to the longevity-associated dietary pattern, or to the recommendations of the Eatwell Guide.

**Fig. 1:**
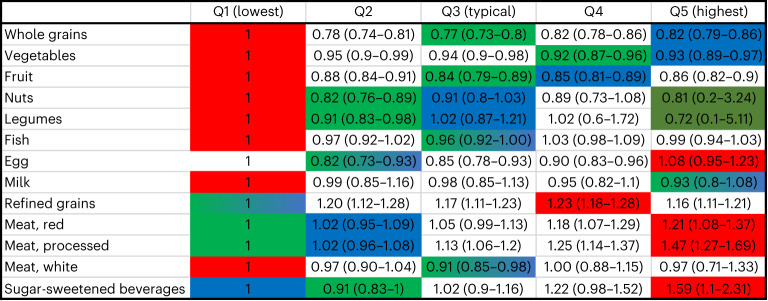
Hazard ratio for all-cause mortality per food group for each quintile (Q1–Q5) among UK Biobank participants. Data are presented as hazard ratios and their 95% confidence intervals. The reference groups were the lowest quintile of intake for each food group. The analyses were adjusted for age, sex, area-based socio-demographic deprivation, smoking, alcohol consumption and physical activity level. The unhealthy categories are shown in red, the longevity associated are shown in green and dark green, and the Eatwell recommendations are shown in blue. The dark green category had large uncertainties; thus, the robust version of the healthiest dietary patterns is in green (not dark green).

The median dietary patterns in the United Kingdom were categorized as the intake level with the mid-quintile intake of each of the food groups from the UK Biobank data, from 467,354 participants. The longevity-associated dietary patterns were the quintiles for each food group with the lowest mortality risk estimates or second lowest if the confidence intervals were very wide (Fig. 1). For the unhealthy eating pattern, we used the quintiles for each food group with the highest mortality association, while for the Eatwell Guide, we chose the quintiles of daily intakes fitting best the Eatwell Guide. A detailed description of the methodology is described in Supplementary Information.

## Results

Our results showed that the longevity-associated dietary pattern had moderate intakes of whole grains, fruit, fish and white meat; a high intake of milk and dairy, vegetables, nuts and legumes; a relatively low intake of eggs, red meat and sugar-sweetened beverages; and a low intake of refined grains and processed meat (Fig. 1). Analyses adjusting also for body mass index and energy (Supplementary Information) showed slight reductions in inverse associations with mortality for whole grains, vegetables and fruits, reductions in positive associations with mortality for red meat, and stronger inverse associations for both nuts and white meat. For several of the food groups associated with reduced mortality, the lowest intake quintiles were substantially different from the other quintiles. The unhealthy dietary pattern (that is, the quintile with the highest mortality associations) contained no or limited amounts of whole grains, vegetables, fruits, nuts, legumes, fish, milk and dairy, and white meat and substantial intakes of processed meat, eggs, refined grains and sugar-sweetened beverages. The strongest positive associations with mortality were for sugar-sweetened beverages and processed meat, while the strongest inverse associations with mortality were for whole grains and nuts.

**Fig. 2:**
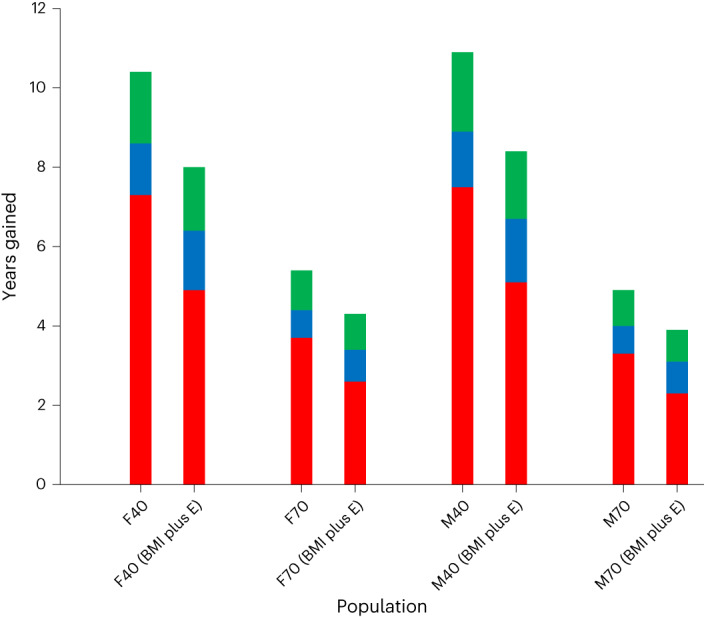
Expected life years gained from dietary changes. Expected life years gained after changing from unhealthy median dietary patterns (red), changing from median dietary patterns to the Eatwell Guide (blue) and changing from the Eatwell Guide to longevity-associated dietary patterns (green) for 40-year-old female and male adults (F40 and M40, respectively) and 70-year-old female and male adults (F70 and M70, respectively) from the United Kingdom. Both core-adjusted models (adjusted for age, sex, socio-demographic area, smoking, alcohol consumption and activity level) and mediation models (adjusted for energy and body mass index (BMI plus E)) are presented.

We present life expectancy estimates with uncertainty intervals (UI, indicating the lowest and highest population means that are likely) associated with various dietary patterns in Fig. 2, Table 1 and Supplementary Information. The life expectancy (that is, the estimated remaining years to live) of a 40-year-old with a median dietary pattern was 44.7 years for females and 41.5 years for males. Similarly, the life expectancy of a 70-year-old with a median dietary pattern was 17.6 years for females and 15.5 years for males. Estimated gains from simulated sustained dietary change from a median UK diet pattern to the longevity-associated diet pattern were 3.1 years (UI 1.3–4.9 years) for 40-year-old females and 3.4 years (UI 1.4–5.3 years) for 40-year-old males. Correspondingly, for sustained change to the Eatwell dietary pattern, estimated gains were 1.3 years (UI 0.1–2.4 years) for females and 1.4 years (UI 0.1–2.6 years) for males. Estimated gains from sustained dietary changes from an unhealthy UK diet pattern to the longevity-associated diet pattern were 10.4 years (UI 8.2–11.3 years) for 40-year-old females and 10.8 years (UI 8.8–12.0 years) for 40-year-old males. Correspondingly, estimated gains from sustained dietary changes from an unhealthy UK diet pattern to the longevity-associated diet pattern were 5.4 years (UI 4.4–6.0 years) for 70-year-old females and 5.0 years (UI 4.2–5.6 years) for 70-year-old males.

**Table 1:** Life expectancy associated with various dietary patterns among UK females and males aged 40 and 70 years, and life expectancy gains with uncertainty intervals (UI) changes in dietary patterns

Group	MUK (years)	UUK (years)	EUK (years)	LUK (years)	MUK ➜ LUK (UI years)	UUK ➜ LUK (UI years)	MUK ➜ EUK (UI years)	UUK ➜ EUK (UI years)
UK females, 40 years old	44.7	37.4	46	47.8	3.1 (1.3, 4.9)	10.4 (8.2, 11.3)	1.3 (0.1, 2.4)	8.6 (6.8, 10.2)
UK females, 70 years old	17.6	13.9	18.4	19.3	1.7 (0.7, 2.6)	5.4 (4.4, 6.0)	0.7 (0.0, 1.3)	4.4 (3.6, 5.4)
UK males, 40 years old	41.5	34	42.9	44.8	3.4 (1.4, 5.3)	10.8 (8.8, 12.0)	1.4 (0.1, 2.6)	8.9 (7.2, 10.8)
UK males, 70 years old	15.5	12.2	16.2	17.1	1.6 (0.7, 2.5)	5.0 (4.2, 5.6)	0.7 (0.0, 1.2)	4.0 (3.4, 5.1)
UK females, 40 years old^a^	44.7	39.7	46.1	47.7	3.1 (1.3, 5.1)	8.0 (5.3, 9.7)	1.5 (−0.2, 2.9)	6.4 (4.1, 8.8)
UK females, 70 years old^a^	17.6	15.1	18.4	19.3	1.7 (0.7, 2.7)	4.3 (2.8, 5.2)	0.8 (−0.1, 1.5)	3.4 (2.2, 4.7)
UK males, 40 years old^a^	41.5	36.3	43	44.8	3.3 (1.4, 5.4)	8.4 (5.7, 10.4)	1.6 (−0.2, 3.1)	6.7 (4.3, 9.3)
UK males, 70 years old^a^	15.4	13.1	16.2	17.1	1.6 (0.7, 2.6)	3.9 (2.7, 4.9)	0.8 (−0.1, 1.5)	3.1 (2.1, 4.3)

^a^Extensively adjusted models also adjusted for energy and body mass index.

Dietary changes presented: MUK ➔ LUK: from median UK diet (MUK) to longevity-associated diet patterns (LUK); UUK ➔ LUK: from unhealthy UK diet patterns (UUK) to LUK; MUK ➔ EUK: from MUK to the Eatwell Guide dietary pattern (EUK); UUK ➔ EUK: from UUK to EUK.

Estimated gains from simulated sustained dietary changes from an unhealthy UK diet pattern to full adherence to the Eatwell Guide were 8.6 years (UI 6.8–10.2 years) for 40-year-old females and 8.9 years (UI 7.2–10.8 years) for 40-year-old males. Corresponding gains for 70-year-old females and males were 4.4 years (UI 3.6–5.4 years) and 4.0 years (UI 3.4–5.1 years), respectively.

In sensitivity analyses, sex-stratified analyses of food groups and associations with mortality generally showed similar associations across the sexes except for white meat, which seemed to be more beneficial among females. A range of other sensitivity analyses are presented in Supplementary Information. To reduce potential reverse causation, we performed a landmark analysis that excluded events that occurred within the previous 2 years.

## Discussion

In this paper, we present a method for estimating changes in life expectancy following changes in food choices, considering correlation between mortality and food group intakes, and effect delay. Such estimates may be useful particularly for policy purposes and for underpinning both guidance and interventions for improving public health. Our results indicate that UK adults aged 40 years with median dietary patterns can expect to gain approximately 3 years in life expectancy from sustained changes to the longevity-associated dietary patterns. Importantly, the estimated gain in life expectancy is approximately a decade for those shifting from the unhealthiest to the longevity-associated dietary patterns. Overall, the bigger the changes made towards healthier dietary patterns, the larger the expected gains in life expectancy are.

Consuming less sugar-sweetened beverages and processed meats and eating more whole grains and nuts were estimated to result in the biggest improvements in life expectancy. Sensitivity analysis also adjusting for body mass index and energy consumption indicated that body mass index and energy consumption might partially mediate and/or confound a possible beneficial effect between life expectancy and whole grains, vegetables and fruits, and inversely for red meat and eggs. For white meat, associations were stronger when adjusting for energy intake and body mass index, while the situation was mixed for legumes. These estimates correspond well with meta-analyses on associations between intakes of food groups and mortality^10–15^. Our estimates from the UK Biobank are also strengthened by meta-analyses of randomized trials on the consumption of various food groups and scoring of biomarkers for disease^16^, mirroring our estimates with nuts, legumes and whole grains performing most beneficially and sugar-sweetened beverages and red meat performing worst. We have also presented estimated health gains associated with adhering to the Eatwell Guide recommendations, showing that the Eatwell Guide does well on the longevity perspective. The life expectancy gains from changes from unhealthy eating to the Eatwell Guide achieve 82–83% of the potential compared with those from sustained change to longevity-associated patterns, which strengthens the evidence base for the promotion of the dietary targets in the Eatwell Guide in public health guidance and interventions. The Eatwell Guide might also be a more realistic target for dietary changes than the statistically set longevity-associated dietary patterns.

Unsurprisingly, predicted gains in life expectancy are lower when the dietary change is initiated at older ages, but these remain substantial. For example, we estimated that people at the age of 70 years could expect to benefit from about half of the life expectancy gain predicted for adults at the age of 40 years, equivalent to a gain in 1.5 years when optimizing median dietary patterns and 4–5 years for those shifting from the unhealthiest dietary patterns. The UK population currently has a life expectancy at birth of 83.6 years for females and 79.9 years for males, and a 3 year gain in life expectancy associated with changes from median to longevity-optimized dietary patterns from the age of 40 years (ref. ^17^). Life expectancies have steadily increased over time^18^, and the observed increase is parallel to the changes in life expectancy observed in the United Kingdom over the past 15 years^17^. A large shift towards healthy dietary patterns could contribute substantially to meeting Sustainable Development Goal target 3.4 that aims to cut premature mortality by one-third.

The governmental food strategy in the United Kingdom to address chronic diseases emphasizes a shared responsibility, with the industry having a responsibility to promote and supply healthier foods, the government having a role in making targeted regulatory interventions to support change, and individual consumers being empowered with better information about healthier choices and thus demanding and seeking healthier foods^19^. With respect to potential policy actions, a recent paper presented approaches to address the substantial inequalities in health in the United Kingdom^20^. The paper argues for five principles, including healthy-by-default and easy-to-use initiatives, long-term and multisector action, locally designed focus, targeting disadvantaged communities and matching of resources to need. The paper further identifies various actions that could contribute to improvements, such as health-oriented food taxes and subsidies aiming to reduce the cost of healthy foods but not that of unhealthy foods. Other actions were related to improving food environments in school, public and working places by removing vending machines and banning the sale of sugar-sweetened beverages and snacks high in fat, sugar or salt. Such policy measures, informed by the up-to-date estimates on potential gains in life expectancy that we provide in this paper, could guide the deployment of resources to improve healthy eating patterns across the population.

Limitations of our study include correlation between the associations between food groups and mortality. We addressed this through model adjustments presenting a conservative model as the main analyses. As for most cohort studies, confounders can have an impact. However, we have adjusted our core models for several potential confounders such as age, sex, area-based socio-demographic deprivation, smoking, alcohol consumption and physical activity level. We have also added sensitivity analyses investigating for mediation and possible confounding from body mass index and energy intake. Our models are based on population data with the uncertainty measure being on the population-level mean. Nevertheless, even though the individual uncertainty is likely to be larger than uncertainty at the group level, one could expect the mean life expectancy differences of groups of individuals with similar characteristics but variations in group size to be comparable (with uncertainty being higher in smaller groups). Thus, our estimates could be useful also in clinical settings. We model sustained and prolonged dietary changes. However, maintaining lifestyle changes over time with dietary improvements can be challenging, and for many, dietary patterns fluctuate over time. We have not modelled potential changes in life expectancy of fluctuating changes. Furthermore, unhealthy and longevity-associated dietary patterns could be seen as dietary ‘constructs’ (as associations between dietary patterns and mortality are used to set these). This is not necessarily overlapping with typically presented dietary patterns such as the Mediterranean or the Nordic diets. Furthermore, the UK Biobank does not measure consumption of rice, which is particularly important for many migrant groups. Overall, the UK Biobank data under-represent non-white populations compared to the UK population. Even though the UK Biobank data contain about nearly half a million participants in our analyses, the size of the data is not sufficient to achieve precise estimates across all quintiles and there seems to be some random fluctuations between some of the quintiles. Thus, the exact quintile threshold should be interpreted with caution, and more emphasis should be placed on the general trends. There were limited and selective data in the dietary recall that could result in biases.

In conclusion, for middle-aged adults in the United Kingdom, sustained dietary improvement is predicted to increase life expectancy by about 3 years for both females and males. Importantly, for those with the least healthy dietary patterns, change to the longevity-associated dietary pattern is predicted to translate into approximately 10 years gain in life expectancy. Changing from an unhealthy dietary pattern to eating in line with the Eatwell Guide was associated with an 8 year life expectancy gain. Gains in life expectancy are lower the longer the delay in the initiation of dietary improvements, but even for those initiating dietary change at age 70 years, the gain in life expectancy is about half of that achieved by 40-year-old adults. The biggest gains in life expectancy are associated with increased intake of whole grains and nuts, and with reduced intake of sugar-sweetened beverages and processed meats. Our findings suggest that these food groups should be specific targets for clinicians in the guidance of patients and policymakers in developing public health policy.

## Methods

The study complies with all relevant ethical regulations (with approval obtained by UK Biobank from the National Health Service National Research Ethics Service (reference 11/NW/0382). All data included are from participants who provided written informed consent for use of their data. Detailed methods beyond the shortened description in this Brief Communication are provided in Supplementary Information.

### Reporting summary

Further information on research design is available in the Nature Portfolio Reporting Summary linked to this article.

### Supplementary information

Supplementary Information Supplementary text, code, Figs. 1–11, Tables 1–9 and TRIPOD checklist.

Reporting Summary

## Data Availability

Requests for the dataset can be sent through UK Biobank.

## Code availability

The R code is available in Supplementary Information.
