# Supplementary material for: Life expectancy can increase by up to 10 years following sustained shifts towards healthier diets in the United Kingdom
Source: Nat Food. 2023 Nov 20;4(11):961–5. doi: 10.1038/s43016-023-00868-w (PMC10661734; doi:10.1038/s43016-023-00868-w)
Supplement: Supplementary file 1 — Supplementary text, code, Figs. 1–11, Tables 1–9 and TRIPOD checklist. [file 43016_2023_868_MOESM1_ESM.pdf]

nature food

Brief Communication https://doi.org/10.1038/s43016-023-00868-w

Life expectancy can increase by up to 10 years following sustained shifts towards healthier diets in the United Kingdom

In the format provided by the authors and unedited

Supplemental materials for *Estimating life expectancy gains from dietary changes in the United Kingdom*

Text S1: Policy summary.

Text S2: Methodology and additional details related to UK Biobank and dietary assessment.

Text S3: Data analysis code in R.

Table S1. Details on diet component from the UK Biobank study.

Table S2. All-cause mortality hazard ratios for various food groups with uncertainty limits for the UK based on estimates from UK Biobank.

Table S3. Table presenting correlation between food intake of the different food groups for the UK Biobank cohort.

Table S4. Intake categories per food group for each quintile (Q1-Q5) among people in the UK based on data from UK Biobank.

Table S5. Sensitivity analysis assessing potential mediation and/or confounding by energy and body mass index with associations from UK Biobank between various food groups with all-cause mortality hazard ratios. A. First presented hazard ratios for models with core adjustment with age, sex, socio-demographic area, smoking, alcohol consumption, and activity level. B. Then adding core adjustment when adding adjustments body mass index, and also core adjustment when adding adjustments for energy and body mass index (b).

Table S6. Sensitivity analysis of all-cause mortality hazard ratios for various food groups with uncertainty limits for the UK based on estimates from UK Biobank using time-to-full-effect of 5, 20 and 50 years, and model adjustments of 0.25, 0.5 and 1.0.

Table S7. Number of food frequency questionnaires.

Table S8. Background characteristics of participants.

Fig S1. Expected life years gained per food group presented in forest plots with uncertain intervals for 40-year-old female adults from the UK who changes from a: median to Eatwell Guide patterns (upper left), b: median to healthiest (lower left), c: unhealthy to Eatwell Guide patterns (upper right), d: unhealthy to healthiest (lower right).

Fig S2. Expected life years gained per food group presented in forest plots with uncertain intervals for 40-year-old male adults from the UK who changes from a: median to Eatwell Guide patterns (upper left), b: median to healthiest (lower left), c: unhealthy to Eatwell Guide patterns (upper right), d: unhealthy to healthiest (lower right).

Fig S3. Expected life years gained per food group presented in forest plots with uncertain intervals for 70-year-old female adults from the UK who changes from a: median to Eatwell Guide patterns (upper left), b: median to healthiest (lower left), c: unhealthy to Eatwell Guide patterns (upper right), d: unhealthy to healthiest (lower right).

Fig S4. Expected life years gained per food group presented in forest plots with uncertain intervals for 70-year-old male adults from the UK who changes from a: median to Eatwell Guide patterns (upper left), b: median to healthiest (lower left), c: unhealthy to Eatwell Guide patterns (upper right), d: unhealthy to healthiest (lower right).

Fig S5. Sensitivity analysis with expected life years gained per food group presented in forest plots with uncertain intervals for 40-year-old female adults from the UK who changes from median to healthiest diet patterns with a: time-to-full effect of 5 years (upper left), b: time-to-full effect of 50 years (lower left), c: conservative model with m=0.25 (upper right), d: model not assuming overlap with m=1.0.

Fig S6. Sensitivity analysis with expected life years gained per food group presented in forest plots with uncertain intervals for 70-year-old male adults from the UK who changes from median to healthiest diet patterns with a: time-to-full effect of 5 years (upper left), b: time-to-full effect of 50 years (lower left), c: conservative model with m=0.25 (upper right), d: model not assuming overlap with m=1.0.

Fig S7. Expected life years gained per food group while adjusting for body mass index presented in forest plots with uncertain intervals for 40-year-old female adults from the UK who changes from a: median to Eatwell Guide patterns (upper left), b: median to longevity-associated (lower left), c: unhealthy to Eatwell Guide patterns (upper right), d: unhealthy to longevity-associated (lower right).

Fig S8. Expected life years gained per food group while adjusting for body mass index presented in forest plots with uncertain intervals for 70-year-old male adults from the UK who changes from a: median to Eatwell Guide patterns (upper left), b: median to longevity-associated (lower left), c: unhealthy to Eatwell Guide patterns (upper right), d: unhealthy to longevity-associated (lower right).

Fig S9. Expected life years gained per food group while adjusting for body mass index and energy presented in forest plots with uncertain intervals for 40-year-old female adults from the UK who changes from a: median to Eatwell Guide patterns (upper left), b: median to longevity-associated (lower left), c: unhealthy to Eatwell Guide patterns (upper right), d: unhealthy to longevity-associated (lower right).

Fig S10. Expected life years gained per food group while adjusting for body mass index and energy presented in forest plots with uncertain intervals for 70-year-old male adults from the UK who changes from a: median to Eatwell Guide patterns (upper left), b: median to longevity-associated (lower left), c: unhealthy to Eatwell Guide patterns (upper right), d: unhealthy to longevity-associated (lower right).

Fig S11: Weight of mortality associated with “initial diet”.

Checklist S1: Tripod Checklist.

Text S1: Policy summary.

Background

Healthy eating with plenty of fruits, vegetables, nuts, and whole grains and limited red and processed meats, is associated with reduced mortality and longevity. However, there is limited research which quantifies the potential gains from changes in specific food groups.

Main findings and limitations

In this study, we present estimated longevity gain from sustained change from median to the longevity-associated dietary patterns in the United Kingdom, which is estimated to be 3 years for 40-year-old females and males. Estimated longevity gain from sustained change from unhealthy to the longevity-associated dietary pattern was about 10 years in 40-year-old females and males. The unhealthy dietary patterns are characterized by limited amounts of whole grains, nuts, legumes, dairy, vegetables and fruits, fish, and white meat, but a high intake of red and processed meats, eggs, as well as refined grains and sugar-sweetened beverages. Sustained change from unhealthy to the Eatwell Guide patterns is associated with gains of around 8 years for 40-year-old females and males. The largest gains would be made by eating more whole grains, nuts, and fruits, and less sugar-sweetened beverages, red and processed meat, and eggs. Limitations include correlation between food groups. This was addressed through model adjustments presenting a conservative model as the main analyses while also adjusting for factors such as smoking, age, sex, and social deprivation. Our estimates should be seen as population estimates and not individual forecasting.

Policy implications

Our findings suggest that these food groups should be specific targets for clinicians in guidance of patients, and policy makers in developing public health policy. The models are presented in a web application at http://uk.food4healthylife.org/

Text S2: Additional details on methodology related to UK Biobank, dietary assessment, and model adjustments.

METHODS

UK Biobank is a prospective cohort study. A total of over 500,000 participants aged 37-73 years at baseline were enrolled, from the general population. In brief, between 2006 and 2010, participants attended one of 22 assessment centres across Scotland, England, and Wales. All participants completed a touch-screen questionnaire, had physical measurements taken, and provided blood, urine, and saliva samples at baseline. A subset of 467,354 participants in UK Biobank for whom diet was assessed (Text S1, Table S1).

In the UK Biobank cohort, dates and causes of death were available from death certificates held by the National Health Service (NHS) Information Centre (England and Wales) and the NHS Central Register Scotland (Scotland). Mortality data were available until June 2020. Therefore, follow-up was censored on these dates.

The Eatwell Guide

The Eatwell Guide recommends consumption of at least five portions of a variety of fruit and vegetables daily, base meals on potatoes, bread, rice, pasta, or other starchy carbohydrates, and choose higher fibre wholegrain varieties, including some dairy or alternatives to dairy such as soya drinks and yoghurts, and choosing lower-fat and lower-sugar products where possible. The Guide further recommends eating some beans, pulses, fish, eggs, meat, and other protein, choosing lean cuts of meat and mince, and eating less red and processed meat such as bacon, ham, and sausages, and aiming for at least two portions of fish every week of which one should be oily (such as salmon or mackerel). It also recommends choosing unsaturated oils and spreads, and eating these in small amounts. Foods high in fat, salt and sugar should be eaten less often, and in small amounts.

Dietary assessment

Information on median dietary patterns in the UK Biobank cohort was assessed at least once using the web-based 24-hour dietary questionnaire Oxford WebQ,[1-4] and categorized into the food groups fruits, vegetables, whole grains, refined grains, nuts, legumes, fish, eggs, milk/dairy, red meat, processed meat, white meat, and sugar-sweetened beverages. We split the intake of all food groups (in grams) into quintiles, and the median dietary patterns in the UK was based on the mid-quintile (Table 1). Quintiles were constructed by ordering the intake levels in each food groups and then splitting into five groups being as balanced as possible. Table S4 presents range of intake per food group category with minimum and maximum in each quintile. The longevity-associated dietary patterns were the quintiles for each food group with the lowest mortality risk estimates or second lowest if the confidence intervals were very wide (Table 1). The outcomes were also presented based on meeting the dietary pattern recommended by the UK's Eatwell Guide.[5] The following thirteen food items were included: whole grains, vegetables, fruits, nuts, legumes, fish, milk/dairy, red, white and processed meat, eggs, refined grains, and sugar-sweetened beverages. The reference groups for the Eatwell recommendations were chosen as the quintile with intake levels closest to the Eatwell recommended amounts for each food item.

Covariates

Age at baseline was determined from dates of birth and baseline assessment. Sex was self-reported at baseline. Deprivation (area-based socioeconomic status) was derived from the postcode of residence, using the Townsend score.[6] Ethnicity/race was self-reported and

categorised as white, black, South Asians, Chinese, mixed, and others. Self-reported smoking status was categorised as never, former, or current smoker. Chronic conditions were self-reported in a nurse-led interview at baseline. Finally, total physical activity was self-reported using the International Physical Activity Questionnaire short form.[7] Body mass index was calculated as weight (kg) divided by height (m) squared. Additional information on the measurements is available on the UK Biobank website (http://www.ukbiobank.ac.uk).

Statistical analysis

Following the approaches of Johansson *et al.* 2020 and Fadnes *et al.* 2022,[8,9] our model for estimating gains or losses in life expectancy after sustained dietary change subtracts the life expectancy for individuals on the baseline diet from the life expectancy of individuals after dietary changes. For example, denoting by $LE_{MUK;age,sex}$ and $LE_{D;age,sex}$ the age- and sex-specific life expectancies for individuals on a median UK diet (MUK) and a new diet (D) we get that the gain (or loss) in life expectancy by a sustained change form MUK to D is $\Delta_{D,MUK;age,sex}=LE_{D;age,sex} - LE_{MUK;age,sex}$. We defined MUK as the median intake in all food groups based on the UK Biobank data.

$LE_{MUK;age,sex}$ is calculated using average sex- and age-specific mortality rates for the UK obtained from the Global Burden of Diseases and Injuries study (GBD).[10] When estimating $LE_{D;age,sex}$, mortality rates were increased or reduced by a rate corresponding to the components of D. These rates were calculated running the adjusted Cox proportional hazards model on the UK Biobank data (see above and Table S2). In other words, starting from the age of sustained dietary change, the age-specific background mortality was multiplied by the hazard rates corresponding to the intake of each of the food groups so that the total impact of all the food groups was:

$$HR_{Total}=\prod_G(HR_G) ,$$

where HR_G was the HR for food group G. For example, if the hazard ratio was 0.9 (reducing mortality) for one food group and 1.2 (increasing mortality) for another, the combined impact on mortality of the two groups was assumed to be 0.9×1.2=1.08 (an increase of 8%). To account for the expected time delay between dietary change and the development of diseases such as cardiovascular disease and cancer,[11-13] while weighting in the morbidity burden,[14-18] we assumed that time to full effect was 20 years with an inverse S-shaped decrease in mortality (Figure S11, with sensitivity analyses for 5 years to 50 years).

Our approach to calculate uncertainty intervals (UI) for the gains or losses in life expectancy from dietary changes was based on Mathers *et al.* (2006).[19] For food group G, we obtained the regression coefficient $\beta_G=\log(HR_G)$ with standard error $SE_G=SE(\log(HR_G))$ from the above-mentioned Cox regression. We next sampled a number at random from a normal distribution with mean β_G and standard deviation SE_G and transformed this number back into a hazard ratio, $HR_{G,1}$. This procedure was repeated for all the food groups, and their simulated hazard ratios were multiplied to obtain the combined impact on mortality, $HR_{Total,1}$. Now $LE_{D;age,sex,1}$ and $\Delta_{D,1}$ could be calculated. Repeating this procedure 200 times, uncertainty limits were selected between the 6th and the 195th when ranked from lowest to highest.

Intakes of some food groups correlate with the intakes of other food groups (Table S3), and if the Cox regression analyses is not able to account fully for this correlation, the resulting HR_{Total} may be biased. Therefore, our model included a parameter m, ranging from 0 to 1.0, to calculate an adjusted hazard ratio, HR_a. When m=1 we assume residual confounding so that

$HR_a = HR_{Total}$, and when m=0, $HR_a=1$ (i.e., no change in mortality from the new diet). In our main analyses, we used m=0.5, which meant that HR_a was moved half-way between the HR_{Total} and 1. We also conducted analyses for m=0.75 and m=1.0 (less conservative) and m=0.25 (more conservative).

The Cox regression was analysed using the survival package in R and the Cox proportional hazard (coxph) function. We used the R package Shiny to create a web application (http://uk.food4healthylife.org/) that enables the estimation of the association between dietary changes and life expectancy. [20,21] In the left food panel indicating dietary pattern before change, the defaults are set to the "median UK dietary pattern" (the mid quintiles). The right food panel represents the dietary pattern after change. After clicking the "*New diet: Healthiest*" (longevity-associated) or "*New diet: Eatwell*" button, the right panel of sliders are adjusted to the longevity-associated and Eatwell Guide dietary patterns. In this paper, we present estimated gain in life expectancy when changing from a median or unhealthy dietary pattern to longevity-associated or Eatwell Guide for 40- and 70-year-old adults from the UK. We also present comparison from unhealthy patterns to the longevity-associated pattern and to that corresponding with the Eatwell Guide. Plots are made using the R package Highcharter.[22]

Ethical approval: This study was performed under generic ethical approval obtained by UK Biobank from the National Health Service National Research Ethics Service (approval letter ref 11/NW/0382, 17 June 2011).

The UK Biobank Study: The UK Biobank is a prospective cohort study which recruited >500,000 participants aged 37-73 years, 56% female, from 22 centres across the UK (England, Scotland, and Wales) between 2006 and 2010. The UK Biobank was conducted in accordance with the Declaration of Helsinki and was approved by the North-West Multi-Centre Research Ethics Committee (REC reference: 12/NW/03820). At the baseline study visit at an assessment centre, a touchscreen questionnaire was used to collect data on sociodemographic factors, diet, and general health, amongst others, blood samples were collected, and anthropometric measurements made, as described below.

Dietary assessment:

Food Frequency questionary: The baseline touchscreen questionnaire that was completed by participants at the assessment centre, was based on the food frequency questionnaire (FFQ), which included 29 items about diet and 18 questions about alcohol.[23] The questionnaire included data on the frequency of consumption of the main food groups over the previous year, including fruits and vegetables, fish, meat, and cheese (details in Table S4). A 24-hour recall-based method, based on the Oxford WebQ, which captured information on up to 206 food and 32 drink items, was introduced towards the end of the recruitment period. Thus, participants recruited between April 2009 and September 2010 completed this at their assessment centre baseline visit. In addition, between February 2011 and June 2012, there were 4 cycles, separated by 3-4 months, where participants were invited via email to complete the 24-hour dietary recall at home. Over 200,000 participants completed at least one 24-hour recall. Further details about the dietary assessments, including reproducibility and agreement between the two methods have been published. In the present study, we used data from the 24-hour recall (Oxford WebQ) to assess adherence to the recommendations on the intakes of nuts, eggs, and sugar-sweetened drinks. We used FFQ data for fruits and vegetables, whole and refined grain, fish, diary, proceed and unprocessed meat and vegetables oils recommendations where the cut-offs are described as intake 'per week' so as not to over or

underestimate consumption of these foods. People who completed more than one questionary had similar characteristics related sex and age. However, those are more educated.

Processing of 24-hour recall data: For operationalisation of the recommendations using 24-hour recall data, intakes were derived by taking the means of the completed recalls. We excluded any recalls for which the participant answered 'no' to the following question "*Would you say that what you ate and drank yesterday was fairly typical for you?* (data field 100020). We also excluded any dietary recalls with extreme energy intakes (based on the 'Estimated Nutrients' data field 100002), using the cut-offs described by Perez-Cornago et al. (2021);[24] < 3347 or > 17,573 kJ per day (< 800 or > 4200 kcal/ per day) for men and <2092 or > 14,644 kJ per day (< 600 or > 3500 kcal per day) for women. Furthermore, as a sensitivity analysis, we excluded participants who had completed less than two dietary recalls. Perez-Cornago et *al*. advise that at least two 24-hour recall assessments are used, if possible, when investigating diet-disease associations, as a single dietary assessment is unlikely to reflect habitual intakes. Standard portion sizes derived from the UK Nutrient Databank, which includes food composition data most relevant to the time when UK Biobank participants completed the dietary assessments, were used, as described by Perez-Cornago et al. We used the frequency data and standard portion sizes for each food item to calculate the mean intake in grams per day and summed these to create a total intake of fruits and vegetables in grams per day. Where standard portion sizes were not defined for 'Vegetable pieces' (data field 104070), we allocated this portion as 60g, which is the same as a standard portion of 'Other vegetables' (data field 104380).

Dietary variables
Generally, we grouped participants into four categories according to the intake of each food/food group. The cut-points for the categories were whole integers, chosen to have reasonable and similar numbers of participants in each group, as far as the distribution of data allowed.

Meat and fish: For oily fish, non-oily fish, processed meats, poultry, beef, lamb, pork, participants were asked how often each item was consumed with possible answers being: ' never, 'less than once a week', 'once a week', '2-4 times a week', '5-6 times a week', 'once or more daily', 'do not know', 'prefer not to answer'. For processed meat, poultry, oily fish, and non-oily fish, we combined the top three frequencies to get four categories: never, <1.0 time per week, 1.0 time per week, and ⩾2.0 times per week'. To rank the participants by weekly red meat consumption based on the touchscreen, we summed the frequencies for beef, pork, and lamb/mutton, using the following coding: 'Never' = 0, 'Less than once a week' = 0.5, ' Once a week' = 1, '2-4 times a week' = 3, '5-6 times a week' = 5.5, 'Once or more daily' = 7. The four categories for red meat consumption were: <1 time per week, 1.0-1.9 times per week, 2.0-2.9 times per week, and ⩾3.0 times per week. For red and processed meat, we summed the frequencies for beef, pork, lamb/mutton, and processed meat. The categories for red and processed meat consumption were: < 2.0 times per week, 2.0-2.9 times per week, 3.0-3.9 times per week, and ⩾4.0 times per week. To rank the participants by weekly total fish consumption based on the touchscreen, we summed the frequencies for oily fish and non-oily fish and grouped participants into the following categories: <1.0 time per week, 1.0-1.9 times per week, 2.0-2.9 times per week, and ⩾3.0 times per week.

Fruit: For fruit, participants were asked to directly enter the number of pieces of fresh fruit and dried fruit (with examples given as to what constitutes a piece eaten per day) or select

‘less than one’, ‘do not know’ or ‘prefer not to answer’. One piece of fresh fruit, and two ‘pieces’ of dried fruit were counted as servings. We grouped participants into the following categories: <2.0 servings per day, 2.0-2.9 servings per day, 3.0-3.9 servings per day, and ⩾ 4.0 servings per day.

Vegetables: For vegetables, participants were asked to directly enter the number of heaped tablespoons of cooked vegetables and salad/raw vegetables eaten per day or select ‘less than one’, ‘do not know’ or ‘prefer not to answer’. Two heaped tablespoons of vegetables were counted as a serving. We grouped participants into the following categories: <2.0 servings per day, 2.0-2.9 servings per day, 3.0-3.9 servings per day, and ⩾4.0 servings per day.

Milk: We derived an estimate of milk intake using the questions on type of milk, bowls of breakfast cereal, cups of tea, and cups of coffee. Participants were asked which type of milk they mainly use, and those who answered ‘Never/rarely have milk’ were assigned to the first category. For the participants who selected one of ‘Full cream’, ‘Semi-skimmed’ or ‘Skimmed’, we estimated their total milk consumption by summing up their daily consumption of milk, assuming that participants added 100 mL of milk to each bowl of breakfast cereal, 35 mL of milk to each cup of tea, and 25 mL of milk to each cup of coffee. These participants were then divided into three categories: those that consumed <150 mL of milk, 150-299 mL of milk, and ⩾300 mL of milk daily. A limitation of the estimated milk intake is that some people may not add milk to tea or coffee, but the touchscreen did not ask about this. To determine whether this derived estimate of milk intake was able to discriminate between participants with low and high milk intakes, we used the sub-sample of participants who completed at least one 24-hour dietary assessment. Within each category of milk consumption from the touchscreen questionnaire, we calculated the average intake of dairy milk from the 24-hour dietary assessments. This showed that the touchscreen categories of milk consumption were able to discriminate between those with low and high intakes (see Supplementary Table S4). We further calculated that within the 24-hour dietary assessments, for those that selected whole milk, semi-skimmed milk, or skim milk as the milk mainly used, 94 % of their total milk intake came from milk added to breakfast cereal, tea, and coffee (the remainder was from glasses of milk and milky drinks, e.g., hot chocolate, that were not asked about in the touchscreen questionnaire).

Added detailed on statistical methods:
Conceptually, our approach is as follows:

1) LE(MUK;age,sex) denotes the sex- and age-specific life expectancy of an individual adhering to the median baseline dietary pattern in the UK.
2) LE(D;age,sex) denotes the sex- and age-specific life expectancy of an individual adhering to a specific dietary pattern D (where D can be intake of any combination of food groups set to quintiles per food group of the population in UK).
3) Median dietary patterns were set to the mid quintile intakes. For the unhealthy eating pattern, we used the quintiles for each food group with the highest mortality association and inversely for the longevity-associated dietary patterns, we used the quintiles for each food group with the lowest mortality association. For the Eatwell Guide, we chose the quintiles of daily intakes fitting best with the Eatwell Guide.
4) LE(D;age,sex) and LE(MUK;age,sex) are calculated using standard lifetable methodology, where each dietary pattern is associated with a set of sex- and age-specific annual mortality rates.
5) Life years gained (or lost) due to a sustained change from the baseline dietary pattern to the new dietary pattern D is LE(D;age,sex)-LE(MUK;age,sex).

LE(MUK;age,sex) is calculated using average sex- and age-specific mortality rates for the UK obtained from the Global Burden of Diseases and Injuries study (GBD).[10] To calculate LE(D;age,sex), we multiply the sex- and age-specific mortality rates associated with median dietary patterns in the UK by hazard ratios associated with moving from median dietary patterns in the UK to diet pattern D. These hazard ratios on dietary risk factors were obtained from UK Biobank and adjusted for age, sex, socio-demographic area, smoking, alcohol consumption, and activity level (see Table S1).

To account for the expected time delay between dietary change and the development of diseases such as cardiovascular disease and cancer,[11-13] while weighting in the morbidity burden,[14-18] we assumed that time to full effect was 20 years with an inverse S-shaped decrease in mortality (Figure S1). We also conducted sensitivity analyses with delays of 5 years to 50 years for a range of different S-shaped curves.

To calculate uncertainty intervals (UI) for the overall, and food specific, associations between dietary change with life expectancy, we considered the confidence intervals for the hazard ratios for each food group from the UK Biobank data. Then, we drew a number at random within the 95% confidence interval for each food group, and used this as the model input. This procedure was repeated 200 times and 95% uncertainty limits were selected as the 2.5- and 97.5 percentiles of the distribution of the 200 replicates.

Model adjustments: Food intake and associations to mortality is correlated between food groups. Not accounting the hazard ratios for these correlations, may give biased results. To account for this, our model adjust hazard ratios (HR_a) by a model parameter (m, ranging from 0 to 1.0 where m=1 means that hazard ratios (HR) for all food groups are assumed independent and m=0.5 means that hazard ratios (HR_0) will be moved half-way towards 1.

When $HR_0<1$: $HR_a = HR_0 + (1- HR_0)*(1-m)$, for $HR_0>1$: $HR_a = HR_0 + (1- HR_0)\times (1-m)$).

We calculated both core-adjusted and mutually adjusted hazard ratios for the food groups. The core-adjusted hazard ratios for mortality for each food group were adjusted for age, sex, socio-demographic area, smoking, alcohol consumption, and activity level. We assess potential mediation and/or confounding between the food groups and mortality by body mass index and energy, we also present models also adjusting for body mass index and energy intake in addition to the adjustment in the core models. To reduce random variation, the hazard ratios for the food groups were not stratified by age and sex groups. The mutually adjusted hazard ratios were adjusted additionally for other important food groups (fruits, vegetables, nuts, red- and processed meats). The mutually adjusted hazard ratios corresponded to a core-adjusted hazard ratios with an m ranging from 0.83 to 0.88. To account for correlation between all food group categories, core-adjusted hazard ratios with an m=0.75 were used (presented in sensitivity analyses). However, to be more conservative in our main estimates and to allow for some residual confounding in the data, we used m=0.5 in our main analyses and present sensitivity analyses for m=0.25 (highly conservative estimates), m=0.75 (adjusted without residual confounding), and m=1.0 (assuming no overlap between food groups, Table S3).

Limitations and strengths

Our study has several strengths and some limitations. First, we used a large cohort study from the UK with available food intake and mortality data, UK Biobank, supplemented with data

from the Global Burden of Disease. Dietary changes can probably contribute to improvements in health outcomes such as type 2 diabetes relatively quickly,[25,26] but more slowly for diseases such as cancer.[13,27] To balance between these, we assumed the improvements in health that contribute to increased life expectancy plateau at 20-year since after the sustained change in dietary patterns is initiated. This assumption involves uncertainty, but we conducted sensitivity analyses for a 5-to-50-year range with overall directions of change similar. Associations between dietary variables and associations with mortality were not stratified by age and sex, which limits the possibility to detect potential effect modifications by age and sex on dietary patterns and mortality relationships. However, stratified analyses would introduce substantial random variability and loss in precision. It is also worth to mention that we model the assumption of sustained dietary changes. Maintaining lifestyle changes over time including dietary improvements can be challenging, and for many dietary patterns fluctuate over time. We have not modelled potential changes in life expectancy of fluctuating changes. The UK Biobank does not measure consumption of rice, the most widely consumed food in the world, and proportionally consumed at greater level in people non-white ethnicity backgrounds. Overall, the UK Biobank data underrepresents non-white in the sampled cohort when compared to UK population.

Our background mortality data is mostly based on pre-COVID-19 data to avoid excess mortality due to the initial waves of COVID-19. However, since mortality data were used until June 2020 that some cases of mortality could be due to COVID (March -June 2020). Due to the substantial COVID-19 restrictions in this period limiting transmissions of COVID-19, we assume the impact of COVID-19 in our data is minimal. Differences in energy intake may contribute to the predicted differences in life expectancy because healthier diets reduce the risk of obesity/overweight,[28] and this could be a mediating and/or confounding factor for life expectancy gains. It is likely that the predicted increases in life expectancy will be associated with improvement in health-related quality of life.[14-16,28-31 32] The quality of evidence for change in outcomes following change in intake of most of the food groups is likely to be moderate.[33] Further, since foods are not eaten in isolation but in meals and as part of dietary patterns, the intakes of specific food groups are correlated. We addressed this issue through model adjustments designed to limit potential over-estimation of the health gains.[34-36] As for most cohort studies, confounders could impact. However, we have adjusted our core models for several potential confounders such as age, sex, area-based socio-demographic deprivation, smoking, alcohol consumption, and physical activity level. Some residual confounding and bias may affect the estimates, but we presented conservative estimates as our main findings and thus overestimation is less likely. We have also assessed correlation between food groups with comparing Cox regression analyses with mutually adjusting for other food groups. To avoid collider effects/bias, we used the core models as the main analyses. The risks and susceptible at different age groups differ with different morbidity burdens at different ages. This is to some extent taken into account by using age-specific background mortality data, but the food associations to mortality could be different for different disease burdens and this is not taken into account. Participants in the UK Biobank are healthier and less socioeconomically deprived than the UK population,[37] but risk factor associations in the UK Biobank are likely to be generalisable to the UK population.[4] Finally, our estimates should be seen as population estimates and not individual forecasting.

Sensitivity analyses

In sensitivity analyses, when increasing time-to-full-effect from 20 years to 50 years, gains in life expectancy were reduced by 19-22% for 40-year-olds and by 52-54% for 70-year-olds (Supplementary Table S5-6, Fig. S5-10). Changing the time-to-full-effect from 20 years to 5

years, increased gains in life expectancy by 5-6% for 40-year-olds and by 37-46% for 70-year-olds. Changing model adjustments from m=0.5 to m=0.25, the estimates of life expectancy gains were reduced by 50-51%, while changing model adjustments from m=0.5 to 0.75, the estimates were increased by 46-52%. Sex-stratified analyses on food groups and associations to mortality (Table S9) generally showed similar associations across sex except white meat which seemed to be more beneficial among females. To reduce the potential contribution of reverse causation to the data, we performed a landmark analysis that excluded events that occurred within the previous two years. The data could be influenced by measurement errors, including social desirable reporting and recall difficulties.[38] Recall difficulties are reduced by focusing on habitual intakes and relatively short recall periods (see details in descriptions above). Different age groups also have different susceptibility to various diseases and could also have different dietary patterns. However, our analysis structure should to large degree reduce potential susceptibility bias.

Data sharing: No additional data available. Any data request must be made to UK Biobank.

Dissemination to participants and related patient and public communities: Results of the study will be linked in the UK Biobank website for research participants and relevant patient and public communities.

References

1. Petermann-Rocha, F.*, et al.* Nonlinear Associations Between Cumulative Dietary Risk Factors and Cardiovascular Diseases, Cancer, and All-Cause Mortality: A Prospective Cohort Study From UK Biobank. *Mayo Clin Proc* **96**, 2418-2431 (2021).
2. Greenwood, D.C.*, et al.* Validation of the Oxford WebQ Online 24-Hour Dietary Questionnaire Using Biomarkers. *American journal of epidemiology* **188**, 1858-1867 (2019).
3. Liu, B.*, et al.* Development and evaluation of the Oxford WebQ, a low-cost, web-based method for assessment of previous 24 h dietary intakes in large-scale prospective studies. *Public health nutrition* **14**, 1998-2005 (2011).
4. Batty, G.D., Gale, C.R., Kivimaki, M., Deary, I.J. & Bell, S. Comparison of risk factor associations in UK Biobank against representative, general population based studies with conventional response rates: prospective cohort study and individual participant meta-analysis. *Bmj* **368**, m131 (2020).
5. Scheelbeek, P.*, et al.* Health impacts and environmental footprints of diets that meet the Eatwell Guide recommendations: analyses of multiple UK studies. *BMJ Open* **10**, e037554 (2020).
6. Townsend, P. & A., B. Health and deprivation. Inequality and the North. *Health Policy (New York)* **10**(1988).
7. Guo, W., Bradbury, K.E., Reeves, G.K. & Key, T.J. Physical activity in relation to body size and composition in women in UK Biobank. *Ann Epidemiol* **25**, 406-413 e406 (2015).
8. Johansson, K.A.*, et al.* Estimating Health Adjusted Age at Death (HAAD). *PloS one* **15**(2020).
9. Fadnes, L.T., Økland, J.-M., Haaland, Ø.A. & Johansson, K.A. Estimating impact of food choices on life expectancy: A modeling study. *PLoS Med* **19**, e1003889 (2022).
10. The Institute for Health Metrics and Evaluation (IHME). The Global Health Data Exchange (GHDx) Vol. 2020 (Institute for Health Metrics and Evaluation, University of Washington,).
11. Capewell, S. & O'Flaherty, M. Can dietary changes rapidly decrease cardiovascular mortality rates? *Eur Heart J* **32**, 1187-1189 (2011).
12. Capewell, S. & O'Flaherty, M. Rapid mortality falls after risk-factor changes in populations. *Lancet* **378**, 752-753 (2011).
13. Wang, Y.*, et al.* Fruit and vegetable consumption and risk of lung cancer: a dose-response meta-analysis of prospective cohort studies. *Lung Cancer* **88**, 124-130 (2015).

14. Aune, D., *et al.* Fruit and vegetable intake and the risk of cardiovascular disease, total cancer and all-cause mortality-a systematic review and dose-response meta-analysis of prospective studies. *Int J Epidemiol* **46**, 1029-1056 (2017).
15. Aune, D., *et al.* Whole grain consumption and risk of cardiovascular disease, cancer, and all cause and cause specific mortality: systematic review and dose-response meta-analysis of prospective studies. *Bmj* **353**, i2716 (2016).
16. Aune, D., *et al.* Nut consumption and risk of cardiovascular disease, total cancer, all-cause and cause-specific mortality: a systematic review and dose-response meta-analysis of prospective studies. *Bmc Med* **14**, 207 (2016).
17. Schwingshackl, L., *et al.* Food groups and risk of all-cause mortality: a systematic review and meta-analysis of prospective studies. *The American journal of clinical nutrition* **105**, 1462-1473 (2017).
18. Collaborators, G.B.D.C.o.D. Global, regional, and national age-sex-specific mortality for 282 causes of death in 195 countries and territories, 1980-2017: a systematic analysis for the Global Burden of Disease Study 2017. *Lancet* **392**, 1736-1788 (2018).
19. Mathers, C.D., *et al.* Sensitivity and Uncertainty Analyses for Burden of Disease and Risk Factor Estimates. in *Global Burden of Disease and Risk Factors* (eds. Lopez, A.D., Mathers, C.D., Ezzati, M., Jamison, D.T. & Murray, C.J.L.) (Washington (DC)
New York, 2006).
20. R Core Team. R: A language and environment for statistical computing. R Foundation for Statistical Computing. (Vienna, Austria. , 2021).
21. Chang, W., *et al.* R Shiny Application Framework for R. R package version 1.7.1. (2021).
22. Kunst, J. Highcharter: A Wrapper for the 'Highcharts' Library. R package version 0.8.2. (2020).
23. Bradbury, K.E., Young, H.J., Guo, W. & Key, T.J. Dietary assessment in UK Biobank: an evaluation of the performance of the touchscreen dietary questionnaire. *J Nutr Sci* **7**, e6 (2018).
24. Perez-Cornago, A., *et al.* Description of the updated nutrition calculation of the Oxford WebQ questionnaire and comparison with the previous version among 207,144 participants in UK Biobank. *Eur J Nutr* **60**, 4019-4030 (2021).
25. Tuomilehto, J., *et al.* Prevention of type 2 diabetes mellitus by changes in lifestyle among subjects with impaired glucose tolerance. *The New England journal of medicine* **344**, 1343-1350 (2001).
26. Lean, M.E., *et al.* Primary care-led weight management for remission of type 2 diabetes (DiRECT): an open-label, cluster-randomised trial. *Lancet* **391**, 541-551 (2018).
27. Lee, P.N., Forey, B.A. & Coombs, K.J. Systematic review with meta-analysis of the epidemiological evidence in the 1900s relating smoking to lung cancer. *BMC Cancer* **12**, 385 (2012).
28. Schlesinger, S., *et al.* Food Groups and Risk of Overweight, Obesity, and Weight Gain: A Systematic Review and Dose-Response Meta-Analysis of Prospective Studies. *Advances in nutrition* **10**, 205-218 (2019).
29. Schwingshackl, L., Hoffmann, G., Iqbal, K., Schwedhelm, C. & Boeing, H. Food groups and intermediate disease markers: a systematic review and network meta-analysis of randomized trials. *American Journal of Clinical Nutrition* **108**, 576-586 (2018).
30. Schwingshackl, L., *et al.* Food groups and risk of type 2 diabetes mellitus: a systematic review and meta-analysis of prospective studies. *European Journal of Epidemiology* **32**, 363-375 (2017).
31. Schwingshackl, L., *et al.* Food groups and risk of colorectal cancer. *International Journal of Cancer* **142**, 1748-1758 (2018).
32. Public Health England. Chapter 1: life expectancy and healthy life expectancy in *Health profile for England: 2017* (Public Health England, , London, 2017).

3. Schwingshackl, L., *et al.* Perspective: NutriGrade: A Scoring System to Assess and Judge the Meta-Evidence of Randomized Controlled Trials and Cohort Studies in Nutrition Research. *Advances in nutrition* **7**, 994-1004 (2016).
34. Roswall, N., *et al.* Adherence to the healthy Nordic food index and total and cause-specific mortality among Swedish women. *Eur J Epidemiol* **30**, 509-517 (2015).
35. Vormund, K., *et al.* Mediterranean diet and mortality in Switzerland: an alpine paradox? *Eur J Nutr* **54**, 139-148 (2015).
36. Prinelli, F., *et al.* Mediterranean diet and other lifestyle factors in relation to 20-year all-cause mortality: a cohort study in an Italian population. *Br J Nutr* **113**, 1003-1011 (2015).
37. Fry, A., *et al.* Comparison of Sociodemographic and Health-Related Characteristics of UK Biobank Participants With Those of the General Population. *American journal of epidemiology* **186**, 1026-1034 (2017).
38. Fadnes, L.T., Taube, A. & Tylleskar, T. How to identify information bias due to self-reporting in epidemiological research. *The Internet Journal of Epidemiology* **7**(2009).

Text S3: Data analysis code in R.

```
## Food-app

# Importing food input data in quintiles into haz, lci, uci (extracted through: "UK extraction of
values" and then converting dots to commas from "haz-dot" and saving as "haz2.csv" etc)

## Necessary packages
packages <-
c("shiny","ggplot2","R.utils","R.devices","tibble","shinycssloaders","fmsb","data.table","high
charter")
# install.packages("highcharter", dependencies = TRUE)
# install.packages("fmsb", dependencies = TRUE)
library("highcharter")
## Installing packages that are not already installed
new.packs <- which(!(packages%in%installed.packages()));new.packs
if(length(new.packs)>0) install.packages(packages[new.packs])

## Load necessary packages. Must be included to ensure that app runs when deposited
lapply(X = packages, FUN = function(x){
  try(library(x, character.only = T))
})

if(F) rm(list = ls(all.names = T))

set.seed(seed = 260973)
n.ageint<-100
source("EX_RISK.R")
# load("demo_pop_gbd2017.rda")
load("epi_cond_gbd2019.rda")
source("forest.R")

# energy_per<-c(4,2,1.5,26,5,6,6,1.7,11,7,19,10,2,37) # kJ/g
# names(energy_per)<-
c("wholegrains","veg","fruit","nuts","legumes","fish","egg","milk","refinedgrains","meat_red
","meat_processed","meat_white","ssb","oil")

nutrigrade<-c(8,5.8,5.8,7,6,7.75,3.8,6,5,6.5,7.5,2,5.5)
names(nutrigrade)<-
c("wholegrains","veg","fruit","nuts","legumes","fish","egg","milk","refinedgrains","meat_red
","meat_processed","meat_white","ssb")

sliderInput2 <- function(inputId, label, min, max, value, step=NULL, from_min, from_max){
  x <- sliderInput(inputId, label, min, max, value, step)
  x$children[[2]]$attribs <- c(x$children[[2]]$attribs,
                               "data-from-min" = from_min,
                               "data-from-max" = from_max,
                               "data-from-shadow" = TRUE)
  x
}
```

```
## ui is a necessary function giving the user interface
ui<-fluidPage(tags$head(
  tags$link(rel = "shortcut icon", type = "image/png", href = "scl-bceps.png"),
  tags$title("Food4healthylife")
),
## Padding to compensate for fixed-top
tags$style(type="text/css", "body {padding-top: 70px}"),
## Example from: C:\Users\oeh041\OneDrive - University of Bergen\Shiny\Test6
tags$style(HTML(".navbar{background-color: #CF1717}
                .navbar-default .navbar-nav > .active > a,
                .navbar-default .navbar-nav > .active > a:focus,
                .navbar-default .navbar-nav > .active > a:hover {color: black;background-color:
#F8A88D}")),
navbarPage(title = tags$div("Food4HealthyLifeUK"),
           theme="sandstone.css",
           position = "fixed-top",

           mainPanel(
             tabsetPanel(
               ##### Calculator
               tabPanel("Calculator",
                        fluidRow(
                          column(width=3,selectInput("country", "Location",
unique(gbd.both$location_name), multiple=F, selectize=F, selected = "United Kingdom")),
                          column(width=3,selectInput("age", "Age", 20:80, multiple=F, selectize=F,
selected = 40)),
                          column(width=4,radioButtons(inputId = "sex",label = "Gender", choices =
c("Female","Male","Both"), inline=T)),
                        ),
                        fluidRow(
                          column(width=3,actionButton(inputId = "optimal.button",label = "New diet:
Healthiest")),
                          column(width=4,actionButton(inputId = "eatwell.button",label = "New diet:
Eatwell")),
                          column(width=5,actionButton(inputId = "unhealthy.button",label = "Current
diet: Unhealthy"))
                        ),
                        fluidRow(column(12,withSpinner(highchartOutput("forest",width =
"100%")))),
                        hr(),

fluidRow(column(4,textOutput("LE")),column(4,textOutput("LE2")),column(4,textOutput("L
E.diff"))),

#fluidRow(column(4,textOutput("LE")),column(4,textOutput("LE2")),column(4,textOutput("
LE.diff"),column(4,textOutput("hazratio"))),
                        hr(
```

```
                fluidRow(column(3,sliderInput("wholegrains","Whole grains (fresh weight, 
before, g):",min=1,max=5,value=3,step=1)),column(3,sliderInput("wholegrains2","Whole 
grains (fresh weight, after, g):",min=1,max=5,value=3,step=1)),column(6,"")),
                fluidRow(column(3,sliderInput2("fish","Fish (before, 
g):",min=1,max=5,value=3,step=1,from_min = 1,from_max = 
5)),column(3,sliderInput2("fish2","Fish (after, g):",min=1,max=5,value=3,step=1,from_min = 
1,from_max = 5)),column(6,"")),
                fluidRow(column(3,sliderInput2("meat_processed","Processed meat (before, 
g):",min=1,max=5,value=3,step=1,from_min = 1,from_max = 
5)),column(3,sliderInput2("meat_processed2","Processed meat (after, 
g):",min=1,max=5,value=3,step=1,from_min = 1,from_max = 5)),column(6,"")),
                fluidRow(column(3,sliderInput2("nuts","Nuts (before, 
g):",min=1,max=5,value=3,step=1,from_min = 1,from_max = 
5)),column(3,sliderInput2("nuts2","Nuts (after, g):",min=1,max=5,value=3,step=1,from_min 
= 1,from_max = 5)),column(6,"")),
                fluidRow(column(3,sliderInput2("meat_red","Red meat (before, 
g):",min=1,max=5,value=3,step=1,from_min = 1,from_max = 
5)),column(3,sliderInput2("meat_red2","Red meat (after, 
g):",min=1,max=5,value=3,step=1,from_min = 1,from_max = 5)),column(6,"")),
                fluidRow(column(3,sliderInput2("legumes","Legumes (before, 
g):",min=1,max=5,value=3,step=1,from_min = 1,from_max = 
5)),column(3,sliderInput2("legumes2","Legumes (after, 
g):",min=1,max=5,value=3,step=1,from_min = 1,from_max = 5)),column(6,"")),
                fluidRow(column(3,sliderInput("milk","Milk/dairy (before, 
g):",min=1,max=5,value=3,step=1)),column(3,sliderInput("milk2","Milk/dairy (after, 
g):",min=1,max=5,value=3,step=1)),column(6,"")),
                fluidRow(column(3,sliderInput("veg","Vegetables (before, 
g):",min=1,max=5,value=3,step=1)),column(3,sliderInput("veg2","Vegetables (after, 
g):",min=1,max=5,value=3,step=1)),column(6,"")),
                fluidRow(column(3,sliderInput("fruit","Fruit (before, 
g):",min=1,max=5,value=3,step=1)),column(3,sliderInput("fruit2","Fruit (after, 
g):",min=1,max=5,value=3,step=1)),column(6,"")),
                fluidRow(column(3,sliderInput("ssb","Sugar sweetened beverages (before, 
g):",min=1,max=5,value=3,step=1)),column(3,sliderInput("ssb2","Sugar sweetened beverages 
(after, g):",min=1,max=5,value=3,step=1)),column(6,"")),
                fluidRow(column(3,sliderInput2("refinedgrains","Refined grains (before, 
g):",min=1,max=5,value=3,step=1,from_min = 1,from_max = 
5)),column(3,sliderInput2("refinedgrains2","Refined grains (after, 
g):",min=1,max=5,value=3,step=1,from_min = 1,from_max = 5)),column(6,"")),
                fluidRow(column(3,sliderInput2("egg","Eggs (before, 
g):",min=1,max=5,value=3,step=1,from_min = 1,from_max = 
5)),column(3,sliderInput2("egg2","Eggs (after, g):",min=1,max=5,value=3,step=1,from_min 
= 1,from_max = 5)),column(6,"")),
                fluidRow(column(3,sliderInput2("meat_white","White meat (before, 
g):",min=1,max=5,value=3,step=1,from_min = 1,from_max = 
5)),column(3,sliderInput2("meat_white2","White meat (after, 
g):",min=1,max=5,value=3,step=1,from_min = 1,from_max = 5)),column(6,"")),
                #  fluidRow(column(3,sliderInput2("oil","Added oils (before, 
g):",min=1,max=5,value=3,step=1,from_min = 1,from_max = 
```

```
5)),column(3,sliderInput2("oil2","Added oils (after,
g):",min=1,max=5,value=3,step=1,from_min = 1,from_max = 5)),column(6,"")),
                fluidRow(column(12,textOutput("nutrigrade")))
              )
        ),
        tabPanel("Help",
              fluidRow(column(12,htmlOutput("Help")))),
        tabPanel("Settings",
              fluidRow(
                column(width=3,sliderInput2("m","Model adjustment (conservative vs.
full):",min=0,max=1.0,value=0.5,step=.01, from_min = 0.25, from_max = 1.0)),
                column(width=3,sliderInput("delay","Time to full effect
(years):",min=0,max=50,value=20,step=1)),
                column(width=1,radioButtons(inputId = "mutuallyadjusted",label =
"Adjusted data", choices = c("Core","Extended"),selected = "Core", inline=T)),
                column(width=1,actionButton(inputId = "standardmodel",label = "Standard
model")),
                column(width=1,actionButton(inputId = "extensivemodel",label = "Extensive
model")),
                column(width=1,downloadButton("downloadData","Download"))
              )
        ),
        # tabPanel("Morbidity",
        #   # fluidRow(column(12,plotOutput("radar",width = "60%"))),
        #   # fluidRow(column(width=1,downloadButton("savePlot","DownloadPNG"))),
        #   #fluidRow(column(width=1,downloadButton("savePlot2","DownloadEPS"))),
        #   # fluidRow(column(12,withSpinner(highchartOutput("radar",width = "60%"))))
        # ),
        tabPanel("Citation",
              fluidRow(column(12,htmlOutput("Citation"))))
      )
    )
)
)

## server is necessary for doing computations
server<-function(input,output,session){
  data<-reactive({
    age<-as.numeric(input$age)
    country<-input$country
    m<-input$m
    delay <- input$delay
    if(input$sex=="Both"){gbd<-gbd.both}
    if(input$sex=="Male"){gbd<-gbd.male}
    if(input$sex=="Female"){gbd<-gbd.female}
    allcauses <- copy(gbd[location_name==country & cause_name=="All causes"])
    h<-factor(allcauses$age_name,levels=c(c("<1 year", "1-4 years", "5-9 years", "10-14
years", "15-19 years","20-24 years","25-29 years","30-34 years","35-39 years","40-44
years","45-49 years","50-54 years","55-59 years","60-64 years","65-69 years","70-74
years","75-79 years","80-84 years","85-89 years","90-94 years","95+ years")))
```

```
  allcauses<-allcauses[order(h)]
  qx<-read2.qx(lifetab=allcauses$Deaths,n.ageint=n.ageint)

  food.names <- c("wholegrains", "veg", "fruit", "nuts", "legumes", "fish", "egg", "milk",
"refinedgrains", "meat_red", "meat_processed", "meat_white", "ssb")
  uk.defaults<-c(3,3,3,3,3,3,3,3,3,3,3,3,3)
  #uk.defaults<-c(3,3,3,3,3,3,3,3,3,3,3,3,3,3)
  defaults<-uk.defaults
  names(defaults)<-food.names
  wholegrains<-input$wholegrains
  wholegrains2<-input$wholegrains2
  veg<-input$veg
  veg2<-input$veg2
  fruit<-input$fruit
  fruit2<-input$fruit2
  nuts<-input$nuts
  nuts2<-input$nuts2
  legumes<-input$legumes
  legumes2<-input$legumes2
  fish<-input$fish
  fish2<-input$fish2
  egg<-input$egg
  egg2<-input$egg2
  milk<-input$milk
  milk2<-input$milk2
  refinedgrains<-input$refinedgrains
  refinedgrains2<-input$refinedgrains2
  meat_red<-input$meat_red
  meat_red2<-input$meat_red2
  meat_processed<-input$meat_processed
  meat_processed2<-input$meat_processed2
  meat_white<-input$meat_white
  meat_white2<-input$meat_white2
  ssb<-input$ssb
  ssb2<-input$ssb2
  # oil<-input$oil
  # oil2<-input$oil2

  ## For debugging
  if(F){
    # cil.refinedgrains.rel
    # [1] 1.005708
    # > ciu.refinedgrains.rel
    # [1] 0.9508617
    country<-"United Kingdom"
    gbd<-gbd.both
    age<-40
    # m <- 1
    m<-0.5
    delay <- 20
```

```
    # anchor<-delay
    # wm<-1;alpha<-0
    # time <- 0:anchor;time
    # beta <- 0.2125
    # gamma<-(-1/beta);gamma
    # c <- beta*(alpha-wm)*(exp(anchor*beta+1))/(beta*(anchor-
gamma)*exp(gamma*beta+1)-exp(anchor*beta));c
    # vdelay <- fweight(time=time,anchor=anchor,a=alpha,b=gamma,c=c,d=beta)
    ## Before change
    wholegrains = 3; veg = 3; fruit = 3; nuts = 3; legumes = 3;
    fish = 3; egg = 3; milk = 3; refinedgrains = 3; meat_red = 3;
    meat_processed = 3; meat_white = 3; ssb = 3
    #; oil = 3
    ## After change
    wholegrains2 = 3; veg2 = 3; fruit2 = 3; nuts2 = 3; legumes2 = 3;
    fish2 = 3; egg2 = 3; milk2 = 3; refinedgrains2 = 3; meat_red2 = 3;
    meat_processed2 = 3; meat_white2 = 3; ssb2 = 3
    #; oil2 = 3
  }

  h<-read.csv2("haz3.csv")
  h2<-read.csv2("haz3a.csv")
  if(input$mutuallyadjusted=="Core") {haz.table<-h[,2:6]}
  if(input$mutuallyadjusted=="Extended") {haz.table<-h2[,2:6]}
  if(input$mutuallyadjusted=="BMI") {haz.table<-h3a[,2:6]}
  if(input$mutuallyadjusted=="BMI&energy") {haz.table<-h3b[,2:6]}
  rownames(haz.table)<-food.names
  haz.table2<-haz.table

  lci<-read.csv2("lci3.csv")
  lci2<-read.csv2("lci3a.csv")
  #lci.table<-read.csv2("hazlciuci.csv")
  if(input$mutuallyadjusted=="Core") {lci.table<-lci[,2:6]}
  if(input$mutuallyadjusted=="Extended") {lci.table<-lci2[,2:6]}
  rownames(lci.table)<-rownames(haz.table)
  uci<-read.csv2("uci3.csv")
  uci2<-read.csv2("uci3a.csv")
  if(input$mutuallyadjusted=="Core") {uci.table<-uci[,2:6]}
  if(input$mutuallyadjusted=="Extended") {uci.table<-uci2[,2:6]}
  rownames(uci.table)<-rownames(haz.table)
  if(F){m<-1}
  # model adjustment
  for(i in 1:nrow(haz.table2)){
    for(j in 1:ncol(haz.table2)){
      if(!is.na(haz.table2[i,j])&haz.table2[i,j]<1){
        hr <- haz.table2[i,j]
        hr <- hr+(1-hr)*(1-m)
        haz.table2[i,j] <- hr
      }
      if(!is.na(haz.table2[i,j])&haz.table2[i,j]>1){
```

```
        hr <- 1/haz.table2[i,j]
        hr <- hr+(1-hr)*(1-m)
        haz.table2[i,j] <- 1/hr
      }
    }
  }

  for(i in 1:nrow(lci.table)){
    for(j in 1:ncol(lci.table)){
      if(!is.na(lci.table[i,j])&lci.table[i,j]<1){
        hr <- lci.table[i,j]
        hr <- hr+(1-hr)*(1-m)
        lci.table[i,j] <- hr
      }
      if(!is.na(lci.table[i,j])&lci.table[i,j]>1){
        hr <- 1/lci.table[i,j]
        hr <- hr+(1-hr)*(1-m)
        lci.table[i,j] <- 1/hr
      }
    }
  }

  for(i in 1:nrow(uci.table)){
    for(j in 1:ncol(uci.table)){
      if(!is.na(uci.table[i,j])&uci.table[i,j]<1){
        hr <- uci.table[i,j]
        hr <- hr+(1-hr)*(1-m)
        uci.table[i,j] <- hr
      }
      if(!is.na(uci.table[i,j])&uci.table[i,j]>1){
        hr <- 1/uci.table[i,j]
        hr <- hr+(1-hr)*(1-m)
        uci.table[i,j] <- 1/hr
      }
    }
  }


  cil<-function(x,y){lci.table[x,y]}
  ciu<-function(x,y){uci.table[x,y]}

  # if(sum(haz.table2<=uci.table,na.rm = T)!=324 | sum(lci.table<=haz.table2,na.rm = 
T)!=324) warning("lci > haz or haz > uci")

  haz<-function(x,y){haz.table2[x,y]}
  foods <- 
c(wholegrains,veg,fruit,nuts,legumes,fish,egg,milk,refinedgrains,meat_red,meat_processed,m
eat_white,ssb) #,oil)
```

```
    foods2 <-
c(wholegrains2,veg2,fruit2,nuts2,legumes2,fish2,egg2,milk2,refinedgrains2,meat_red2,meat_
processed2,meat_white2,ssb2) #,oil2)
    names(foods) <- names(foods2) <- rownames(haz.table)
    haz.tot2 <- haz.tot <- lci.tot<-lci.tot2<-uci.tot<-uci.tot2<-haz.defaults <- 1
    for(i in seq_along(defaults)){
      haz.defaults <- haz.defaults*haz(names(defaults)[i],paste0("X",1*defaults[i]))
      #print(haz(names(foods)[i],paste0("X",1*foods[i])))
    }
    for(i in seq_along(foods)){
      haz.tot <- haz.tot*haz(names(foods)[i],paste0("X",1*foods[i]))
      lci.tot <- lci.tot*cil(names(foods)[i],paste0("X",1*foods[i]))
      uci.tot <- uci.tot*ciu(names(foods)[i],paste0("X",1*foods[i]))
      #print(haz(names(foods)[i],paste0("X",1*foods[i])))
    }
    haz.tot<-haz.tot/haz.defaults
    lci.tot<-lci.tot/haz.defaults
    uci.tot<-uci.tot/haz.defaults
    for(i in seq_along(foods2)){
      haz.tot2 <- haz.tot2*haz(names(foods2)[i],paste0("X",1*foods2[i]))
      lci.tot2 <- lci.tot2*cil(names(foods2)[i],paste0("X",1*foods2[i]))
      uci.tot2 <- uci.tot2*ciu(names(foods2)[i],paste0("X",1*foods2[i]))
      #print(haz(names(foods)[i],paste0("X",1*foods[i])))
    }
    haz.tot2<-haz.tot2/haz.defaults
    lci.tot2<-lci.tot2/haz.defaults
    uci.tot2<-uci.tot2/haz.defaults

    # monte carlo run 200 times
    n.mc<-200
    ## Normal at log-scale, then transformed back to HR-scale
    mc<-function(n=n.mc,x,y){
      # set.seed(26032023)
      lcl <- log(lci.table[x,y])
      ucl <- log(uci.table[x,y])
      se <- (ucl-lcl)/(1.96*2)
      logHR <- ucl-1.96*se
      haz.vector <- exp(rnorm(n = n, mean = logHR, sd = se))
      haz.vector
    }
    ## Uniform between LCL and UCL
    # mc<-function(n=n.mc,x,y){
    #   haz.vector<-runif(n = n, min = lci.table[x,y], max = uci.table[x,y])
    #   haz.vector
    # }

    #    energy <-
as.numeric(c(wholegrains,veg,fruit,nuts,legumes,fish,egg,milk,refinedgrains,meat_red,meat_p
rocessed,meat_white,ssb,oil)%*%energy_per);#print(energy)
```

```
  #   energy2 <- 
as.numeric(c(wholegrains2,veg2,fruit2,nuts2,legumes2,fish2,egg2,milk2,refinedgrains2,meat
_red2,meat_processed2,meat_white2,ssb2,oil2)%*%energy_per);#print(energy2)
  n.ageint <- 100

  ## Managing delay
  ## Based on
  ## Haaland et al. (2019). A flexible formula for incorporating distributive concerns into 
cost-effectiveness analyses: Priority weights
  ## Adjusting for time since change in diet
  ## Mortality, M, for new diet, D_new, given time, t, since change in diet and age:
  ##
  ##   M(D_new|t,age) = w(t)*M(D_old|age) + (1-w(t))M(D_new|age)
  ##
  ## Setting max weight to 1
  wm<-1
  ## Anchors function to 0 at delay years
  anchor<-delay
  alpha<-0
  ## Time it takes from 0 (weight is 1) to anchor (weight is 0)
  time <- 0:anchor;time
  ## Finding the beta value that ensures that the weight is t/anchor after t years (i.e., when 
weight function crosses linear weight function)
  beta.func <- function(beta){
    time.cross <- anchor/5
    gamma<-(-1/beta)
    c <- beta*(alpha-wm)*(exp(anchor*beta+1))/(beta*anchor+1-exp(anchor*beta));c
    abs(fweight(time=time.cross,anchor=anchor,a=alpha,b=gamma,c=c,d=beta)-(1-
time.cross/anchor))
  }
  beta <- optim(par = 0.2, fn = beta.func)$par;beta
  ## Setting gamma and c
  gamma<-(-1/beta);gamma
  c <- beta*(alpha-wm)*(exp(anchor*beta+1))/(beta*(anchor-gamma)*exp(gamma*beta+1)-
exp(anchor*beta));c

  ## Weights for each year
  vdelay <- fweight(time=time,anchor=anchor,a=alpha,b=gamma,c=c,d=beta)

  LE.allcause <- 
EX_RISK(qx=mx2qx(qx),n.ageint=n.ageint,r=0,age=age,qual=NULL,interval=NULL,haz=1,
vdelay = vdelay)

  LE <- 
EX_RISK(qx=mx2qx(qx),n.ageint=n.ageint,r=0,age=age,qual=NULL,interval=NULL,haz=h
az.tot,vdelay = vdelay)
  LE2<- 
EX_RISK(qx=mx2qx(qx),n.ageint=n.ageint,r=0,age=age,qual=NULL,interval=NULL,haz=h
az.tot2,vdelay = vdelay)
  LE.diff<-LE2-LE;round(LE.diff,2)
```

```
## Testing ground
if(F){
  haz.mc<-mc(n = n.mc, x = "wholegrains", y = paste0("X",1*wholegrains))
  haz.mc2<-mc(n = n.mc, x = "wholegrains", y = paste0("X",1*wholegrains2))
  LE.diffs<-NULL
  ## XXX Added "vdelay = vdelay" in top row (haz.cm2). Why was that not there?
  for(i in 1:n.mc) LE.diffs<-append(LE.diffs,

EX_RISK(qx=mx2qx(qx),n.ageint=n.ageint,r=0,age=age,qual=NULL,interval=NULL,haz=haz.mc2[i]/haz.defaults, vdelay = vdelay)
                              -
EX_RISK(qx=mx2qx(qx),n.ageint=n.ageint,r=0,age=age,qual=NULL,interval=NULL,haz=haz.mc[i]/haz.defaults, vdelay = vdelay))
  LE.ci <- quantile(x = LE.diffs, probs = c(.025, .975))
  # LE.ci <- range(LE.diffs)
}

LE.wholegrains <-
EX_RISK(qx=mx2qx(qx),n.ageint=n.ageint,r=0,age=age,qual=NULL,interval=NULL,haz=haz("wholegrains",paste0("X",1*wholegrains))/haz("wholegrains",paste0("X",1*defaults["wholegrains"])), vdelay = vdelay)
LE.wholegrains2 <-
EX_RISK(qx=mx2qx(qx),n.ageint=n.ageint,r=0,age=age,qual=NULL,interval=NULL,haz=haz("wholegrains",paste0("X",1*wholegrains2))/haz("wholegrains",paste0("X",1*defaults["wholegrains"])), vdelay = vdelay)
LE.wholegrains.diff <- LE.wholegrains2[age]-LE.wholegrains[age]

# 1. Run n.mc for wholegrains
# 2. Run n.mc for wholegrains2
# 3. Estimate LE.diff for each run (usorted)
# 4. Get quantiles for LE.diff

set.seed(20230306)
haz.wholegrains.mc<-mc(n = n.mc, x = "wholegrains", y = paste0("X",1*wholegrains))
haz.wholegrains.mc2<-mc(n = n.mc, x = "wholegrains", y = paste0("X",1*wholegrains2))
LE.wholegrains.diffs<-NULL
for(i in 1:n.mc) LE.wholegrains.diffs <- append(LE.wholegrains.diffs,

EX_RISK(qx=mx2qx(qx),n.ageint=n.ageint,r=0,age=age,qual=NULL,interval=NULL,haz=haz.wholegrains.mc2[i]/haz("wholegrains",paste0("X",1*defaults["wholegrains"])), vdelay = vdelay)[age]
                                        -
EX_RISK(qx=mx2qx(qx),n.ageint=n.ageint,r=0,age=age,qual=NULL,interval=NULL,haz=haz.wholegrains.mc[i]/haz("wholegrains",paste0("X",1*defaults["wholegrains"])), vdelay = vdelay)[age])
LE.ci.wholegrains <- quantile(x = LE.wholegrains.diffs, probs = c(.025, .975))
# LE.ci.wholegrains <- range(LE.wholegrains.diffs)
```

```
  LE.veg<-
EX_RISK(qx=mx2qx(qx),n.ageint=n.ageint,r=0,age=age,qual=NULL,interval=NULL,haz=h
az("veg",paste0("X",1*veg))/haz("veg",paste0("X",1*defaults["veg"])), vdelay = vdelay)
  LE.veg2<-
EX_RISK(qx=mx2qx(qx),n.ageint=n.ageint,r=0,age=age,qual=NULL,interval=NULL,haz=h
az("veg",paste0("X",1*veg2))/haz("veg",paste0("X",1*defaults["veg"])), vdelay = vdelay)
  LE.veg.diff<-LE.veg2[age]-LE.veg[age]

  haz.veg.mc<-mc(n = n.mc, x = "veg", y = paste0("X",1*veg))
  haz.veg.mc2<-mc(n = n.mc, x = "veg", y = paste0("X",1*veg2))
  LE.veg.diffs<-NULL
  for(i in 1:n.mc) LE.veg.diffs<-append(LE.veg.diffs,

EX_RISK(qx=mx2qx(qx),n.ageint=n.ageint,r=0,age=age,qual=NULL,interval=NULL,haz=h
az.veg.mc2[i]/haz("veg",paste0("X",1*defaults["veg"])), vdelay = vdelay)[age]
                              -
EX_RISK(qx=mx2qx(qx),n.ageint=n.ageint,r=0,age=age,qual=NULL,interval=NULL,haz=h
az.veg.mc[i]/haz("veg",paste0("X",1*defaults["veg"])), vdelay = vdelay)[age])
  LE.ci.veg <- quantile(x = LE.veg.diffs, probs = c(.025, .975))
  # LE.ci.veg <- range(LE.veg.diffs)


  LE.fruit<-
EX_RISK(qx=mx2qx(qx),n.ageint=n.ageint,r=0,age=age,qual=NULL,interval=NULL,haz=h
az("fruit",paste0("X",1*fruit))/haz("fruit",paste0("X",1*defaults["fruit"])), vdelay = vdelay)
  LE.fruit2<-
EX_RISK(qx=mx2qx(qx),n.ageint=n.ageint,r=0,age=age,qual=NULL,interval=NULL,haz=h
az("fruit",paste0("X",1*fruit2))/haz("fruit",paste0("X",1*defaults["fruit"])), vdelay = vdelay)
  LE.fruit.diff<-LE.fruit2[age]-LE.fruit[age]

  haz.fruit.mc<-mc(n = n.mc, x = "fruit", y = paste0("X",1*fruit))
  haz.fruit.mc2<-mc(n = n.mc, x = "fruit", y = paste0("X",1*fruit2))
  LE.fruit.diffs<-NULL
  for(i in 1:n.mc) LE.fruit.diffs<-append(LE.fruit.diffs,

EX_RISK(qx=mx2qx(qx),n.ageint=n.ageint,r=0,age=age,qual=NULL,interval=NULL,haz=h
az.fruit.mc2[i]/haz("fruit",paste0("X",1*defaults["fruit"])), vdelay = vdelay)[age]
                              -
EX_RISK(qx=mx2qx(qx),n.ageint=n.ageint,r=0,age=age,qual=NULL,interval=NULL,haz=h
az.fruit.mc[i]/haz("fruit",paste0("X",1*defaults["fruit"])), vdelay = vdelay)[age])
  LE.ci.fruit <- quantile(x = LE.fruit.diffs, probs = c(.025, .975))
  # LE.ci.fruit <- range(LE.fruit.diffs)


  LE.nuts<-
EX_RISK(qx=mx2qx(qx),n.ageint=n.ageint,r=0,age=age,qual=NULL,interval=NULL,haz=h
az("nuts",paste0("X",1*nuts))/haz("nuts",paste0("X",1*defaults["nuts"])), vdelay = vdelay)
  LE.nuts2<-
EX_RISK(qx=mx2qx(qx),n.ageint=n.ageint,r=0,age=age,qual=NULL,interval=NULL,haz=h
az("nuts",paste0("X",1*nuts2))/haz("nuts",paste0("X",1*defaults["nuts"])), vdelay = vdelay)
```

```
    LE.nuts.diff<-LE.nuts2[age]-LE.nuts[age]

    haz.nuts.mc<-mc(n = n.mc, x = "nuts", y = paste0("X",1*nuts))
    haz.nuts.mc2<-mc(n = n.mc, x = "nuts", y = paste0("X",1*nuts2))
    LE.nuts.diffs<-NULL
    for(i in 1:n.mc) LE.nuts.diffs<-append(LE.nuts.diffs,

EX_RISK(qx=mx2qx(qx),n.ageint=n.ageint,r=0,age=age,qual=NULL,interval=NULL,haz=h
az.nuts.mc2[i]/haz("nuts",paste0("X",1*defaults["nuts"])), vdelay = vdelay)[age]
                                  -
EX_RISK(qx=mx2qx(qx),n.ageint=n.ageint,r=0,age=age,qual=NULL,interval=NULL,haz=h
az.nuts.mc[i]/haz("nuts",paste0("X",1*defaults["nuts"])), vdelay = vdelay)[age])
    LE.ci.nuts <- quantile(x = LE.nuts.diffs, probs = c(.025, .975))
    # LE.ci.nuts <- range(LE.nuts.diffs)


    LE.legumes<-
EX_RISK(qx=mx2qx(qx),n.ageint=n.ageint,r=0,age=age,qual=NULL,interval=NULL,haz=h
az("legumes",paste0("X",1*legumes))/haz("legumes",paste0("X",1*defaults["legumes"])),
vdelay = vdelay)
    LE.legumes2<-
EX_RISK(qx=mx2qx(qx),n.ageint=n.ageint,r=0,age=age,qual=NULL,interval=NULL,haz=h
az("legumes",paste0("X",1*legumes2))/haz("legumes",paste0("X",1*defaults["legumes"])),
vdelay = vdelay)
    LE.legumes.diff<-LE.legumes2[age]-LE.legumes[age]

    haz.legumes.mc<-mc(n = n.mc, x = "legumes", y = paste0("X",1*legumes))
    haz.legumes.mc2<-mc(n = n.mc, x = "legumes", y = paste0("X",1*legumes2))
    LE.legumes.diffs<-NULL
    for(i in 1:n.mc) LE.legumes.diffs<-append(LE.legumes.diffs,

EX_RISK(qx=mx2qx(qx),n.ageint=n.ageint,r=0,age=age,qual=NULL,interval=NULL,haz=h
az.legumes.mc2[i]/haz("legumes",paste0("X",1*defaults["legumes"])), vdelay = vdelay)[age]
                                    -
EX_RISK(qx=mx2qx(qx),n.ageint=n.ageint,r=0,age=age,qual=NULL,interval=NULL,haz=h
az.legumes.mc[i]/haz("legumes",paste0("X",1*defaults["legumes"])), vdelay = vdelay)[age])
    LE.ci.legumes <- quantile(x = LE.legumes.diffs, probs = c(.025, .975))
    # LE.ci.legumes <- range(LE.legumes.diffs)


    LE.fish<-
EX_RISK(qx=mx2qx(qx),n.ageint=n.ageint,r=0,age=age,qual=NULL,interval=NULL,haz=h
az("fish",paste0("X",1*fish))/haz("fish",paste0("X",1*defaults["fish"])), vdelay = vdelay)
    LE.fish2<-
EX_RISK(qx=mx2qx(qx),n.ageint=n.ageint,r=0,age=age,qual=NULL,interval=NULL,haz=h
az("fish",paste0("X",1*fish2))/haz("fish",paste0("X",1*defaults["fish"])), vdelay = vdelay)
    LE.fish.diff<-LE.fish2[age]-LE.fish[age]

    haz.fish.mc<-mc(n = n.mc, x = "fish", y = paste0("X",1*fish))
    haz.fish.mc2<-mc(n = n.mc, x = "fish", y = paste0("X",1*fish2))
```

```
    LE.fish.diffs<-NULL
    for(i in 1:n.mc) LE.fish.diffs<-append(LE.fish.diffs,

EX_RISK(qx=mx2qx(qx),n.ageint=n.ageint,r=0,age=age,qual=NULL,interval=NULL,haz=h
az.fish.mc2[i]/haz("fish",paste0("X",1*defaults["fish"])), vdelay = vdelay)[age]
                                  -
EX_RISK(qx=mx2qx(qx),n.ageint=n.ageint,r=0,age=age,qual=NULL,interval=NULL,haz=h
az.fish.mc[i]/haz("fish",paste0("X",1*defaults["fish"])), vdelay = vdelay)[age])
    LE.ci.fish <- quantile(x = LE.fish.diffs, probs = c(.025, .975))
    # LE.ci.fish <- range(LE.fish.diffs)


    LE.egg<-
EX_RISK(qx=mx2qx(qx),n.ageint=n.ageint,r=0,age=age,qual=NULL,interval=NULL,haz=h
az("egg",paste0("X",1*egg))/haz("egg",paste0("X",1*defaults["egg"])), vdelay = vdelay)
    LE.egg2<-
EX_RISK(qx=mx2qx(qx),n.ageint=n.ageint,r=0,age=age,qual=NULL,interval=NULL,haz=h
az("egg",paste0("X",1*egg2))/haz("egg",paste0("X",1*defaults["egg"])), vdelay = vdelay)
    LE.egg.diff<-LE.egg2[age]-LE.egg[age]

    haz.egg.mc<-mc(n = n.mc, x = "egg", y = paste0("X",1*egg))
    haz.egg.mc2<-mc(n = n.mc, x = "egg", y = paste0("X",1*egg2))
    LE.egg.diffs<-NULL
    for(i in 1:n.mc) LE.egg.diffs<-append(LE.egg.diffs,

EX_RISK(qx=mx2qx(qx),n.ageint=n.ageint,r=0,age=age,qual=NULL,interval=NULL,haz=h
az.egg.mc2[i]/haz("egg",paste0("X",1*defaults["egg"])), vdelay = vdelay)[age]
                                  -
EX_RISK(qx=mx2qx(qx),n.ageint=n.ageint,r=0,age=age,qual=NULL,interval=NULL,haz=h
az.egg.mc[i]/haz("egg",paste0("X",1*defaults["egg"])), vdelay = vdelay)[age])
    LE.ci.egg <- quantile(x = LE.egg.diffs, probs = c(.025, .975))
    # LE.ci.egg <- range(LE.egg.diffs)


    LE.milk<-
EX_RISK(qx=mx2qx(qx),n.ageint=n.ageint,r=0,age=age,qual=NULL,interval=NULL,haz=h
az("milk",paste0("X",1*milk))/haz("milk",paste0("X",1*defaults["milk"])), vdelay = vdelay)
    LE.milk2<-
EX_RISK(qx=mx2qx(qx),n.ageint=n.ageint,r=0,age=age,qual=NULL,interval=NULL,haz=h
az("milk",paste0("X",1*milk2))/haz("milk",paste0("X",1*defaults["milk"])), vdelay = vdelay)
    LE.milk.diff<-LE.milk2[age]-LE.milk[age]

    haz.milk.mc<-mc(n = n.mc, x = "milk", y = paste0("X",1*milk))
    haz.milk.mc2<-mc(n = n.mc, x = "milk", y = paste0("X",1*milk2))
    LE.milk.diffs<-NULL
    for(i in 1:n.mc) LE.milk.diffs<-append(LE.milk.diffs,

EX_RISK(qx=mx2qx(qx),n.ageint=n.ageint,r=0,age=age,qual=NULL,interval=NULL,haz=h
az.milk.mc2[i]/haz("milk",paste0("X",1*defaults["milk"])), vdelay = vdelay)[age]
```

```
                                        -
EX_RISK(qx=mx2qx(qx),n.ageint=n.ageint,r=0,age=age,qual=NULL,interval=NULL,haz=h
az.milk.mc[i]/haz("milk",paste0("X",1*defaults["milk"])), vdelay = vdelay)[age])
   LE.ci.milk <- quantile(x = LE.milk.diffs, probs = c(.025, .975))
   # LE.ci.milk <- range(LE.milk.diffs)


   LE.refinedgrains<-
EX_RISK(qx=mx2qx(qx),n.ageint=n.ageint,r=0,age=age,qual=NULL,interval=NULL,haz=h
az("refinedgrains",paste0("X",1*refinedgrains))/haz("refinedgrains",paste0("X",1*defaults["r
efinedgrains"])), vdelay = vdelay)
   LE.refinedgrains2<-
EX_RISK(qx=mx2qx(qx),n.ageint=n.ageint,r=0,age=age,qual=NULL,interval=NULL,haz=h
az("refinedgrains",paste0("X",1*refinedgrains2))/haz("refinedgrains",paste0("X",1*defaults["
refinedgrains"])), vdelay = vdelay)
   LE.refinedgrains.diff<-LE.refinedgrains2[age]-LE.refinedgrains[age]

   haz.refinedgrains.mc<-mc(n = n.mc, x = "refinedgrains", y = paste0("X",1*refinedgrains))
   haz.refinedgrains.mc2<-mc(n = n.mc, x = "refinedgrains", y =
paste0("X",1*refinedgrains2))
   LE.refinedgrains.diffs<-NULL
   for(i in 1:n.mc) LE.refinedgrains.diffs<-append(LE.refinedgrains.diffs,

EX_RISK(qx=mx2qx(qx),n.ageint=n.ageint,r=0,age=age,qual=NULL,interval=NULL,haz=h
az.refinedgrains.mc2[i]/haz("refinedgrains",paste0("X",1*defaults["refinedgrains"])), vdelay
= vdelay)[age]
                                              -
EX_RISK(qx=mx2qx(qx),n.ageint=n.ageint,r=0,age=age,qual=NULL,interval=NULL,haz=h
az.refinedgrains.mc[i]/haz("refinedgrains",paste0("X",1*defaults["refinedgrains"])), vdelay =
vdelay)[age])
   LE.ci.refinedgrains <- quantile(x = LE.refinedgrains.diffs, probs = c(.025, .975))
   # LE.ci.refinedgrains <- range(x = LE.refinedgrains.diffs, probs = c(.025, .975))


   LE.meat_red<-
EX_RISK(qx=mx2qx(qx),n.ageint=n.ageint,r=0,age=age,qual=NULL,interval=NULL,haz=h
az("meat_red",paste0("X",1*meat_red))/haz("meat_red",paste0("X",1*defaults["meat_red"]))
, vdelay = vdelay)
   LE.meat_red2<-
EX_RISK(qx=mx2qx(qx),n.ageint=n.ageint,r=0,age=age,qual=NULL,interval=NULL,haz=h
az("meat_red",paste0("X",1*meat_red2))/haz("meat_red",paste0("X",1*defaults["meat_red"])
), vdelay = vdelay)
   LE.meat_red.diff<-LE.meat_red2[age]-LE.meat_red[age]

   haz.meat_red.mc<-mc(n = n.mc, x = "meat_red", y = paste0("X",1*meat_red))
   haz.meat_red.mc2<-mc(n = n.mc, x = "meat_red", y = paste0("X",1*meat_red2))
   LE.meat_red.diffs<-NULL
   for(i in 1:n.mc) LE.meat_red.diffs<-append(LE.meat_red.diffs,

EX_RISK(qx=mx2qx(qx),n.ageint=n.ageint,r=0,age=age,qual=NULL,interval=NULL,haz=h
```

```
az.meat_red.mc2[i]/haz("meat_red",paste0("X",1*defaults["meat_red"])), vdelay =
vdelay)[age]
                                    -
EX_RISK(qx=mx2qx(qx),n.ageint=n.ageint,r=0,age=age,qual=NULL,interval=NULL,haz=h
az.meat_red.mc[i]/haz("meat_red",paste0("X",1*defaults["meat_red"])), vdelay =
vdelay)[age])
   LE.ci.meat_red <- quantile(x = LE.meat_red.diffs, probs = c(.025, .975))
   # LE.ci.meat_red <- range(LE.meat_red.diffs)


   LE.meat_processed<-
EX_RISK(qx=mx2qx(qx),n.ageint=n.ageint,r=0,age=age,qual=NULL,interval=NULL,haz=h
az("meat_processed",paste0("X",1*meat_processed))/haz("meat_processed",paste0("X",1*de
faults["meat_processed"])), vdelay = vdelay)
   LE.meat_processed2<-
EX_RISK(qx=mx2qx(qx),n.ageint=n.ageint,r=0,age=age,qual=NULL,interval=NULL,haz=h
az("meat_processed",paste0("X",1*meat_processed2))/haz("meat_processed",paste0("X",1*d
efaults["meat_processed"])), vdelay = vdelay)
   LE.meat_processed.diff<-LE.meat_processed2[age]-LE.meat_processed[age]

   haz.meat_processed.mc<-mc(n = n.mc, x = "meat_processed", y =
paste0("X",1*meat_processed))
   haz.meat_processed.mc2<-mc(n = n.mc, x = "meat_processed", y =
paste0("X",1*meat_processed2))
   LE.meat_processed.diffs<-NULL
   for(i in 1:n.mc) LE.meat_processed.diffs<-append(LE.meat_processed.diffs,

EX_RISK(qx=mx2qx(qx),n.ageint=n.ageint,r=0,age=age,qual=NULL,interval=NULL,haz=h
az.meat_processed.mc2[i]/haz("meat_processed",paste0("X",1*defaults["meat_processed"])),
vdelay = vdelay)[age]
                                         -
EX_RISK(qx=mx2qx(qx),n.ageint=n.ageint,r=0,age=age,qual=NULL,interval=NULL,haz=h
az.meat_processed.mc[i]/haz("meat_processed",paste0("X",1*defaults["meat_processed"])),
vdelay = vdelay)[age])
   LE.ci.meat_processed <- quantile(x = LE.meat_processed.diffs, probs = c(.025, .975))
   # LE.ci.meat_processed <- range(LE.meat_processed.diffs)


   LE.meat_white<-
EX_RISK(qx=mx2qx(qx),n.ageint=n.ageint,r=0,age=age,qual=NULL,interval=NULL,haz=h
az("meat_white",paste0("X",1*meat_white))/haz("meat_white",paste0("X",1*defaults["meat_
white"])), vdelay = vdelay)
   LE.meat_white2<-
EX_RISK(qx=mx2qx(qx),n.ageint=n.ageint,r=0,age=age,qual=NULL,interval=NULL,haz=h
az("meat_white",paste0("X",1*meat_white2))/haz("meat_white",paste0("X",1*defaults["meat
_white"])), vdelay = vdelay)
   LE.meat_white.diff<-LE.meat_white2[age]-LE.meat_white[age]

   haz.meat_white.mc<-mc(n = n.mc, x = "meat_white", y = paste0("X",1*meat_white))
   haz.meat_white.mc2<-mc(n = n.mc, x = "meat_white", y = paste0("X",1*meat_white2))
```

```
    LE.meat_white.diffs<-NULL
    for(i in 1:n.mc) LE.meat_white.diffs<-append(LE.meat_white.diffs,

EX_RISK(qx=mx2qx(qx),n.ageint=n.ageint,r=0,age=age,qual=NULL,interval=NULL,haz=h
az.meat_white.mc2[i]/haz("meat_white",paste0("X",1*defaults["meat_white"])), vdelay =
vdelay)[age]
                                   -
EX_RISK(qx=mx2qx(qx),n.ageint=n.ageint,r=0,age=age,qual=NULL,interval=NULL,haz=h
az.meat_white.mc[i]/haz("meat_white",paste0("X",1*defaults["meat_white"])), vdelay =
vdelay)[age])
    LE.ci.meat_white <- quantile(x = LE.meat_white.diffs, probs = c(.025, .975))
    # LE.ci.meat_white <- range(LE.meat_white.diffs)


    LE.ssb<-
EX_RISK(qx=mx2qx(qx),n.ageint=n.ageint,r=0,age=age,qual=NULL,interval=NULL,haz=h
az("ssb",paste0("X",1*ssb))/haz("ssb",paste0("X",1*defaults["ssb"])), vdelay = vdelay)
    LE.ssb2<-
EX_RISK(qx=mx2qx(qx),n.ageint=n.ageint,r=0,age=age,qual=NULL,interval=NULL,haz=h
az("ssb",paste0("X",1*ssb2))/haz("ssb",paste0("X",1*defaults["ssb"])), vdelay = vdelay)
    LE.ssb.diff<-LE.ssb2[age]-LE.ssb[age]

    haz.ssb.mc<-mc(n = n.mc, x = "ssb", y = paste0("X",1*ssb))
    haz.ssb.mc2<-mc(n = n.mc, x = "ssb", y = paste0("X",1*ssb2))
    LE.ssb.diffs<-NULL
    for(i in 1:n.mc) LE.ssb.diffs<-append(LE.ssb.diffs,

EX_RISK(qx=mx2qx(qx),n.ageint=n.ageint,r=0,age=age,qual=NULL,interval=NULL,haz=h
az.ssb.mc2[i]/haz("ssb",paste0("X",1*defaults["ssb"])), vdelay = vdelay)[age]
                              -
EX_RISK(qx=mx2qx(qx),n.ageint=n.ageint,r=0,age=age,qual=NULL,interval=NULL,haz=h
az.ssb.mc[i]/haz("ssb",paste0("X",1*defaults["ssb"])), vdelay = vdelay)[age])
    LE.ci.ssb <- quantile(x = LE.ssb.diffs, probs = c(.025, .975))
    # LE.ci.ssb <- range(LE.ssb.diffs)


    # LE.oil<-
EX_RISK(qx=mx2qx(qx),n.ageint=n.ageint,r=0,age=age,qual=NULL,interval=NULL,haz=h
az("oil",paste0("X",1*oil))/haz("oil",paste0("X",1*defaults["oil"])), vdelay = vdelay)
    # LE.oil2<-
EX_RISK(qx=mx2qx(qx),n.ageint=n.ageint,r=0,age=age,qual=NULL,interval=NULL,haz=h
az("oil",paste0("X",1*oil2))/haz("oil",paste0("X",1*defaults["oil"])), vdelay = vdelay)
    # LE.oil.diff<-LE.oil2[age]-LE.oil[age]

    # haz.oil.mc<-mc(n = n.mc, x = "oil", y = paste0("X",1*oil))
    # haz.oil.mc2<-mc(n = n.mc, x = "oil", y = paste0("X",1*oil2))
    # LE.oil.diffs<-NULL
    # for(i in 1:n.mc) LE.oil.diffs<-append(LE.oil.diffs,
```

```
   #
EX_RISK(qx=mx2qx(qx),n.ageint=n.ageint,r=0,age=age,qual=NULL,interval=NULL,haz=h
az.oil.mc2[i]/haz("oil",paste0("X",1*defaults["oil"])), vdelay = vdelay)[age]
   #                              -
EX_RISK(qx=mx2qx(qx),n.ageint=n.ageint,r=0,age=age,qual=NULL,interval=NULL,haz=h
az.oil.mc[i]/haz("oil",paste0("X",1*defaults["oil"])), vdelay = vdelay)[age])
   # LE.ci.oil <- quantile(x = LE.oil.diffs, probs = c(.025, .975))

   LE.ref <-
EX_RISK(qx=mx2qx(qx),n.ageint=n.ageint,r=0,age=1,qual=NULL,interval=NULL,haz=1,vd
elay = vdelay)

   hrs <-
c(LE.wholegrains.diff,LE.veg.diff,LE.fruit.diff,LE.nuts.diff,LE.legumes.diff,LE.fish.diff,LE.e
gg.diff,LE.milk.diff,LE.refinedgrains.diff,LE.meat_red.diff,LE.meat_processed.diff,LE.meat_
white.diff,LE.ssb.diff) #,LE.oil.diff)
   cils <-
c(LE.ci.wholegrains[1],LE.ci.veg[1],LE.ci.fruit[1],LE.ci.nuts[1],LE.ci.legumes[1],LE.ci.fish[
1],LE.ci.egg[1],LE.ci.milk[1],LE.ci.refinedgrains[1],LE.ci.meat_red[1],LE.ci.meat_processed
[1],LE.ci.meat_white[1],LE.ci.ssb[1]) #,LE.ci.oil[1])
   cihs <-
c(LE.ci.wholegrains[2],LE.ci.veg[2],LE.ci.fruit[2],LE.ci.nuts[2],LE.ci.legumes[2],LE.ci.fish[
2],LE.ci.egg[2],LE.ci.milk[2],LE.ci.refinedgrains[2],LE.ci.meat_red[2],LE.ci.meat_processed
[2],LE.ci.meat_white[2],LE.ci.ssb[2]) #,LE.ci.oil[2])

   ####### Total effect

   ## Aim: Same UI for food UI and total UI if only one group is changed
   haz.tot.mc <- rep(1,n.mc)
   haz.tot.mc2 <- rep(1,n.mc)
   LE.diffs <-
c(LE.wholegrains.diff,LE.veg.diff,LE.fruit.diff,LE.nuts.diff,LE.legumes.diff,LE.fish.diff,LE.e
gg.diff,LE.milk.diff,LE.refinedgrains.diff,LE.meat_red.diff,LE.meat_processed.diff,LE.meat_
white.diff,LE.ssb.diff);round(LE.diffs,2)
   #    LE.diffs <-
c(LE.wholegrains.diff,LE.veg.diff,LE.fruit.diff,LE.nuts.diff,LE.legumes.diff,LE.fish.diff,LE.e
gg.diff,LE.milk.diff,LE.refinedgrains.diff,LE.meat_red.diff,LE.meat_processed.diff,LE.meat_
white.diff,LE.ssb.diff,LE.oil.diff);round(LE.diffs,2)
   #print(LE.diffs)
   #print(sum(LE.diffs!=0))
   if(sum(LE.diffs!=0)>1){
     ## Before change
     mc.hazs <-
cbind(haz.wholegrains.mc,haz.veg.mc,haz.fruit.mc,haz.nuts.mc,haz.legumes.mc,haz.fish.mc,h
az.egg.mc,haz.milk.mc,haz.refinedgrains.mc,haz.meat_red.mc,haz.meat_processed.mc,haz.m
eat_white.mc,haz.ssb.mc);head(round(mc.hazs,2),5)
     #      mc.hazs <-
cbind(haz.wholegrains.mc,haz.veg.mc,haz.fruit.mc,haz.nuts.mc,haz.legumes.mc,haz.fish.mc,h
az.egg.mc,haz.milk.mc,haz.refinedgrains.mc,haz.meat_red.mc,haz.meat_processed.mc,haz.m
eat_white.mc,haz.ssb.mc,haz.oil.mc);head(round(mc.hazs,2),5)
```

```
      mc.hazs <- mc.hazs[,which(LE.diffs!=0)];head(round(mc.hazs,2),5)
      haz.tot.mc <- apply(mc.hazs,1,prod);round(haz.tot.mc,2)
      ## After change
      mc.hazs2 <-
cbind(haz.wholegrains.mc2,haz.veg.mc2,haz.fruit.mc2,haz.nuts.mc2,haz.legumes.mc2,haz.fis
h.mc2,haz.egg.mc2,haz.milk.mc2,haz.refinedgrains.mc2,haz.meat_red.mc2,haz.meat_process
ed.mc2,haz.meat_white.mc2,haz.ssb.mc2);head(round(mc.hazs,2),5)
      # mc.hazs2 <-
cbind(haz.wholegrains.mc2,haz.veg.mc2,haz.fruit.mc2,haz.nuts.mc2,haz.legumes.mc2,haz.fis
h.mc2,haz.egg.mc2,haz.milk.mc2,haz.refinedgrains.mc2,haz.meat_red.mc2,haz.meat_process
ed.mc2,haz.meat_white.mc2,haz.ssb.mc2,haz.oil.mc2);head(round(mc.hazs,2),5)
      mc.hazs2 <- mc.hazs2[,which(LE.diffs!=0)];head(round(mc.hazs2,2),5)
      haz.tot.mc2 <- apply(mc.hazs2,1,prod);round(haz.tot.mc2,2)
    }
    if(sum(LE.diffs!=0)==1){
      ## Before change
      mc.hazs <-
cbind(haz.wholegrains.mc,haz.veg.mc,haz.fruit.mc,haz.nuts.mc,haz.legumes.mc,haz.fish.mc,h
az.egg.mc,haz.milk.mc,haz.refinedgrains.mc,haz.meat_red.mc,haz.meat_processed.mc,haz.m
eat_white.mc,haz.ssb.mc);head(round(mc.hazs,2),5)
      #      mc.hazs <-
cbind(haz.wholegrains.mc,haz.veg.mc,haz.fruit.mc,haz.nuts.mc,haz.legumes.mc,haz.fish.mc,h
az.egg.mc,haz.milk.mc,haz.refinedgrains.mc,haz.meat_red.mc,haz.meat_processed.mc,haz.m
eat_white.mc,haz.ssb.mc,haz.oil.mc);head(round(mc.hazs,2),5)
      mc.hazs <- mc.hazs[,which(LE.diffs!=0)];head(round(mc.hazs,2),5)
      haz.tot.mc <- mc.hazs
      ## After change
      mc.hazs2 <-
cbind(haz.wholegrains.mc2,haz.veg.mc2,haz.fruit.mc2,haz.nuts.mc2,haz.legumes.mc2,haz.fis
h.mc2,haz.egg.mc2,haz.milk.mc2,haz.refinedgrains.mc2,haz.meat_red.mc2,haz.meat_process
ed.mc2,haz.meat_white.mc2,haz.ssb.mc2);head(round(mc.hazs,2),5)
      #      mc.hazs2 <-
cbind(haz.wholegrains.mc2,haz.veg.mc2,haz.fruit.mc2,haz.nuts.mc2,haz.legumes.mc2,haz.fis
h.mc2,haz.egg.mc2,haz.milk.mc2,haz.refinedgrains.mc2,haz.meat_red.mc2,haz.meat_process
ed.mc2,haz.meat_white.mc2,haz.ssb.mc2,haz.oil.mc2);head(round(mc.hazs,2),5)
      mc.hazs2 <- mc.hazs2[,which(LE.diffs!=0)];head(round(mc.hazs2,2),5)
      haz.tot.mc2 <- mc.hazs2
    }
    #print(haz.tot.mc)
    #print(haz.tot.mc2)
    # haz.tot.mc <-
haz.wholegrains.mc*haz.veg.mc*haz.fruit.mc*haz.nuts.mc*haz.legumes.mc*haz.fish.mc*haz
.egg.mc*haz.milk.mc*haz.refinedgrains.mc*haz.meat_red.mc*haz.meat_processed.mc*haz.m
eat_white.mc*haz.ssb.mc*haz.oil.mc;round(haz.tot.mc,2)
    # haz.tot.mc2 <-
haz.wholegrains.mc2*haz.veg.mc2*haz.fruit.mc2*haz.nuts.mc2*haz.legumes.mc2*haz.fish.m
c2*haz.egg.mc2*haz.milk.mc2*haz.refinedgrains.mc2*haz.meat_red.mc2*haz.meat_processe
d.mc2*haz.meat_white.mc2*haz.ssb.mc2*haz.oil.mc2;round(haz.tot.mc2,2)

    LE.tot.diffs<-NULL
```

```
    for(i in 1:n.mc) LE.tot.diffs<-append(LE.tot.diffs,

EX_RISK(qx=mx2qx(qx),n.ageint=n.ageint,r=0,age=age,qual=NULL,interval=NULL,haz=h
az.tot.mc2[i], vdelay = vdelay)[age]
                                    -
EX_RISK(qx=mx2qx(qx),n.ageint=n.ageint,r=0,age=age,qual=NULL,interval=NULL,haz=h
az.tot.mc[i], vdelay = vdelay)[age])
    LE.ci.tot <- quantile(x = LE.tot.diffs, probs = c(.025, .975))
    # LE.ci.tot <- range(LE.tot.diffs)
    ####### End total effect #######

    # hazratio <- haz.tot2/ haz.tot

    #
list(LE=LE,LE2=LE2,LE.diff=LE.diff,LE.ref=LE.ref,age=age,energy=energy,energy2=energ
y2,
    list(LE=LE,LE2=LE2,LE.diff=LE.diff,age=age,
         hrs=hrs,cils=cils,cihs=cihs,
         LE.ref=LE.ref,

LE.wholegrains.diff=LE.wholegrains.diff,LE.veg.diff=LE.veg.diff,LE.fruit.diff=LE.fruit.diff,
LE.nuts.diff=LE.nuts.diff,LE.legumes.diff=LE.legumes.diff,LE.fish.diff=LE.fish.diff,LE.egg.
diff=LE.egg.diff,LE.milk.diff=LE.milk.diff,LE.refinedgrains.diff=LE.refinedgrains.diff,LE.m
eat_red.diff=LE.meat_red.diff,LE.meat_processed.diff=LE.meat_processed.diff,LE.meat_whi
te.diff=LE.meat_white.diff,LE.ssb.diff=LE.ssb.diff,

LE.ci.wholegrains=LE.ci.wholegrains,LE.ci.veg=LE.ci.veg,LE.ci.fruit=LE.ci.fruit,LE.ci.nuts
=LE.ci.nuts,LE.ci.legumes=LE.ci.legumes,LE.ci.fish=LE.ci.fish,LE.ci.egg=LE.ci.egg,LE.ci.
milk=LE.ci.milk,LE.ci.refinedgrains=LE.ci.refinedgrains,LE.ci.meat_red=LE.ci.meat_red,LE
.ci.meat_processed=LE.ci.meat_processed,LE.ci.meat_white=LE.ci.meat_white,LE.ci.ssb=L
E.ci.ssb,

#LE.wholegrains.diff=LE.wholegrains.diff,LE.veg.diff=LE.veg.diff,LE.fruit.diff=LE.fruit.dif
f,LE.nuts.diff=LE.nuts.diff,LE.legumes.diff=LE.legumes.diff,LE.fish.diff=LE.fish.diff,LE.eg
g.diff=LE.egg.diff,LE.milk.diff=LE.milk.diff,LE.refinedgrains.diff=LE.refinedgrains.diff,LE.
meat_red.diff=LE.meat_red.diff,LE.meat_processed.diff=LE.meat_processed.diff,LE.meat_w
hite.diff=LE.meat_white.diff,LE.ssb.diff=LE.ssb.diff,LE.oil.diff=LE.oil.diff,

#LE.ci.wholegrains=LE.ci.wholegrains,LE.ci.veg=LE.ci.veg,LE.ci.fruit=LE.ci.fruit,LE.ci.nut
s=LE.ci.nuts,LE.ci.legumes=LE.ci.legumes,LE.ci.fish=LE.ci.fish,LE.ci.egg=LE.ci.egg,LE.ci.
milk=LE.ci.milk,LE.ci.refinedgrains=LE.ci.refinedgrains,LE.ci.meat_red=LE.ci.meat_red,LE
.ci.meat_processed=LE.ci.meat_processed,LE.ci.meat_white=LE.ci.meat_white,LE.ci.ssb=L
E.ci.ssb,LE.ci.oil=LE.ci.oil,
         LE.ci.tot=LE.ci.tot,
         lci.table=lci.table,uci.table=uci.table,
         qx=qx,
         m=m)
  })
  output$LE<-renderText({
    LE<-data()[["LE"]]
```

```
    LE.ref<-data()[["LE.ref"]]
    age<-data()[["age"]];#print(age)
    #energy<-data()[["energy"]]
    #if(energy<4000) paste("Energy intake", energy, "so low, the estimate is uncertain")
    #if(energy>16000) paste("Energy intake", energy, "so high, the estimate is uncertain")
    paste("Expected years left: ",round(LE[age],1),"Total: ",age+round(LE[age],1),"Reference
from birth: ",round(LE.ref[1],1),"Age + reference from age : ",age+round(LE.ref[age],1))
  })
  output$LE2<-renderText({
    LE2<-data()[["LE2"]]
    LE.ref<-data()[["LE.ref"]]
    age<-data()[["age"]];#print(age)
    #energy2<-data()[["energy2"]]
    #if(energy<4000) paste("Energy intake", energy, "so low, the estimate is uncertain")
    #if(energy>25000) paste("Energy intake", energy, "so high, the estimate is uncertain")
    #Uncertain measures on food pattern due to insufficient data on high/low energy levels
    paste("Expected years left: ",round(LE2[age],1),"Total:
",age+round(LE2[age],1),"Reference from birth: ",round(LE.ref[1],1),"Age + reference from
age : ",age+round(LE.ref[age],1))
  })
  output$LE.diff<-renderText({
    LE.diff <- data()[["LE.diff"]]
    LE.ci.tot <- data()[["LE.ci.tot"]]
    age<-data()[["age"]]
    if(!is.na(LE.diff[age])) paste("Years gained/lost for prolonged change",
round(LE.diff[age],1),"(",round(LE.ci.tot[1],1),";",round(LE.ci.tot[2],1),")")
  })

  # output$LE.diff<-renderText({
  #   LE.diff <- data()[["LE.diff"]]
  #   LE.ci.tot <- data()[["LE.ci.tot"]]
  #   age<-data()[["age"]]
  #       if(!is.na(LE.diff[age])) paste("Total HR", round(hazratio,2),"","","")
  # })
  output$LE.wholegrains.diff<-renderText({
    LE.wholegrains.diff<-data()[["LE.wholegrains.diff"]]
    LE.ci.wholegrains<-data()[["LE.ci.wholegrains"]]
    age<-data()[["age"]];#print(age)
    paste("Years gained/lost for prolonged change",round(LE.wholegrains.diff,1),"
(",round(LE.ci.wholegrains[1],1),";", round(LE.ci.wholegrains[2],1),")")
  })

  output$LE.veg.diff<-renderText({
    LE.veg.diff<-data()[["LE.veg.diff"]]
    LE.ci.veg<-data()[["LE.ci.veg"]]
    age<-data()[["age"]];#print(age)
    paste("Years gained/lost for prolonged change",round(LE.veg.diff,1),"
(",round(LE.ci.veg[1],1),";", round(LE.ci.veg[2],1),")")
  })
```

```
  output$LE.fruit.diff<-renderText({
    LE.fruit.diff<-data()[["LE.fruit.diff"]]
    LE.ci.fruit<-data()[["LE.ci.fruit"]]
    age<-data()[["age"]];#print(age)
    paste("Years gained/lost for prolonged change",round(LE.fruit.diff,1),"
(",round(LE.ci.fruit[1],1),";", round(LE.ci.fruit[2],1),")")
  })

  output$LE.nuts.diff<-renderText({
    LE.nuts.diff<-data()[["LE.nuts.diff"]]
    LE.ci.nuts<-data()[["LE.ci.nuts"]]
    age<-data()[["age"]];#print(age)
    paste("Years gained/lost for prolonged change",round(LE.nuts.diff,1),"
(",round(LE.ci.nuts[1],1),";", round(LE.ci.nuts[2],1),")")
  })

  output$LE.legumes.diff<-renderText({
    LE.legumes.diff<-data()[["LE.legumes.diff"]]
    LE.ci.legumes<-data()[["LE.ci.legumes"]]
    age<-data()[["age"]];#print(age)
    paste("Years gained/lost for prolonged change",round(LE.legumes.diff,1),"
(",round(LE.ci.legumes[1],1),";", round(LE.ci.legumes[2],1),")")
  })

  output$LE.fish.diff<-renderText({
    LE.fish.diff<-data()[["LE.fish.diff"]]
    LE.ci.fish<-data()[["LE.ci.fish"]]
    age<-data()[["age"]];#print(age)
    paste("Years gained/lost for prolonged change",round(LE.fish.diff,1),"
(",round(LE.ci.fish[1],1),";", round(LE.ci.fish[2],1),")")
  })

  output$LE.egg.diff<-renderText({
    LE.egg.diff<-data()[["LE.egg.diff"]]
    LE.ci.egg<-data()[["LE.ci.egg"]]
    age<-data()[["age"]];#print(age)
    paste("Years gained/lost for prolonged change",round(LE.egg.diff,1),"
(",round(LE.ci.egg[1],1),";", round(LE.ci.egg[2],1),")")
  })

  output$LE.milk.diff<-renderText({
    LE.milk.diff<-data()[["LE.milk.diff"]]
    LE.ci.milk<-data()[["LE.ci.milk"]]
    age<-data()[["age"]];#print(age)
    paste("Years gained/lost for prolonged change",round(LE.milk.diff,1),"
(",round(LE.ci.milk[1],1),";", round(LE.ci.milk[2],1),")")
  })

  output$LE.refinedgrains.diff<-renderText({
    LE.refinedgrains.diff<-data()[["LE.refinedgrains.diff"]]
```

```
    LE.ci.refinedgrains<-data()[["LE.ci.refinedgrains"]]
    age<-data()[["age"]];#print(age)
    paste("Years gained/lost for prolonged change",round(LE.refinedgrains.diff,1),"
(",round(LE.ci.refinedgrains[1],1),";", round(LE.ci.refinedgrains[2],1),")")
  })

  output$LE.meat_red.diff<-renderText({
    LE.meat_red.diff<-data()[["LE.meat_red.diff"]]
    LE.ci.meat_red<-data()[["LE.ci.meat_red"]]
    age<-data()[["age"]];#print(age)
    paste("Years gained/lost for prolonged change",round(LE.meat_red.diff,1),"
(",round(LE.ci.meat_red[1],1),";", round(LE.ci.meat_red[2],1),")")
  })

  output$LE.meat_processed.diff<-renderText({
    LE.meat_processed.diff<-data()[["LE.meat_processed.diff"]]
    LE.ci.meat_processed<-data()[["LE.ci.meat_processed"]]
    age<-data()[["age"]];#print(age)
    paste("Years gained/lost for prolonged change",round(LE.meat_processed.diff,1),"
(",round(LE.ci.meat_processed[1],1),";", round(LE.ci.meat_processed[2],1),")")
  })

  output$LE.meat_white.diff<-renderText({
    LE.meat_white.diff<-data()[["LE.meat_white.diff"]]
    LE.ci.meat_white<-data()[["LE.ci.meat_white"]]
    age<-data()[["age"]];#print(age)
    paste("Years gained/lost for prolonged change",round(LE.meat_white.diff,1),"
(",round(LE.ci.meat_white[1],1),";", round(LE.ci.meat_white[2],1),")")
  })

  output$LE.ssb.diff<-renderText({
    LE.ssb.diff<-data()[["LE.ssb.diff"]]
    LE.ci.ssb<-data()[["LE.ci.ssb"]]
    age<-data()[["age"]];#print(age)
    paste("Years gained/lost for prolonged change",round(LE.ssb.diff,1),"
(",round(LE.ci.ssb[1],1),";", round(LE.ci.ssb[2],1),")")
  })

  # output$LE.oil.diff<-renderText({
  #   LE.oil.diff<-data()[["LE.oil.diff"]]
  #   LE.ci.oil<-data()[["LE.ci.oil"]]
  #   age<-data()[["age"]];#print(age)
  #   paste("Years gained/lost for prolonged change",round(LE.oil.diff,1),"
(",round(LE.ci.oil[1],1),";", round(LE.ci.oil[2],1),")")
  # })


  # output$nutrigrade<-renderText({
  #energy2<-data()[["energy2"]]
  #LE.wholegrains.diff<-data()[["LE.wholegrains.diff"]]
```

```
  #LE.veg.diff<-data()[["LE.veg.diff"]]
  #LE.fruit.diff<-data()[["LE.fruit.diff"]]
  #LE.nuts.diff<-data()[["LE.nuts.diff"]]
  #LE.legumes.diff<-data()[["LE.legumes.diff"]]
  #LE.fish.diff<-data()[["LE.fish.diff"]]
  #LE.egg.diff<-data()[["LE.egg.diff"]]
  #LE.milk.diff<-data()[["LE.milk.diff"]]
  #LE.refinedgrains.diff<-data()[["LE.refinedgrains.diff"]]
  #LE.meat_red.diff<-data()[["LE.meat_red.diff"]]
  #LE.meat_processed.diff<-data()[["LE.meat_processed.diff"]]
  #LE.meat_white.diff<-data()[["LE.meat_white.diff"]]
  #LE.ssb.diff<-data()[["LE.ssb.diff"]]
  #LE.oil.diff<-data()[["LE.oil.diff"]]
  #ng<-
(abs(LE.wholegrains.diff)*nutrigrade["wholegrains"]+abs(LE.veg.diff)*nutrigrade["veg"]+ab
s(LE.fruit.diff)*nutrigrade["fruit"]+abs(LE.nuts.diff)*nutrigrade["nuts"]+abs(LE.legumes.diff
)*nutrigrade["legumes"]+abs(LE.fish.diff)*nutrigrade["fish"]+abs(LE.egg.diff)*nutrigrade["e
gg"]+abs(LE.milk.diff)*nutrigrade["milk"]+abs(LE.refinedgrains.diff)*nutrigrade["refinedgra
ins"]+abs(LE.meat_red.diff)*nutrigrade["meat_red"]+abs(LE.meat_processed.diff)*nutrigrad
e["meat_processed"]+abs(LE.meat_white.diff)*nutrigrade["meat_white"]+abs(LE.ssb.diff)*n
utrigrade["ssb"]+abs(LE.oil.diff)*nutrigrade["oil"])/(abs(LE.wholegrains.diff)+abs(LE.veg.di
ff)+abs(LE.fruit.diff)+abs(LE.nuts.diff)+abs(LE.legumes.diff)+abs(LE.fish.diff)+abs(LE.egg.
diff)+abs(LE.milk.diff)+abs(LE.refinedgrains.diff)+abs(LE.meat_red.diff)+abs(LE.meat_proc
essed.diff)+abs(LE.meat_white.diff)+abs(LE.ssb.diff)+abs(LE.oil.diff))
  #if(is.nan(ng)) ngscore<-"-" else if(ng>=8) ngscore<-"high" else if(ng<8 & ng>=6)
ngscore<-"moderate" else if(ng<6) ngscore<-"weak" else ngscore<-"error"
  #paste("Overall NutriGrade meta-evidence quality is",if(is.nan(ng)) "-" else round(ng,1),"
(",ngscore,").", "Estimated total energy",round(energy2),"kJ/day
(",round(energy2/4.184),"kcal/day).")
  #})

  output$downloadData <- downloadHandler(
    filename = function() {
      paste("food-", input$country ,"-", Sys.Date(), ".csv", sep="")    },
    content = function(file) {
      df<-NULL
      age<-data()[["age"]]
      qx<-data()[["qx"]]
      LE.wholegrains.diff<-data()[["LE.wholegrains.diff"]]
      LE.ci.wholegrains<-data()[["LE.ci.wholegrains"]]
      df<-
rbind(df,c(round(LE.wholegrains.diff,1),round(LE.ci.wholegrains[1],1),round(LE.ci.wholegra
ins[2],1)))
      LE.veg.diff<-data()[["LE.veg.diff"]]
      LE.ci.veg<-data()[["LE.ci.veg"]]
      df<-rbind(df,c(round(LE.veg.diff,1),round(LE.ci.veg[1],1),round(LE.ci.veg[2],1)))
      LE.fruit.diff<-data()[["LE.fruit.diff"]]
      LE.ci.fruit<-data()[["LE.ci.fruit"]]
      df<-rbind(df,c(round(LE.fruit.diff,1),round(LE.ci.fruit[1],1),round(LE.ci.fruit[2],1)))
      LE.nuts.diff<-data()[["LE.nuts.diff"]]
```

```
LE.ci.nuts<-data()[["LE.ci.nuts"]]
df<-rbind(df,c(round(LE.nuts.diff,1),round(LE.ci.nuts[1],1),round(LE.ci.nuts[2],1)))
LE.legumes.diff<-data()[["LE.legumes.diff"]]
LE.ci.legumes<-data()[["LE.ci.legumes"]]
df<-rbind(df,c(round(LE.legumes.diff,1),round(LE.ci.legumes[1],1),round(LE.ci.legumes[2],1)))
LE.fish.diff<-data()[["LE.fish.diff"]]
LE.ci.fish<-data()[["LE.ci.fish"]]
df<-rbind(df,c(round(LE.fish.diff,1),round(LE.ci.fish[1],1),round(LE.ci.fish[2],1)))
LE.egg.diff<-data()[["LE.egg.diff"]]
LE.ci.egg<-data()[["LE.ci.egg"]]
df<-rbind(df,c(round(LE.egg.diff,1),round(LE.ci.egg[1],1),round(LE.ci.egg[2],1)))
LE.milk.diff<-data()[["LE.milk.diff"]]
LE.ci.milk<-data()[["LE.ci.milk"]]
df<-rbind(df,c(round(LE.milk.diff,1),round(LE.ci.milk[1],1),round(LE.ci.milk[2],1)))
LE.refinedgrains.diff<-data()[["LE.refinedgrains.diff"]]
LE.ci.refinedgrains<-data()[["LE.ci.refinedgrains"]]
df<-rbind(df,c(round(LE.refinedgrains.diff,1),round(LE.ci.refinedgrains[1],1),round(LE.ci.refinedgrains[2],1)))
LE.meat_red.diff<-data()[["LE.meat_red.diff"]]
LE.ci.meat_red<-data()[["LE.ci.meat_red"]]
df<-rbind(df,c(round(LE.meat_red.diff,1),round(LE.ci.meat_red[1],1),round(LE.ci.meat_red[2],1)))
LE.meat_processed.diff<-data()[["LE.meat_processed.diff"]]
LE.ci.meat_processed<-data()[["LE.ci.meat_processed"]]
df<-rbind(df,c(round(LE.meat_processed.diff,1),round(LE.ci.meat_processed[1],1),round(LE.ci.meat_processed[2],1)))
LE.meat_white.diff<-data()[["LE.meat_white.diff"]]
LE.ci.meat_white<-data()[["LE.ci.meat_white"]]
df<-rbind(df,c(round(LE.meat_white.diff,1),round(LE.ci.meat_white[1],1),round(LE.ci.meat_white[2],1)))
LE.ssb.diff<-data()[["LE.ssb.diff"]];#print(LE.ssb.diff)
LE.ci.ssb<-data()[["LE.ci.ssb"]]
df<-rbind(df,c(round(LE.ssb.diff,1),round(LE.ci.ssb[1],1),round(LE.ci.ssb[2],1)))
#LE.oil.diff<-data()[["LE.oil.diff"]]
#LE.ci.oil<-data()[["LE.ci.oil"]]
#df<-rbind(df,c(round(LE.oil.diff,1),round(LE.ci.oil[1],1),round(LE.ci.oil[2],1)))
LE.diff<-data()[["LE.diff"]]
LE.ci.tot <- data()[["LE.ci.tot"]]

df<-rbind(df,c(round(LE.diff[age],1),round(LE.ci.tot[1],1),round(LE.ci.tot[2],1)))
rownames(df)<-c("wholegrains","veg","fruit","nuts","legumes","fish","egg","milk","refinedgrains","meat_red","meat_processed","meat_white","ssb","total")
```

```
      # rownames(df)<-
c("wholegrains","veg","fruit","nuts","legumes","fish","egg","milk","refinedgrains","meat_red
","meat_processed","meat_white","ssb","oil","total")
      colnames(df)<-c("val","lower","upper")
      write.csv2(df, file="out.csv")
      file.copy("out.csv", file)
    }
  )

  observeEvent(input$eatwell.button,
              {

                uk.eatwell<-c(5,5,4,3,3,3, 2,5,1,2,2,3,1)
                #uk.eatwell<-c(5,5,4,3,3,3, 2,5,1,2,2,3,1,3)
                # Eatwell: egg not specified - thus assumed to be 3, milk/dairy amounts not
specified - thus assumed to be 3, same for white meat
                eatwell<-uk.eatwell
                names(eatwell)<-c("wholegrains", "veg", "fruit", "nuts", "legumes", "fish", "egg",
"milk", "refinedgrains", "meat_red", "meat_processed", "meat_white", "ssb")
                # names(eatwell)<-c("wholegrains", "veg", "fruit", "nuts", "legumes", "fish", "egg",
"milk", "refinedgrains", "meat_red", "meat_processed", "meat_white", "ssb", "oil")

                updateSliderInput(session, "wholegrains2", value =
as.numeric(eatwell["wholegrains"]))
                updateSliderInput(session, "veg2", value = as.numeric(eatwell["veg"]))
                updateSliderInput(session, "fruit2", value = as.numeric(eatwell["fruit"]))
                updateSliderInput(session, "nuts2", value = as.numeric(eatwell["nuts"]))
                updateSliderInput(session, "legumes2", value = as.numeric(eatwell["legumes"]))
                updateSliderInput(session, "fish2", value = as.numeric(eatwell["fish"]))
                updateSliderInput(session, "egg2", value = as.numeric(eatwell["egg"]))
                updateSliderInput(session, "milk2", value = as.numeric(eatwell["milk"]))
                updateSliderInput(session, "refinedgrains2", value =
as.numeric(eatwell["refinedgrains"]))
                updateSliderInput(session, "meat_red2", value = as.numeric(eatwell["meat_red"]))
                updateSliderInput(session, "meat_processed2", value =
as.numeric(eatwell["meat_processed"]))
                updateSliderInput(session, "meat_white2", value =
as.numeric(eatwell["meat_white"]))
                updateSliderInput(session, "ssb2", value = as.numeric(eatwell["ssb"]))
                # updateSliderInput(session, "oil2", value = as.numeric(eatwell["oil"]))
              })

  observeEvent(input$optimal.button,
              {
                #uk.optimals<-c(3,4,3,5,5,3,2,5,1,1,1,3,1,3)
                #making robust optimals
                uk.optimals<-c(3,4,3,2,2,3,2,5,1,1,1,3,2)
                # uk.optimals<-c(3,4,3,2,2,3,2,5,1,1,1,3,2,3)
                optimals<-uk.optimals
```

```
              names(optimals)<-c("wholegrains", "veg", "fruit", "nuts", "legumes", "fish", "egg",
"milk", "refinedgrains", "meat_red", "meat_processed", "meat_white", "ssb")
              # names(optimals)<-c("wholegrains", "veg", "fruit", "nuts", "legumes", "fish",
"egg", "milk", "refinedgrains", "meat_red", "meat_processed", "meat_white", "ssb", "oil")

              updateSliderInput(session, "wholegrains2", value =
as.numeric(optimals["wholegrains"]))
              updateSliderInput(session, "veg2", value = as.numeric(optimals["veg"]))
              updateSliderInput(session, "fruit2", value = as.numeric(optimals["fruit"]))
              updateSliderInput(session, "nuts2", value = as.numeric(optimals["nuts"]))
              updateSliderInput(session, "legumes2", value = as.numeric(optimals["legumes"]))
              updateSliderInput(session, "fish2", value = as.numeric(optimals["fish"]))
              updateSliderInput(session, "egg2", value = as.numeric(optimals["egg"]))
              updateSliderInput(session, "milk2", value = as.numeric(optimals["milk"]))
              updateSliderInput(session, "refinedgrains2", value =
as.numeric(optimals["refinedgrains"]))
              updateSliderInput(session, "meat_red2", value =
as.numeric(optimals["meat_red"]))
              updateSliderInput(session, "meat_processed2", value =
as.numeric(optimals["meat_processed"]))
              updateSliderInput(session, "meat_white2", value =
as.numeric(optimals["meat_white"]))
              updateSliderInput(session, "ssb2", value = as.numeric(optimals["ssb"]))
              # updateSliderInput(session, "oil2", value = as.numeric(optimals["oil"]))
             })

  observeEvent(input$unhealthy.button,
             {
              uk.unhealthy<-c(1,1,1,1,1,1,5,1,5,5,5,1,5)
              # uk.unhealthy<-c(1,1,1,1,1,1,5,1,5,5,5,1,5,3)
              unhealthy<-uk.unhealthy
              names(unhealthy)<-c("wholegrains", "veg", "fruit", "nuts", "legumes", "fish",
"egg", "milk", "refinedgrains", "meat_red", "meat_processed", "meat_white", "ssb")
              # names(unhealthy)<-c("wholegrains", "veg", "fruit", "nuts", "legumes", "fish",
"egg", "milk", "refinedgrains", "meat_red", "meat_processed", "meat_white", "ssb", "oil")

              updateSliderInput(session, "wholegrains", value =
as.numeric(unhealthy["wholegrains"]))
              updateSliderInput(session, "veg", value = as.numeric(unhealthy["veg"]))
              updateSliderInput(session, "fruit", value = as.numeric(unhealthy["fruit"]))
              updateSliderInput(session, "nuts", value = as.numeric(unhealthy["nuts"]))
              updateSliderInput(session, "legumes", value = as.numeric(unhealthy["legumes"]))
              updateSliderInput(session, "fish", value = as.numeric(unhealthy["fish"]))
              updateSliderInput(session, "egg", value = as.numeric(unhealthy["egg"]))
              updateSliderInput(session, "milk", value = as.numeric(unhealthy["milk"]))
              updateSliderInput(session, "refinedgrains", value =
as.numeric(unhealthy["refinedgrains"]))
              updateSliderInput(session, "meat_red", value =
as.numeric(unhealthy["meat_red"]))
```

```
          updateSliderInput(session, "meat_processed", value =
as.numeric(unhealthy["meat_processed"]))
          updateSliderInput(session, "meat_white", value =
as.numeric(unhealthy["meat_white"]))
          updateSliderInput(session, "ssb", value = as.numeric(unhealthy["ssb"]))
          # updateSliderInput(session, "oil", value = as.numeric(unhealthy["oil"]))
        })


  observeEvent(input$extensivemodel,
        {
          updateSliderInput(session, "m", value = 0.75)
        })

  observeEvent(input$standardmodel,
        {
          updateSliderInput(session, "m", value = 0.5)
        })

  output$forest<-renderHighchart({
    rnames<-c("Wholegrains", "Vegetables", "Fruit", "Nuts", "Legumes", "Fish", "Egg",
"Milk/dairy", "Refined grains", "Red meat", "Processed meat", "White meat", "Sugar-
sweetened beverages")
    # rnames<-c("Wholegrains", "Vegetables", "Fruit", "Nuts", "Legumes", "Fish", "Egg",
"Milk/dairy", "Refined grains", "Red meat", "Processed meat", "White meat", "Sugar-
sweetened beverages", "Added oils")
    hrs<-data()[["hrs"]]
    cils<-data()[["cils"]]
    cihs<-data()[["cihs"]]

    forest.tibble <- tibble(rnames= rnames,
                            hrs=round(hrs,2),
                            cils=round(cils,2),
                            cihs=round(cihs,2),
                            hrs2=round2(hrs,2),
                            cils2=round2(cils,2),
                            cihs2=round2(cihs,2),
                            col = rgb(t(col2rgb("grey")), maxColorValue = 255))

    x <- c("Lower ", "Estim. ", "Upper ")
    y <- sprintf("{point.%s}", c("cils", "hrs", "cihs"))

    tltip <- tooltip_table(x, y)

    hchart(forest.tibble,
           type = "columnrange",
           name = "Uncertainty interval",
           hcaes(x = rnames, low = cils, high = cihs)#,
           # style = list(color = col)
    ) %>%
```

```
    hc_chart(inverted = TRUE) %>%
    hc_xAxis(max = 13, min = 0,
             showLastLabel = FALSE,
             title = FALSE,
             color="black",
             showFirstLabel = TRUE) %>%
    hc_add_series(forest.tibble,
                  type = "scatter",
                  name = "Life years gained",
                  hcaes(x = rnames, y = hrs, group = rnames, color = col),
                  # hcaes(y = hrs, group = rnames, color = col),
                  dataLabels = list(enabled =T,
                                    format ='{point.hrs2}',
                                    verticalAlign = 'middle'),
                  showInLegend = F) %>%
    hc_plotOptions(scatter = list(marker = list(radius = 0))
                   # columnrange = list(name = 'Range'),
                   # columnrange = list(dataLabels = list(enabled = TRUE)),
                   # series = list(name = 'Range')
    ) %>%
    hc_tooltip(
      useHTML = TRUE,
      pointFormat = tltip#,
      # headerFormat = as.character(tags$small("{point.x:%d %B, %Y}"))
    ) %>%
    # hc_tooltip(pointFormat = "Gain: {point.y} years") %>%
    hc_yAxis(
      max = 3.1, min = -1.0,
      title = list(text = "Life years gained"), gridLineWidth = 0.5
      ,plotLines = list(list(label = list(text = ""),color = "#FF0000",width = 1,value = 0))
      # ,plotLines = list( color = "#FF0000", width = 0.1, value = 0)
    ) %>%
    # hc_yAxis(
    #   list(title = list(text = "Life years gained"), opposite = TRUE),
    # ) %>%
    hc_exporting(enabled = TRUE)
  })

  output$radar<-renderHighchart({

    rnames<-c("Wholegrains", "Vegetables", "Fruit", "Nuts", "Legumes", "Fish", "Egg",
"Milk/dairy", "Refined grains", "Red meat", "Processed meat", "White meat", "Sugar-
sweetened beverages")
    # rnames<-c("Wholegrains", "Vegetables", "Fruit", "Nuts", "Legumes", "Fish", "Egg",
"Milk/dairy", "Refined grains", "Red meat", "Processed meat", "White meat", "Sugar-
sweetened beverages", "Added oils")
    hrs<-data()[["hrs"]]
    cils<-data()[["cils"]]
    cihs<-data()[["cihs"]]
```

```
radar.tibble <- tibble(rnames= rnames,
                       Estim.=round(hrs,2),
                       Lower=round(cils,2),
                       Upper=round(cihs,2),
                       hrs2=round2(hrs,2),
                       cils2=round2(cils,2),
                       cihs2=round2(cihs,2),
                       col = rgb(t(col2rgb("grey")), maxColorValue = 255))

cols <- c(rgb(t(col2rgb("black")), maxColorValue = 255),
          rgb(t(col2rgb("grey")), maxColorValue = 255),
          rgb(t(col2rgb("grey")), maxColorValue = 255))

# radar.tibble <- tibble(rnames= rep(rnames,3),
#                        values=c(round(hrs,2),round(cils,2),round(cihs,2)),
#                        values2=c(round2(hrs,2),round2(cils,2),round2(cihs,2)),
#                        group = rep(c("Estim.","Lower","Upper"), each = length(hrs)),
#                        col = rep(c(rgb(t(col2rgb("black")), maxColorValue = 255),
#                                    rgb(t(col2rgb("grey")), maxColorValue = 255),
#                                    rgb(t(col2rgb("grey")), maxColorValue = 255)),
#                                  each = length(hrs)))

x <- c("Lower ", "Estim. ", "Upper ")
y <- sprintf("{point.%s}", c("Lower", "Estim.", "Upper"))

tltip <- tooltip_table(x, y)

hchart(
  radar.tibble,
  type = "line" ,
  name = "Life years gained",
  hcaes(
    x = rnames,
    y = Estim.
  )
) |>
  hc_add_series(
    radar.tibble,
    type = "line",
    name = "Upper limit",
    hcaes(
      x = rnames,
      y = Upper
    )
  ) |>
  hc_add_series(
    radar.tibble,
    type = "line",
    name = "Lower limit",
    hcaes(
```

```
        x = rnames,
        y = Lower
      )
    ) |>
    hc_colors(cols) |>
    hc_chart(
      polar = TRUE
    ) |>
    hc_yAxis(
      labels = list(format = "{value}y"),
      tickInterval = .2,
      title = list(text = ""),
      showFirstLabel = TRUE
    ) |>
    hc_xAxis(
      showLastLabel = TRUE,
      # title = FALSE,
      color="black",
      # showFirstLabel = TRUE
      title = list(text = ""),
      gridLineWidth = 0.5
      # labels = list(format = "{value: %b}")
    ) |>
    # hc_tooltip(
    #   useHTML = TRUE,
    #   pointFormat = tltip#,
    #   # headerFormat = as.character(tags$small("{point.x:%d %B, %Y}"))
    # ) |>
    hc_title(
      text = "Life years gained per food group"
    ) |>
    hc_subtitle(
      text = "With lower and upper uncertainty limits"
    ) |>
    hc_pane(line = list(size = '100%')) |>
    hc_plotOptions(line = list(marker = list(radius = 0))
                # columnrange = list(name = 'Range'),
                # columnrange = list(dataLabels = list(enabled = TRUE)),
                # series = list(name = 'Range')
    ) %>%
    hc_exporting(enabled = TRUE)
  })


  output$Help <- renderUI({
    HTML(paste0(h1("Frequently asked questions (FAQ): "),
            br(),br(),
            p(paste0("How do I use the sliders?"), style = "font-size:16px"),
            p(paste0("The left slider bars represent how much of each food group a specific
person eats before change (e.g. the last year). The defaults are set to a the “typical western
```

diet". The right food panel represents diet after change. You could move both before (left sliders) and after (right sliders) to various intake levels. Clicking the "Optimal" button, the right panel of sliders are adjusted to the diet patterns that are estimated to give best outcomes based on available evidence (UK Biobank). Clicking the "Unhealthy" button, the left panel of sliders are adjusted to the diet patterns that are among the least healthy quintile. In the left food panel indicating diet before change, the defaults are set to the "typical UK diet" (TUK, the mid quintiles). The right food panel represents diet after change. After clicking the "Optimal" (HD) or "Eatwell" button (ED), the right panel of sliders are adjusted to the HD and ED diet patterns. ". "), style = "font-size:16px"),

p(paste0("Do years gained for prolonged change mean that I will live this much longer if adopting specific diet changes? Can this tool tell me how long I will live? "), style = "font-size:16px"),

p(paste0("Obviously, there are many factors at play beyond diet impacting on longevity. There could be treatment options that will change life span and disease spectrums and thus there are substantial uncertainties around these estimates. However, these estimates are built under the assumptions of current disease spectrum for people who are "typical" within each of these regions/countries. The estimates could also be an indicator of how good health is as people who live to the age of for example 90 years generally have better health at the age of 60, than people who die at the age of 70 years. "), style = "font-size:16px"),

br(),br(),

p(paste0("How do I understand the output? "), style = "font-size:16px"),

p(paste0("The plot for food items presents estimated contribution to change in life expectancy / longevity from each food group change. The number to the right (which is presented with the point on the line) shows the mean estimates while the lines present the degree of uncertainty based on the primary data sources (however there could be additional layers of uncertainty on top of this). The total diagram presents total change in life expectancy. "), style = "font-size:16px"),

br(),br(),

p(paste0("What is "model adjustment"? "), style = "font-size:16px"),

p(paste0("Our estimates use data from UK Biobank that are adjusted for factors such as smoking, exercise, age and sex. However, some "residual confounding" may still affect the estimates. This could in principle go either direction, but is more likely to overestimate the effect sizes. There is also a risk of over-adjustment as some of the studies included in meta-analyses adjusted for potential intermediate factors that might contribute to underestimating effect measures. If moving the adjustment parameter to 0.5 the model becomes more conservative in the sense that the effect of dietary changes is reduced by 50% while 1.0 does not make the model more "conservative". The default is 0.5."), style = "font-size:16px"),

br(),br(),

p(paste0("Similarly, we know that effects of a healthy diet is unlikely to have full effect immediately and for conditions such as cardiovascular disease and cancers, the time before reaching "full effects" are likely to be several decades.

We have used 20 years as assumption, but it is possible to check how this changes the estimates when modifying the assumption to different time ranges between 0 and 50 years.

"), style = "font-size:16px"),

br(),br(),

```
            p(paste0("What data sources do this evidence build on? "), style = "font-
size:16px"),
            p(paste0("Data on background mortality for specific countries and regions were
obtained from the freely available GBD cause of death database. We extracted data for the
United Kingdom. Region-specific estimates on total mortality rates in 5-year age groups were
also available from GBD. These were converted to single-year age specific mortality rates in
our model. 1 "), style = "font-size:16px"),
            p(paste0("Using data from UK Biobank, we estimate the impact on mortality of
food groups.
                "), style = "font-size:16px"),
            br(),br(),
            p(paste0("How is this calculated “under the hood”? "), style = "font-size:16px"),
            p(paste0("We used the R package Shiny to create a web application
(https://food4healthylife.org/) that enables the estimation of the effect of a range of dietary
change. The details of this method will be published in an article (more details later) "), style
= "font-size:16px"),
            br(),br(),
            p(paste0("References "), style = "font-size:16px"),
            p(paste0("")), a(href="https://doi.org/10.1371/journal.pmed.1003889", "Fadnes LT,
Økland J-M, Haaland ØA, Johansson KA. Estimating impact of food choices on life
expectancy: A modeling study. PLOS Medicine 2022")
    ))
  })
  output$Citation <- renderUI({
    HTML(paste0(p(paste0("Food for healthy life is based on the following paper:"), style =
"font-size:16px"),
            p(paste0(" "),a(href="https://doi.org/10.1371/journal.pmed.1003889", "Fadnes LT,
Økland J-M, Haaland ØA, Johansson KA. Estimating impact of food choices on life
expectancy: A modeling study. PLOS Medicine 2022", target = "_blank"),
              style = "font-size:16px"
            )))
  })
  #
  # a(href="https://www.uib.no/en/bceps/130756/data-visualizations","FairChoices website",
  #   style = "font-size:12px", target = "_blank"),br(),br(),

}

shinyApp(ui=ui,server=server)
```

Table S1. Details on diet component from the UK Biobank study.

Diet component	Intake goal	Field IDs	Amount per serving	Grams per serving
**FFQ**				
Fruits	250g/day (200-300g)	1309 (pieces fresh fruit/day)<br>1319 (pieces dried fruit/day)	1309 – 1 piece<br>1319 – 5 pieces	100g fresh<br>8g dried
Vegetable	360g/day (290-430g)	1289 (tablespoons cooked vegetables/day)<br>1299 (salad/raw vegetables/day)	3 heaped tablespoons	40g cooked<br>30g row
Whole grains	125g/day (100-150g)	1438, 1448 (wholemeal / wholegrain bread)<br>1458, 1468 (bran / oat / muesli cereal bowls/week)	1438/1448 – 1 slice/day<br>1458/1468 – 1 bowl/day	White bread: 36 g<br>Brown bread: 36 g<br>Wholemeal bread: 36 g<br>Other/do not know/prefer not to answer<br>Bran cereal: 40 g<br>Biscuit cereal: 40 g<br>Oat cereal: 160 g<br>Muesli: 55 g<br>Other (e.g. cornflakes): 30 g
Refined grains	≤2 servings/day	1438, 1448 (white, brown, other bread slices/week)<br>1458, 1468 (biscuit, other cereals/week)	1438/1448 – 1 slice/day<br>1458/1468 – 1 bowl/day	
Fish	≥2 servings/week (40g/day, 20g/day oily)	1329 (oily fish/week)<br>1339 (non-oily fish/week)	Once/week	134g oily<br>100g non-oily
Dairy	2 servings/day	1408 (cheese/week)<br>1418 (milk type)	1408 – 1 piece/day<br>1418 – 1 glass/day if consumption of any type of milk	40g cheese<br>250g milk
Processed meats	≤1 serving/week (2g/day (0-4g)	1349 (processed meat/week or daily)<br>3680 (age when last ate meat)		90g
Red meats	≤2 serving/week (23g/day (18-27g/day)	1369 (beef/week or day)<br>1379 (lamb or mutton/week or day)<br>1389 (pork/week or day)		120g
Poultry	≤2 serving/week	1359 (poultry/week or day)		130g
24-hour recall				
Nuts	21g/day (16-25)	102410 (salted peanuts)	1 handful	40g

		102420 (unsalted peanuts)<br>102430 (salted nuts)<br>102440 (salted nuts)		
Legumes		104010 (beans or lentils)	1 serving	150g
Mixed				
Sugar-sweetened beverages	Don’t drink<br>3g/day (0-5g/day)	6144 (never consumes drinks containing sugar)<br>100170 (Fizzy drinks)	Only 0 servings were possible here.	250g
Eggs	No recommendation available	102940 (whole egg)<br>102950 (omelettes/scrambled)<br>102960 (in sandwiches)<br>102970 (scotch eggs)<br>102980 (other dishes)<br>6144 (egg consumer)	1 piece	53g whole egg<br>120g others
Typical diet		100020 (Typical diet)		

Table S2. All-cause mortality hazard ratios for various food groups with uncertainty limits for the UK based on estimates from UK Biobank.

Analysis	Outcome	Exposure	Range (intal	Total n	Deaths	HR 95%CI
trend	All cause mortality	wholeGrain_t5		394573	16561	0.96 (0.95; 0.97)
trend	All cause mortality	vegTotal_t5		403870	17146	0.98 (0.97; 0.99)
trend	All cause mortality	fruitTotal_t5		403900	17133	0.96 (0.95; 0.97)
trend	All cause mortality	fishTotal_t5		409245	17397	1.00 (0.99; 1.01)
trend	All cause mortality	processedmeat_5cat		409377	17407	1.09 (1.07; 1.12)
trend	All cause mortality	redMeat_5cat		409592	17421	1.05 (1.03; 1.07)
trend	All cause mortality	poultry_5cat		409460	17391	0.96 (0.94; 0.98)
trend	All cause mortality	egg_total_5cat		185022	5925	0.97 (0.95; 0.99)
trend	All cause mortality	pulses_5cat		178062	5415	0.96 (0.91; 1.02)
trend	All cause mortality	ssb_5cat		218275	7978	1.02 (0.98; 1.07)
trend	All cause mortality	nuts_total_5cat		178062	5415	0.92 (0.89; 0.96)
trend	All cause mortality	td_egg_total_5cat		169884	5523	0.99 (0.97; 1.01)
trend	All cause mortality	td_pulses_5cat		162652	5003	0.99 (0.94; 1.05)
trend	All cause mortality	td_ssb_5cat		204900	7639	1.02 (0.98; 1.07)
trend	All cause mortality	td_nuts_total_5cat		162652	5003	0.92 (0.88; 0.96)
trend	All cause mortality	tb_unSalnuts_total_5cat		162652	5003	0.91 (0.86; 0.96)
trend	All cause mortality	td_Salnuts_total_5cat		162652	5003	0.97 (0.93; 1.03)
category	All cause mortality	wholeGrain_t5 - 1	0	94976	5137	1.00 (1.00; 1.00)
category	All cause mortality	wholeGrain_t5 - 2	>0-25	77979	2540	0.78 (0.74; 0.81)
category	All cause mortality	wholeGrain_t5 - 3	>25-28	97102	3628	0.77 (0.73; 0.80)
category	All cause mortality	wholeGrain_t5 - 4	>28-36	47652	1902	0.82 (0.78; 0.86)
category	All cause mortality	wholeGrain_t5 - 5	>36	76864	3354	0.82 (0.79; 0.86)
category	All cause mortality	vegTotal_t5 - 1	0-36	128736	5660	1.00 (1.00; 1.00)
category	All cause mortality	vegTotal_t5 - 2	>36-47	56186	2329	0.95 (0.90; 0.99)
category	All cause mortality	vegTotal_t5 - 3	>17-60	82227	3449	0.94 (0.90; 0.98)
category	All cause mortality	vegTotal_t5 - 4	>60-77	60291	2482	0.92 (0.87; 0.96)
category	All cause mortality	vegTotal_t5 - 5	>77	76430	3226	0.93 (0.89; 0.97)
category	All cause mortality	fruitTotal_t5 - 1	0-100	110408	5518	1.00 (1.00; 1.00)
category	All cause mortality	fruitTotal_t5 - 2	>100-200	100701	4088	0.88 (0.84; 0.91)
category	All cause mortality	fruitTotal_t5 - 3	>200-204	37270	1375	0.84 (0.79; 0.89)
category	All cause mortality	fruitTotal_t5 - 4	>204-304	81129	3130	0.85 (0.81; 0.89)
category	All cause mortality	fruitTotal_t5 - 5	>304	74392	3022	0.86 (0.82; 0.90)

category	All cause mortality	fishTotal_t5 - 1	0-19	107479	4347	1.00 (1.00; 1.00)
category	All cause mortality	fishTotal_t5 - 2	>19-25	60661	2446	0.97 (0.92; 1.02)
category	All cause mortality	fishTotal_t5 - 3	>25-40	127692	5421	0.96 (0.92; 1.00)
category	All cause mortality	fishTotal_t5 - 4	>40-64	41715	1794	1.03 (0.98; 1.09)
category	All cause mortality	fishTotal_t5 - 5	>64	71698	3389	0.99 (0.94; 1.03)
category	All cause mortality	processedmeat_5cat - [0	0	38793	1362	1.00 (1.00; 1.00)
category	All cause mortality	processedmeat_5cat - [1	>0-6	245272	9806	1.02 (0.96; 1.08)
category	All cause mortality	processedmeat_5cat - [2	>6-38	109455	5297	1.13 (1.06; 1.20)
category	All cause mortality	processedmeat_5cat - [3	>38-70	12630	717	1.25 (1.14; 1.37)
category	All cause mortality	processedmeat_5cat - [4	>70	3227	225	1.47 (1.27; 1.69)
category	All cause mortality	redMeat_5cat - [0] Neve	0	28862	951	1.00 (1.00; 1.00)
category	All cause mortality	redMeat_5cat - [1] 1 or l	>0-34	177118	7071	1.02 (0.95; 1.09)
category	All cause mortality	redMeat_5cat - [2] 2-4 p	>34-85	183518	8243	1.05 (0.99; 1.13)
category	All cause mortality	redMeat_5cat - [3] 5-6 p	>85-120	13855	785	1.18 (1.07; 1.29)
category	All cause mortality	redMeat_5cat - [4] Daily	>120	6239	371	1.21 (1.08; 1.37)
category	All cause mortality	poultry_5cat - [0] Never	0	21598	816	1.00 (1.00; 1.00)
category	All cause mortality	poultry_5cat - [1] 1 or le	>0-55	190020	9140	0.97 (0.90; 1.04)
category	All cause mortality	poultry_5cat - [2] 2-4 p/	>55-102	188633	7103	0.91 (0.85; 0.98)
category	All cause mortality	poultry_5cat - [3] 5-6 p w	>102-130	8081	291	1.00 (0.88; 1.15)
category	All cause mortality	poultry_5cat - [4] Daily	>130	1128	41	0.97 (0.71; 1.33)
category	All cause mortality	egg_total_5cat - [0] nev	0	121214	3983	1.00 (1.00; 1.00)
category	All cause mortality	egg_total_5cat - [1] half	half <25g	10489	286	0.82 (0.73; 0.93)
category	All cause mortality	egg_total_5cat - [2] 1 50	150g	18086	518	0.85 (0.78; 0.93)
category	All cause mortality	egg_total_5cat - [3] 2-3	50-150g	28690	891	0.90 (0.83; 0.96)
category	All cause mortality	egg_total_5cat - [4] 3 or	>150	6543	247	1.08 (0.95; 1.23)
category	All cause mortality	pulses_5cat - [0] never	0	147786	4607	1.00 (1.00; 1.00)
category	All cause mortality	pulses_5cat - [1] half	>0-37	24645	647	0.91 (0.83; 0.98)
category	All cause mortality	pulses_5cat - [2] 1 servin	>37-82	5081	146	1.02 (0.87; 1.21)
category	All cause mortality	pulses_5cat - [3] 2-3servi	>82-160	511	14	1.02 (0.60; 1.72)
category	All cause mortality	pulses_5cat - [4] 3 or mo	>160	39	1	0.72 (0.10; 5.11)
category	All cause mortality	ssb_5cat - [0] never	0	192115	7177	1.00 (1.00; 1.00)
category	All cause mortality	ssb_5cat - [1] half	125	15813	452	0.91 (0.83; 1.00)
category	All cause mortality	ssb_5cat - [2] 1 serving	250	7520	240	1.02 (0.90; 1.16)

category	All cause mortality	ssb_5cat - [3] 2-3serving	>500-750	2207	81	1.22 (0.98; 1.52)
category	All cause mortality	ssb_5cat - [4] 3 or more	>750	620	28	1.59 (1.10; 2.31)
category	All cause mortality	nuts_total_5cat - [0] nev	0	137738	4348	1.00 (1.00; 1.00)
category	All cause mortality	nuts_total_5cat - [1] half	half <20g	26632	683	0.82 (0.76; 0.89)
category	All cause mortality	nuts_total_5cat - [2] 1 4(	40g	9766	276	0.91 (0.80; 1.03)
category	All cause mortality	nuts_total_5cat - [3] 2-3	80-120g	3851	106	0.89 (0.73; 1.08)
category	All cause mortality	nuts_total_5cat - [4] 3 or	>120g	75	2	0.81 (0.20; 3.24)
category	All cause mortality	td_egg_total_5cat - [0] r	0	118342	3843	1.00 (1.00; 1.00)
category	All cause mortality	td_egg_total_5cat - [1] h	half <25g	6632	201	0.89 (0.77; 1.03)
category	All cause mortality	td_egg_total_5cat - [2] 1	150g	13192	400	0.89 (0.80; 0.98)
category	All cause mortality	td_egg_total_5cat - [3] 2	50-150g	25686	855	0.97 (0.90; 1.05)
category	All cause mortality	td_egg_total_5cat - [4] 3	>150	6032	224	1.08 (0.94; 1.23)
category	All cause mortality	td_pulses_5cat - [0] nev	0	136997	4286	1.00 (1.00; 1.00)
category	All cause mortality	td_pulses_5cat - [1] half	>0-37	19797	543	0.94 (0.86; 1.03)
category	All cause mortality	td_pulses_5cat - [2] 1 se	>37-82	5253	159	1.09 (0.93; 1.27)
category	All cause mortality	td_pulses_5cat - [3] 2-3s	>82-160	563	14	0.96 (0.57; 1.62)
category	All cause mortality	td_pulses_5cat - [4] 3 or	>160	42	1	0.64 (0.09; 4.52)
category	All cause mortality	td_ssb_5cat - [0] never	0	184755	7000	1.00 (1.00; 1.00)
category	All cause mortality	td_ssb_5cat - [1] half	125	11431	335	0.89 (0.80; 1.00)
category	All cause mortality	td_ssb_5cat - [2] 1 servin	250	6350	208	1.02 (0.88; 1.17)
category	All cause mortality	td_ssb_5cat - [3] 2-3serv	>500-750	1843	72	1.19 (0.94; 1.50)
category	All cause mortality	td_ssb_5cat - [4] 3 or mc	>750	521	24	1.60 (1.07; 2.39)
category	All cause mortality	td_nuts_total_5cat - [0]	0	128196	4071	1.00 (1.00; 1.00)
category	All cause mortality	td_nuts_total_5cat - [1] l	half <20g	21696	585	0.85 (0.78; 0.93)
category	All cause mortality	td_nuts_total_5cat - [2] :	40g	8981	242	0.85 (0.75; 0.97)
category	All cause mortality	td_nuts_total_5cat - [3] :	80-120g	3705	102	0.88 (0.72; 1.07)
category	All cause mortality	td_nuts_total_5cat - [4] :	>120g	74	3	1.22 (0.39; 3.78)
category	All cause mortality	RefinedGrains_5cat		392084	16607	1.04 (1.03; 1.05)
category	All cause mortality	Dairy_intake_5cat		408199	17321	0.98 (0.96; 1.00)
category	All cause mortality	RefinedGrains_5cat - [0]	0	167184	5934	1.00 (1.00; 1.00)
category	All cause mortality	RefinedGrains_5cat - [1]	>0 to 15g	24190	1048	1.20 (1.12; 1.28)
category	All cause mortality	RefinedGrains_5cat - [2]	>15 to 30	47213	2002	1.17 (1.11; 1.23)
category	All cause mortality	RefinedGrains_5cat - [3]	>30 to 50	63414	3252	1.23 (1.18; 1.28)

category	All cause mortality	RefinedGrains_5cat - [4]	>50g	90083	4371	1.16 (1.11; 1.21)
category	All cause mortality	Dairy_intake_5cat - [0] n	0	4488	187	1.00 (1.00; 1.00)
category	All cause mortality	Dairy_intake_5cat - [1] >	>0 to 125	28795	1180	0.99 (0.85; 1.16)
category	All cause mortality	Dairy_intake_5cat - [2] >	>125 to 255	73930	3395	0.98 (0.85; 1.13)
category	All cause mortality	Dairy_intake_5cat - [3] >	>255 to 280	250504	10617	0.95 (0.82; 1.10)
category	All cause mortality	Dairy_intake_5cat - [4] >	>280	50482	1942	0.93 (0.80; 1.08)

Table S3. Table presenting correlation between food intake of the different food groups for the UK Biobank cohort.

	Fish	Oily Fish	Vegetables	Fruits	Dairy	whole Grain	Refined Grains	Eggs	Fizzy	Poultry	Processed meat	Red Meat
Pulses	0.04	0.04	0.08	0.05	0.04	0.02	-0.04	-0.01	-0.01	-0.05	-0.06	-0.05
Fish		0.81	0.17	0.17	0.14	0.06	-0.07	0.03	-0.04	0.07	-0.05	-0.01
Oily Fish			0.16	0.15	0.14	0.04	-0.06	0.03	-0.02	0.04	-0.07	-0.02
Vegetables				0.23	0.25	0.03	-0.07	0.03	-0.01	0.02	-0.11	-0.01
Fruits					0.86	0.08	-0.09	-0.01	-0.05	-0.01	-0.15	-0.06
Dairy						0.06	-0.07	-0.01	-0.03	-0.01	-0.14	-0.05
whole Grain							0.14	-0.01	-0.03	0	0	-0.03
Refined Grains								0.01	0.03	0.01	0.13	0.05
Eggs									0.02	0.01	0.05	0.03
Fizzy										0.03	0.06	0.03
Poultry											0.16	0.2
Processed meat												0.23

Table S4. Intake categories per food group for each quintile (Q1-Q5) among people in the UK based on data from UK Biobank.

	Q1 (lowest)	Q2	Q3 (typical)	Q4	Q5 (highest)
Whole grains	0 g/d	>0-25 g/d	>25-28 g/d	>28-36 g/d	>36 g/d
Vegetables	0-36 g/d	>36-47 g/d	>47-60 g/d	>60-77 g/d	>77 g/d
Fruit	0-100 g/d	>100-200 g/d	>200-204 g/d	>204-304 g/d	>304 g/d
Nuts	0 g/d	<20 g/d	40 g/d	80-120 g/d	>120 g/d
Legumes	0 g/d	>0-37 g/d	>37-82 g/d	>82-160 g/d	>160 g/d
Fish	0-19 g/d	>19-25 g/d	>25-40 g/d	>40-64 g/d	>64 g/d
Egg	0 g/d	<25 g/d	25-50 g/d	50-150 g/d	>150 g/d
Milk	0 g/d	>0-125 g/d	>125-255 g/d	>255-280 g/d	>280 g/d
Refined grains	0 g/d	>0-15 g/d	>15-30 g/d	>30-50 g/d	>50 g/d
Meat, red	0 g/d	>0-34 g/d	>34-85 g/d	>85-120 g/d	>120 g/d
Meat, processed	0 g/d	>0-6 g/d	>6-38 g/d	>38-70 g/d	>70 g/d
Meat, white	0 g/d	>0-55 g/d	>55-102 g/d	>102-130 g/d	>130 g/d
Sugar-sweetened beverages	0 ml/d	125 ml/d	250 ml/d	>500-750 ml/d	>750 ml/d

* Data presented as daily intakes for each of the food quintiles. The unhealthy categories are labelled with red, the longevity-associated are labelled with green/dark green and the Eatwell recommendations in blue.

Table S5. Sensitivity analysis assessing potential mediation by energy and body mass index with associations from UK Biobank between various food groups with all-cause mortality hazard ratios. a. First presented hazard ratios for models with core adjustment including age, sex, socio-demographic area, smoking, alcohol consumption, and activity level. b. Then adding core adjustment when adding adjustments body mass index, and also core adjustment when adding adjustments for energy and body mass index (b). Model a included up to 409,592 participants, model b included up to 367,501 participants, and model c included up to 162,112 participants.

a. First presented hazard ratios for models with core adjustment including age, sex, socio-demographic area, smoking, alcohol consumption, and activity level (n=409,592 participants).

	Q1 (lowest)	Q2	Q3 (typical)	Q4	Q5 (highest)
Whole grains	1	0.78 (0.74-0.81)	0.77 (0.73-0.8)	0.82 (0.78-0.86)	0.82 (0.79-0.86)
Vegetables	1	0.95 (0.9-0.99)	0.94 (0.9-0.98)	0.92 (0.87-0.96)	0.93 (0.89-0.97)
Fruit	1	0.88 (0.84-0.91)	0.84 (0.79-0.89)	0.85 (0.81-0.89)	0.86 (0.82-0.9)
Nuts	1	0.82 (0.76-0.89)	0.91 (0.8-1.03)	0.89 (0.73-1.08)	0.81 (0.2-3.24)
Legumes	1	0.91 (0.83-0.98)	1.02 (0.87-1.21)	1.02 (0.6-1.72)	0.72 (0.1-5.11)
Fish	1	0.97 (0.92-1.02)	0.96 (0.92-1.00)	1.03 (0.98-1.09)	0.99 (0.94-1.03)
Egg	1	0.82 (0.73-0.93)	0.85 (0.78-0.93)	0.90 (0.83-0.96)	1.08 (0.95-1.23)
Milk	1	0.99 (0.85-1.16)	0.98 (0.85-1.13)	0.95 (0.82-1.1)	0.93 (0.8-1.08)
Refined grains	1	1.20 (1.12-1.28)	1.17 (1.11-1.23)	1.23 (1.18-1.28)	1.16 (1.11-1.21)
Meat, red	1	1.02 (0.95-1.09)	1.05 (0.99-1.13)	1.18 (1.07-1.29)	1.21 (1.08-1.37)
Meat, processed	1	1.02 (0.96-1.08)	1.13 (1.06-1.2)	1.25 (1.14-1.37)	1.47 (1.27-1.69)
Meat, white	1	0.97 (0.90-1.04)	0.91 (0.85-0.98)	1.00 (0.88-1.15)	0.97 (0.71-1.33)
Sugar-sweetened beverages	1	0.91 (0.83-1)	1.02 (0.9-1.16)	1.22 (0.98-1.52)	1.59 (1.1-2.31)

b. Adjustment for body mass index, age, sex, socio-demographic area, smoking, alcohol consumption, and activity level (n=367,501).

	Q1 (lowest)	Q2	Q3 (typical)	Q4	Q5 (highest)
Whole grains	1	0.81 (0.76; 0.85)	0.82 (0.78; 0.86)	0.83 (0.78; 0.87)	0.84 (0.81; 0.89)
Vegetables	1	0.99 (0.94; 1.04)	0.98 (0.93; 1.02)	0.97 (0.92; 1.02)	0.98 (0.94; 1.03)
Fruit	1	0.91 (0.87; 0.95)	0.90 (0.84; 0.96)	0.90 (0.86; 0.94)	0.92 (0.87; 0.97)
Nuts	1	0.82 (0.75; 0.89)	0.93 (0.81; 1.05)	0.90 (0.73; 1.10)	0.45 (0.06; 3.22)
Legumes	1	0.92 (0.84; 1.00)	1.05 (0.88; 1.25)	1.17 (0.69; 1.98)	0.86 (0.12; 6.11)
Fish	1	0.96 (0.91; 1.02)	0.99 (0.95; 1.04)	1.08 (1.02; 1.14)	1.06 (1.01; 1.11)
Egg	1	0.83 (0.74; 0.95)	0.88 (0.80; 0.96)	0.89 (0.82; 0.96)	1.03 (0.89; 1.18)
Milk	1	0.99 (0.94; 1.03)	0.94 (0.91; 0.98)	0.94 (0.90; 0.99)	0.94 (0.89; 0.99)
Refined grains	1	1.07 (1.04; 1.09)	1.13 (1.08; 1.19)	1.17 (1.12; 1.23)	1.14 (1.09; 1.19)
Meat, red	1	0.98 (0.90; 1.06)	0.99 (0.92; 1.08)	1.06 (0.95; 1.19)	1.14 (0.99; 1.30)
Meat, processed	1	0.98 (0.92; 1.05)	1.05 (0.98; 1.13)	1.16 (1.04; 1.29)	1.33 (1.13; 1.57)
Meat, white	1	0.85 (0.78; 0.93)	0.80 (0.74; 0.87)	0.92 (0.79; 1.06)	0.92 (0.66; 1.29)
Sugar-sweetened beverages	1	0.91 (0.83; 1.01)	1.02 (0.89; 1.17)	1.19 (0.94; 1.51)	1.51 (1.00; 2.27)

c. Adjustment for body mass index, energy, age, sex, socio-demographic area, smoking, alcohol consumption, and activity level (n=162,112).

	Q1 (lowest)	Q2	Q3 (typical)	Q4	Q5 (highest)
Whole grains	1	0.81 (0.73; 0.89)	0.82 (0.74; 0.90)	0.86 (0.77; 0.95)	0.92 (0.84; 1.00)
Vegetables	1	1.05 (0.96; 1.15)	1.04 (0.96; 1.13)	0.96 (0.88; 1.06)	0.97 (0.89; 1.06)
Fruit	1	0.95 (0.87; 1.02)	0.91 (0.82; 1.02)	0.90 (0.82; 0.98)	0.93 (0.85; 1.01)
Nuts	1	0.82 (0.75; 0.89)	0.92 (0.81; 1.05)	0.89 (0.73; 1.10)	0.45 (0.06; 3.18)
Legumes	1	0.95 (0.87; 1.05)	1.13 (0.96; 1.34)	1.08 (0.64; 1.82)	0.74 (0.10; 5.25)
Fish	1	1.01 (0.92; 1.11)	1.04 (0.96; 1.12)	1.09 (0.97; 1.21)	1.03 (0.94; 1.12)
Egg	1	0.86 (0.76; 0.98)	0.91 (0.82; 1.00)	0.92 (0.85; 1.00)	1.07 (0.93; 1.23)
Milk	1	0.99 (0.91; 1.08)	0.92 (0.85; 0.98)	0.90 (0.83; 0.98)	0.89 (0.81; 0.98)
Refined grains	1	1.08 (1.03; 1.13)	1.16 (1.07; 1.26)	1.14 (1.06; 1.23)	1.13 (1.05; 1.23)
Meat, red	1	1.00 (0.87; 1.15)	1.02 (0.89; 1.17)	1.00 (0.82; 1.23)	0.94 (0.72; 1.23)
Meat, processed	1	1.05 (0.93; 1.18)	1.07 (0.94; 1.21)	1.09 (0.90; 1.33)	1.45 (1.08; 1.94)
Meat, white	1	0.89 (0.77; 1.03)	0.84 (0.72; 0.97)	1.04 (0.80; 1.34)	0.44 (0.16; 1.19)
Sugar-sweetened beverages	1	1.00 (0.90; 1.11)	1.12 (0.98; 1.29)	1.31 (1.03; 1.66)	1.65 (1.09; 2.49)

Table S6. Sensitivity analysis of all-cause mortality hazard ratios for various food groups with uncertainty limits for the UK based on estimates from UK Biobank using time-to-full-effect of 5, 20 and 50 years, and model adjustments of 0.25, 0.5 and 1.0. The rightmost column shows the % change when comparing age- and sex-specific numbers in the UUK->HUK column to results from the main analyses (m=0.5 and t=20).
MUK: Median UK diet; UUK: Unhealthy UK diet; EUK: Eatwell Guideline diet; HUK: Healthy UK diet; m: model adjustment parameter; t: time to full effect of new diet.

		Life expectancy		Life expectancy		Life expectancy gain from sustained diet change				% change
	Age	MUK	UUK	EUK	HUK	MUK->HUK	UUK->HUK	MUK->EUK	UUK->EUK	UUK->HUK
UK females (m=0.5, t=20)	40	44.7	37.4	46.0	47.8	3.1 (1.3;4.9)	10.4 (8.2;11.3)	1.3 (0.1;2.4)	8.6 (6.8;10.2)	0
	70	17.6	13.9	18.4	19.3	1.7 (0.7;2.6)	5.4 (4.2;6.3)	0.7 (0.0;1.3)	4.4 (3.6;5.4)	0
UK males (m=0.5, t=20)	40	41.5	34.0	42.9	44.8	3.4 (1.4;5.3)	10.8 (8.8;12.0)	1.4 (0.1;2.6)	8.9 (7.2;10.8)	0
	70	15.5	12.2	16.2	17.1	1.6 (0.7;2.5)	5.0 (4.2;5.6)	0.7 (0.0;1.2)	4.0 (3.4;5.1)	0
UK females (m=0.25, t=20)	40	44.7	41.1	45.3	46.2	1.6 (0.7;2.4)	5.1 (4.0;5.5)	0.7 (0.0;1.2)	4.2 (3.3;5.0)	-51
	70	17.6	15.8	18.0	18.5	0.8 (0.4;1.3)	2.7 (2.2;3.0)	0.4 (0.0;0.6)	2.2 (1.8;2.7)	-50
UK males (m=0.25, t=20)	40	41.5	37.8	42.2	43.1	1.7 (0.7;2.6)	5.3 (4.3;5.9)	0.7 (0.0;1.3)	4.4 (3.5;5.3)	-51
	70	15.5	13.8	15.8	16.3	0.8 (0.3;1.2)	2.5 (2.1;2.8)	0.3 (0.0;0.6)	2.0 (1.7;2.5)	-50
UK females (m=0.75, t=20)	40	44.7	33.5	46.7	49.3	4.6 (2.0;7.4)	15.8 (12.5;17.1)	2.0 (0.1;3.6)	13.2(10.3;15.7)	52
	70	17.6	12.2	18.7	20.1	2.5 (1.1;4.0)	7.9 (6.6;8.8)	1.1 (0.1;1.9)	6.5 (5.5;8.1)	46
UK males (m=0.75, t=20)	40	41.5	30.1	43.6	46.5	5.1 (2.2;8.0)	16.4 (13.4;18.2)	2.2 (0.1;3.9)	13.5(11.0;16.6)	52
	70	15.5	10.6	16.5	17.9	2.4 (1.0;3.8)	7.3 (6.2;8.3)	1.0 (0.1;1.9)	5.9 (5.1;7.6)	46
UK females (m=1.0, t=20)	40	44.7	29.3	47.3	50.7	6.0 (2.6;9.7)	21.4 (16.7;23.1)	2.7 (0.1;4.7)	18.0(13.9;21.5)	106
	70	17.6	10.6	19.1	20.9	3.3 (1.4;5.3)	10.4 (8.7;11.5)	1.4 (0.1;2.6)	8.5 (7.3;10.8)	93
UK males (m=1.0, t=20)	40	41.5	26.1	44.3	48.2	6.7 (2.9;10.7)	22.1 (18;24.6)	2.9 (0.1;5.3)	18.3(14.8;22.7)	105
	70	15.5	9.2	16.9	18.7	3.2 (1.4;5.1)	9.5 (8.3;10.9)	1.4 (0.1;2.5)	7.7 (6.8;10.1)	90

UK females (m=0.5, t=5)	40	44.7	37.0	46.1	47.9	3.2 (1.4;5.1)	10.9 (8.6;11.8)	1.4 (0.1;2.5)	9.1 (7.1;10.7)	5
	70	17.6	12.6	18.6	20.0	2.3 (1.0;3.7)	7.4 (6.1;8.3)	1.0 (0.1;1.8)	6.1 (5.0;7.5)	37
UK males (m=0.5, t=5)	40	41.5	33.5	43.0	45.0	3.6 (1.5;6.0)	11.5 (9.3;12.7)	1.5 (0.1;2.7)	9.5 (7.6;11.5)	6
	70	15.5	10.7	16.5	17.9	2.4 (1.0;3.7)	7.3 (6.2;8.4)	1.0 (0.1;1.9)	5.8 (5.0;7.5)	46
UK females (m=0.5, t=50)	40	44.7	38.8	45.7	47.2	2.5 (1.1;4.0)	8.4 (6.7;9.1)	1.1 (0.1;1.9)	7.0 (5.5;8.3)	-19
	70	17.6	15.7	17.9	18.3	0.7 (0.3;1.1)	2.6 (1.9;2.7)	0.3 (0.0;0.5)	2.3 (1.6;2.4)	-52
UK males (m=0.5, t=50)	40	41.5	35.6	42.6	44.1	2.6 (1.1;4.1)	8.4 (6.8;9.3)	1.1 (0.1;2.0)	6.9 (5.6;8.4)	-22
	70	15.5	13.8	15.8	16.1	0.6 (0.3;1.0)	2.3 (1.7;2.4)	0.3 (0.0;0.5)	2.0 (1.4;2.2)	-54

Table S7. Number of food frequency questionnaires and presentation of UK Biobank data.

**Baselines food frequency questionnaires** (2007-2009)		**420,766**
**Web-based 24-h dietary assessments**		**183,380**
-	Cycle 0 (2009-2010)	61,013
-	Cycle 1 (Feb-Apr.2011)	87,925
-	Cycle 2 (Jun-Sep.2011)	72,650
-	Cycle 3 (Oct-Dec.2011)	90,830
-	Cycle 4 (Apr-Jun.2012)	87,812

Table S8. Background characteristics of participants.

	Females (N=235,315)	Males (N=185,451)	Overall (N=420,766)
**Age, mean (SD)**	56.3 (7.98)	56.4 (8.19)	56.3 (8.07)
Females/males	235,315 (55.9%)	185,451 (44.1%)	420,766 (100.0%)
**Townsend deprivation index**			
Lower deprivation	80,734 (34.3%)	64,876 (35.0%)	145,610 (34.6%)
Middle	80,317 (34.1%)	61,940 (33.4%)	142,257 (33.8%)
Higher deprivation	74,264 (31.6%)	58,635 (31.6%)	132,899 (31.6%)
**Ethnicity**			
White	223,456 (95.0%)	176,023 (94.9%)	399,479 (94.9%)
Mixed	3,497 (1.5%)	2,244 (1.2%)	5,741 (1.4%)
South Asian	3,588 (1.5%)	3,687 (2.0%)	7,275 (1.7%)
Black	3,469 (1.5%)	2,417 (1.3%)	5,886 (1.4%)
Chinese	751 (0.3%)	395 (0.2%)	1,146 (0.3%)
Any other	0 (0.0%)	0 (0.0%)	0 (0.0%)
Missing	554 (0.2%)	685 (0.4%)	1239 (0.3%)
**Height in meters, mean (SD)**	1.6 (0.06)	1.8 (0.07)	1.7 (0.09)
**Weight in kg, mean (SD)**	71.2 (13.9)	85.7 (14.1)	77.6 (15.7)
**Waist width in , mean (SD)**	84.4 (12.4)	96.5 (11.1)	89.7 (13.3)
**Body Mass index (kg/m2)**	27.0 (5.1)	27.7 (4.2)	27.3 (4.7)
Underweight	1,758 (0.7%)	424 (0.2%)	2,182 (0.5%)
Normal weight	93,098 (39.6%)	47,953 (25.9%)	141,051 (33.5%)
Overweight	86,377 (36.7%)	92,069 (49.6%)	178,446 (42.4%)
Obese	53,167 (22.6%)	44,186 (23.8%)	97,353 (23.1%)
Missing	915 (0.4%)	819 (0.4%)	1734 (0.4%)
**Smoking**			
Never	142,070 (60.4%)	94,532 (51.0%)	236,602 (56.2%)
Previous	73,803 (31.4%)	69,470 (37.5%)	143,273 (34.1%)
Current	19,442 (8.3%)	21,449 (11.6%)	40,891 (9.7%)
**Alcohol intake**			
Daily or almost daily	38,083 (16.2%)	47,186 (25.4%)	85,269 (20.3%)
3-4 times a week	49,659 (21.1%)	49,897 (26.9%)	99,556 (23.7%)
Once or twice a week	61,699 (26.2%)	48,818 (26.3%)	110,517 (26.3%)
1-3 times a month	30,844 (13.1%)	16,418 (8.9%)	47,262 (11.2%)

Special occasions only	34,154 (14.5%)	12,638 (6.8%)	46,792 (11.1%)
Never	20,876 (8.9%)	10,494 (5.7%)	31,370 (7.5%)
**Physical activity**			
Walking for pleasure	173,947 (73.9%)	128,827 (69.5%)	302,774 (72.0%)
Other exercises swimming, cycling, keep fit, bowling)	26,753 (11.4%)	25,200 (13.6%)	51,953 (12.3%)
Strenuous sports	802 (0.3%)	2,503 (1.3%)	3,305 (0.8%)
Light home activities: pruning, watering the lawn	14,724 (6.3%)	12,456 (6.7%)	27,180 (6.5%)
Heavy home activities: weeding, lawn mowing, carpentry, digging	3,269 (1.4%)	7,045 (3.8%)	10,314 (2.5%)
Missing	15820 (6.7%)	9420 (5.1%)	25240 (6.0%)

Table S9. Sex-stratified examples for continuous and categorical estimates of food groups and associations to mortality (adjusted for age, sex, area-based socio-demographic deprivation, smoking, alcohol consumption, and physical activity level, body mass index and energy consumption).

Continuous	Both	Females	Males
Whole grains	HR 0.98 (0.96-1.00)	HR 0.99 (0.95-1.02)	HR 0.97 (0.95-1.00)
Vegetables	HR 0.99 (0.97-1.01)	HR 0.98 (0.95-1.02)	HR 0.99 (0.97-1.02)
Fruit	HR 0.98 (0.96-1.00)	HR 0.98 (0.95-1.02)	HR 0.98 (0.95-1.01)
Fish	HR 1.01 (0.99-1.03)	HR 1.01 (0.98-1.05)	HR 1.01 (0.98-1.04)
Meat, processed	HR 1.04 (1.00-1.09)	HR 1.05 (0.97-1.13)	HR 1.06 (1.00-1.12)
Meat, red	HR 1.00 (0.96-1.05)	HR 1.00 (0.93-1.07)	HR 0.99 (0.94-1.05)
Refined grains	HR 1.04 (1.02-1.06)	HR 1.03 (1.00-1.06)	HR 1.04 (1.01-1.06)
Milk	HR 0.97 (0.95-0.99)	HR 0.98 (0.94-1.01)	HR 0.97 (0.94-1.00)
Meat, white	HR 0.95 (0.90-0.99)	HR 0.90 (0.83-0.97)	HR 0.97 (0.90-1.03)
Egg	HR 0.98 (0.96-1.00)	HR 0.98 (0.95-1.02)	HR 1.00 (0.97-1.03)
Legumes	HR 0.98 (0.92-1.04)	HR 0.98 (0.89-1.08)	HR 0.98 (0.90-1.07)
Sugar-sweetened beverages	HR 1.07 (1.02-1.12)	HR 1.03 (0.94-1.12)	HR 1.08 (1.02-1.15)
Nuts	HR 0.93 (0.88-0.97)	HR 0.90 (0.84-0.98)	HR 0.93 (0.88-0.99)

Categorical	Both	Females	Males
Whole grains (q1)	Reference	Reference	Reference
Whole grains (q2)	HR 0.81 (0.73-0.89)	HR 0.84 (0.72-0.99)	HR 0.81 (0.70-0.94)
Whole grains (q3)	HR 0.82 (0.74-0.90)	HR 0.89 (0.76-1.04)	HR 0.77 (0.67-0.88)
Whole grains (q4)	HR 0.86 (0.77-0.95)	HR 0.89 (0.75-1.06)	HR 0.80 (0.70-0.92)
Whole grains (q5)	HR 0.92 (0.84-1.00)	HR 0.91 (0.78-1.05)	HR 0.91 (0.81-1.02)
Vegetables (q1)	Reference	Reference	Reference
Vegetables (q2)	HR 1.05 (0.96-1.15)	HR 1.04 (0.90-1.21)	HR 1.03 (0.91-1.16)
Vegetables (q3)	HR 1.04 (0.96-1.13)	HR 0.99 (0.87-1.13)	HR 1.09 (0.98-1.21)
Vegetables (q4)	HR 0.96 (0.88-1.06)	HR 0.94 (0.81-1.08)	HR 0.96 (0.85-1.09)
Vegetables (q5)	HR 0.97 (0.89-1.06)	HR 0.96 (0.83-1.10)	HR 0.97 (0.86-1.10)

Fruit (q1)	Reference	Reference	Reference
Fruit (q2)	HR 0.95 (0.87-1.02)	HR 0.91 (0.79-1.05)	HR 0.98 (0.88-1.09)
Fruit (q3)	HR 0.91 (0.82-1.02)	HR 0.88 (0.74-1.05)	HR 0.94 (0.81-1.09)
Fruit (q4)	HR 0.90 (0.82-0.98)	HR 0.90 (0.78-1.04)	HR 0.89 (0.79-1.01)
Fruit (q5)	HR 0.93 (0.85-1.01)	HR 0.91 (0.79-1.06)	HR 0.95 (0.84-1.08)
Fish (q1)	Reference	Reference	Reference
Fish (q2)	HR 1.01 (0.92-1.11)	HR 1.03 (0.88-1.20)	HR 1.00 (0.88-1.14)
Fish (q3)	HR 1.04 (0.96-1.12)	HR 0.98 (0.86-1.11)	HR 1.07 (0.96-1.19)
Fish (q4)	HR 1.09 (0.97-1.21)	HR 1.15 (0.97-1.35)	HR 1.06 (0.91-1.23)
Fish (q5)	HR 1.03 (0.94-1.12)	HR 1.03 (0.89-1.19)	HR 1.02 (0.90-1.16)
Meat, processed (q1)	Reference	Reference	Reference
Meat, processed (q2)	HR 1.05 (0.93-1.18)	HR 1.06 (0.91-1.24)	HR 1.14 (0.93-1.40)
Meat, processed (q3)	HR 1.07 (0.94-1.21)	HR 1.10 (0.91-1.33)	HR 1.17 (0.95-1.45)
Meat, processed (q4)	HR 1.09 (0.90-1.33)	HR 0.98 (0.64-1.50)	HR 1.22 (0.94-1.60)
Meat, processed (q5)	HR 1.45 (1.08-1.94)	HR 1.69 (0.89-3.20)	HR 1.52 (1.05-2.20)
Meat, red (q1)	Reference	Reference	Reference
Meat, red (q2)	HR 1.00 (0.87-1.15)	HR 1.05 (0.87-1.28)	HR 1.01 (0.81-1.25)
Meat, red (q3)	HR 1.02 (0.89-1.17)	HR 1.03 (0.84-1.26)	HR 1.03 (0.82-1.28)
Meat, red (q4)	HR 1.00 (0.82-1.23)	HR 1.04 (0.75-1.45)	HR 1.03 (0.77-1.37)
Meat, red (q5)	HR 0.94 (0.72-1.23)	HR 1.10 (0.68-1.76)	HR 0.83 (0.57-1.20)
Refined grains (q1)	Reference	Reference	Reference
Refined grains (q2)	HR 1.08 (1.03-1.13)	HR 1.02 (0.95-1.10)	HR 1.13 (1.06-1.21)
Refined grains (q3)	HR 1.16 (1.07-1.26)	HR 1.04 (0.91-1.20)	HR 1.26 (1.12-1.41)
Refined grains (q4)	HR 1.14 (1.06-1.23)	HR 1.14 (1.01-1.29)	HR 1.12 (1.01-1.25)
Refined grains (q5)	HR 1.13 (1.05-1.23)	HR 1.10 (0.96-1.25)	HR 1.16 (1.04-1.28)
Milk (q1)	Reference	Reference	Reference
Milk (q2)	HR 0.99 (0.91-1.08)	HR 0.99 (0.87-1.13)	HR 1.01 (0.90-1.15)
Milk (q3)	HR 0.92 (0.85-0.98)	HR 0.92 (0.82-1.02)	HR 0.93 (0.84-1.03)
Milk (q4)	HR 0.90 (0.83-0.98)	HR 0.92 (0.81-1.05)	HR 0.92 (0.82-1.03)
Milk (q5)	HR 0.89 (0.81-0.98)	HR 0.93 (0.80-1.08)	HR 0.90 (0.79-1.03)

Meat, white (q1)	Reference	Reference	Reference
Meat, white (q2)	HR 0.89 (0.77-1.03)	HR 0.78 (0.63-0.95)	HR 1.10 (0.87-1.39)
Meat, white (q3)	HR 0.84 (0.72-0.97)	HR 0.72 (0.58-0.88)	HR 1.02 (0.80-1.29)
Meat, white (q4)	HR 1.04 (0.80-1.34)	HR 0.80 (0.54-1.19)	HR 1.31 (0.89-1.91)
Meat, white (q5)	HR 0.44 (0.16-1.19)	HR 0.21 (0.03-1.52)	HR 0.80 (0.25-2.53)
Egg (q1)	Reference	Reference	Reference
Egg (q2)	HR 0.86 (0.76-0.98)	HR 0.89 (0.73-1.07)	HR 0.86 (0.72-1.02)
Egg (q3)	HR 0.91 (0.82-1.00)	HR 1.01 (0.88-1.16)	HR 0.85 (0.74-0.97)
Egg (q4)	HR 0.92 (0.85-1.00)	HR 0.91 (0.80-1.04)	HR 0.98 (0.88-1.08)
Egg (q5)	HR 1.07 (0.93-1.23)	HR 1.01 (0.78-1.30)	HR 1.17 (0.97-1.40)
Legumes (q1)	Reference	Reference	Reference
Legumes (q2)	HR 0.92 (0.84-1.00)	HR 0.95 (0.84-1.09)	HR 0.90 (0.80-1.02)
Legumes (q3)	HR 1.05 (0.88-1.25)	HR 1.13 (0.87-1.47)	HR 1.02 (0.79-1.30)
Legumes (q4)	HR 1.17 (0.69-1.98)	HR 0.41 (0.10-1.65)	HR 1.77 (0.98-3.20)
Legumes (q5)	HR 0.86 (0.12-6.12)	-	HR 1.96 (0.27-13.95)
Sugar-sweetened beverages (q1)	Reference	Reference	Reference
Sugar-sweetened beverages (q2)	HR 1.00 (0.90-1.11)	HR 1.10 (0.94-1.29)	HR 0.92 (0.80-1.06)
Sugar-sweetened beverages (q3)	HR 1.12 (0.98-1.29)	HR 0.96 (0.73-1.27)	HR 1.19 (1.00-1.41)
Sugar-sweetened beverages (q4)	HR 1.31 (1.03-1.66)	HR 0.99 (0.57-1.71)	HR 1.46 (1.10-1.94)
Sugar-sweetened beverages (q5)	HR 1.65 (1.09-2.49)	HR 1.45 (0.60-3.50)	HR 1.62 (0.94-2.81)
Nuts (q1)	Reference	Reference	Reference
Nuts (q2)	HR 0.82 (0.75-0.89)	HR 0.91 (0.81-1.03)	HR 0.72 (0.63-0.82)
Nuts (q3)	HR 0.92 (0.81-1.05)	HR 0.81 (0.65-1.02)	HR 0.96 (0.82-1.14)
Nuts (q4)	HR 0.89 (0.73-1.10)	HR 0.72 (0.46-1.13)	HR 0.96 (0.75-1.22)
Nuts (q5)	HR 0.45 (0.06-3.18)	-	HR 0.65 (0.09-4.63)

Fig S1. Expected life years gained per food group presented in forest plots with uncertain intervals for 40-year-old female adults from the UK who changes from a: median to Eatwell Guide patterns (upper left), b: median to healthiest (lower left), c: unhealthy to Eatwell Guide patterns (upper right), d: unhealthy to healthiest (lower right).

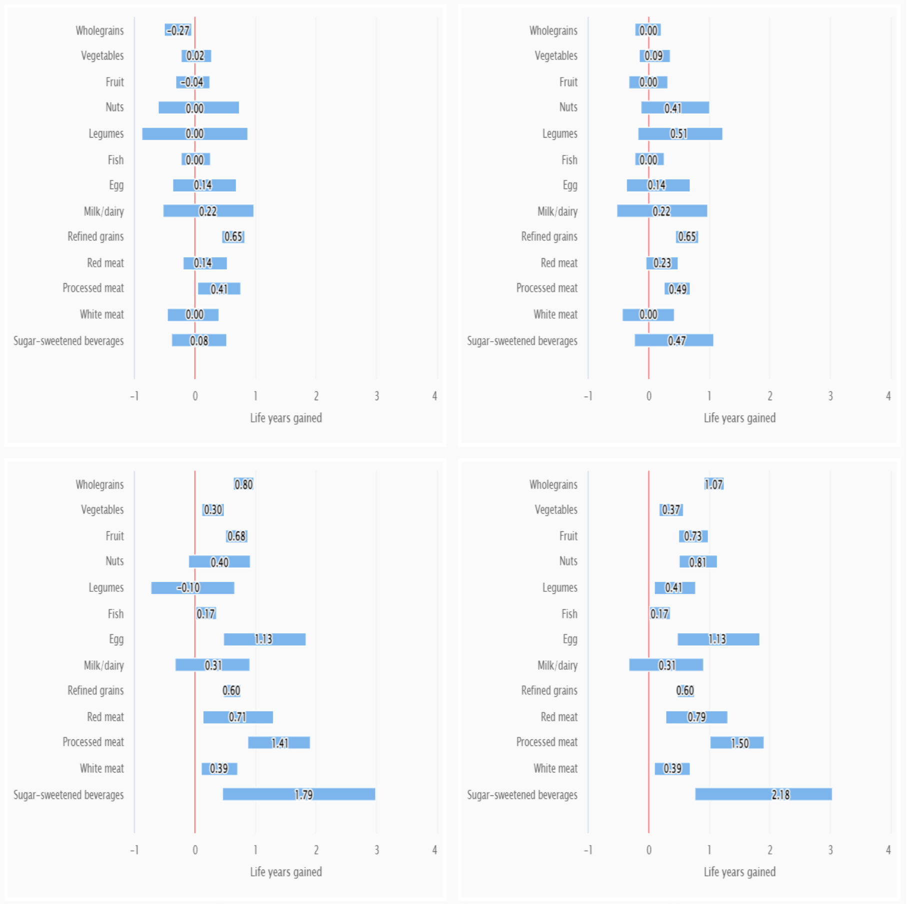

* Changes includes medium to high intake of whole grains, vegetables, fruits, nuts, legumes, fish, milk/dairy, and white meat, a low to medium intake of eggs, and a low intake of sugar-sweetened beverages, red and processed meats, and refined grains. For the last four groups, a reduction in the intake is associated with life expectancy gains. Estimated healthiest intake levels for legumes & nuts the 5th quintile, but due to wide confidence interval the second healthiest estimates for the 2nd quintile are presented.

Fig S2. Expected life years gained per food group presented in forest plots with uncertain intervals for 40-year-old male adults from the UK who changes from a: median to Eatwell Guide patterns (upper left), b: median to healthiest (lower left), c: unhealthy to Eatwell Guide patterns (upper right), d: unhealthy to healthiest (lower right).

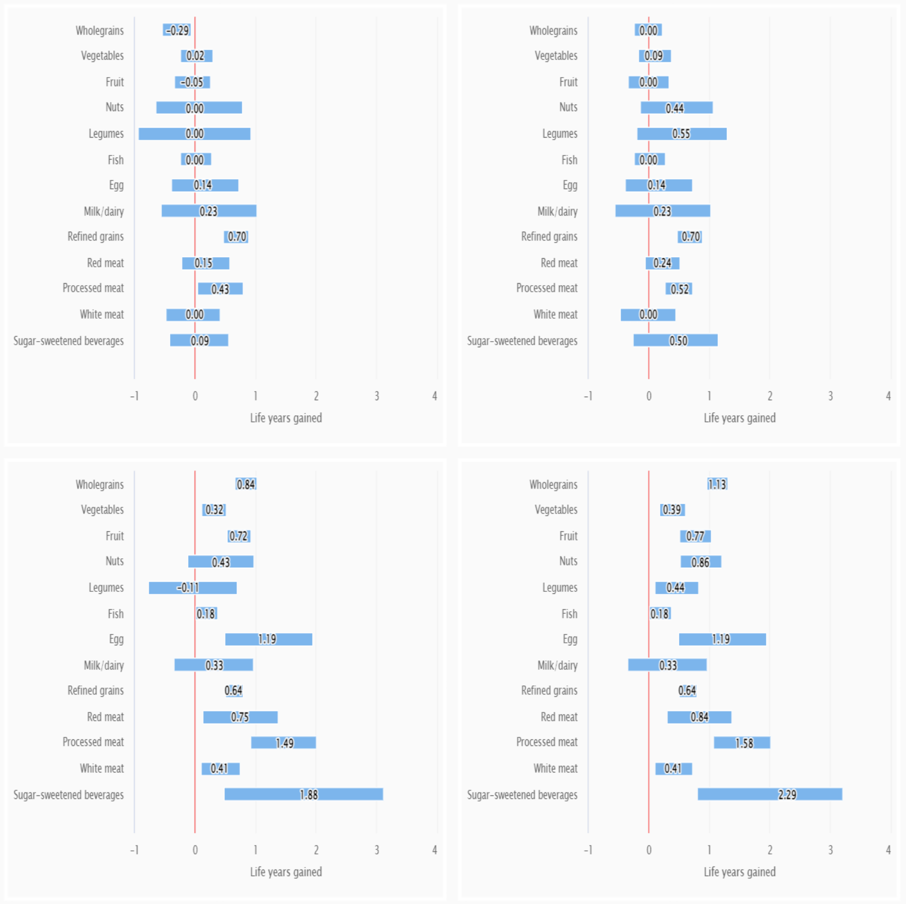

* Changes includes medium to high intake of whole grains, vegetables, fruits, nuts, legumes, fish, milk/dairy, and white meat, a low to medium intake of eggs, and a low intake of sugar-sweetened beverages, red and processed meats, and refined grains. For the last four groups, a reduction in the intake is associated with life expectancy gains. Estimated healthiest intake levels for legumes & nuts the 5th quintile, but due to wide confidence interval the second healthiest estimates for the 2nd quintile are presented.

Fig S3. Expected life years gained per food group presented in forest plots with uncertain intervals for 70-year-old female adults from the UK who changes from a: median to Eatwell Guide patterns (upper left), b: median to healthiest (lower left), c: unhealthy to Eatwell Guide patterns (upper right), d: unhealthy to healthiest (lower right).

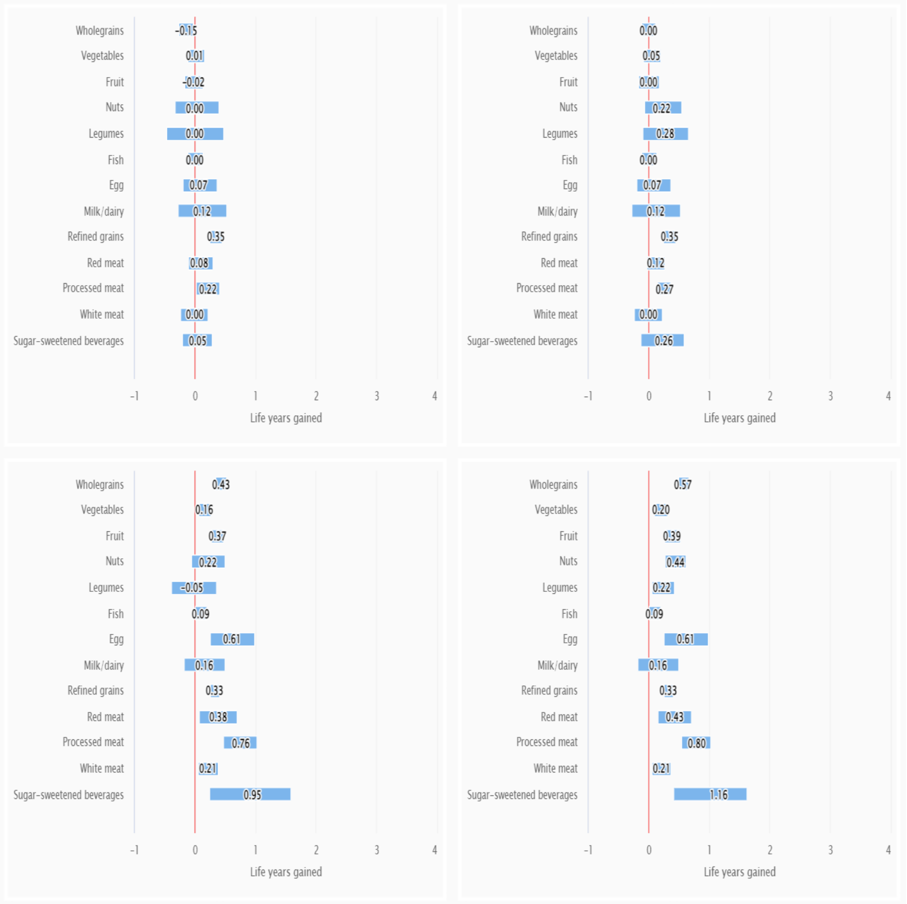

* Changes includes medium to high intake of whole grains, vegetables, fruits, nuts, legumes, fish, milk/dairy, and white meat, a low to medium intake of eggs, and a low intake of sugar-sweetened beverages, red and processed meats, and refined grains. For the last four groups, a reduction in the intake is associated with life expectancy gains. Estimated healthiest intake levels for legumes & nuts the 5th quintile, but due to wide confidence interval the second healthiest estimates for the 2nd quintile are presented.

Fig S4. Expected life years gained per food group presented in forest plots with uncertain intervals for 70-year-old male adults from the UK who changes from a: median to Eatwell Guide patterns (upper left), b: median to healthiest (lower left), c: unhealthy to Eatwell Guide patterns (upper right), d: unhealthy to healthiest (lower right).

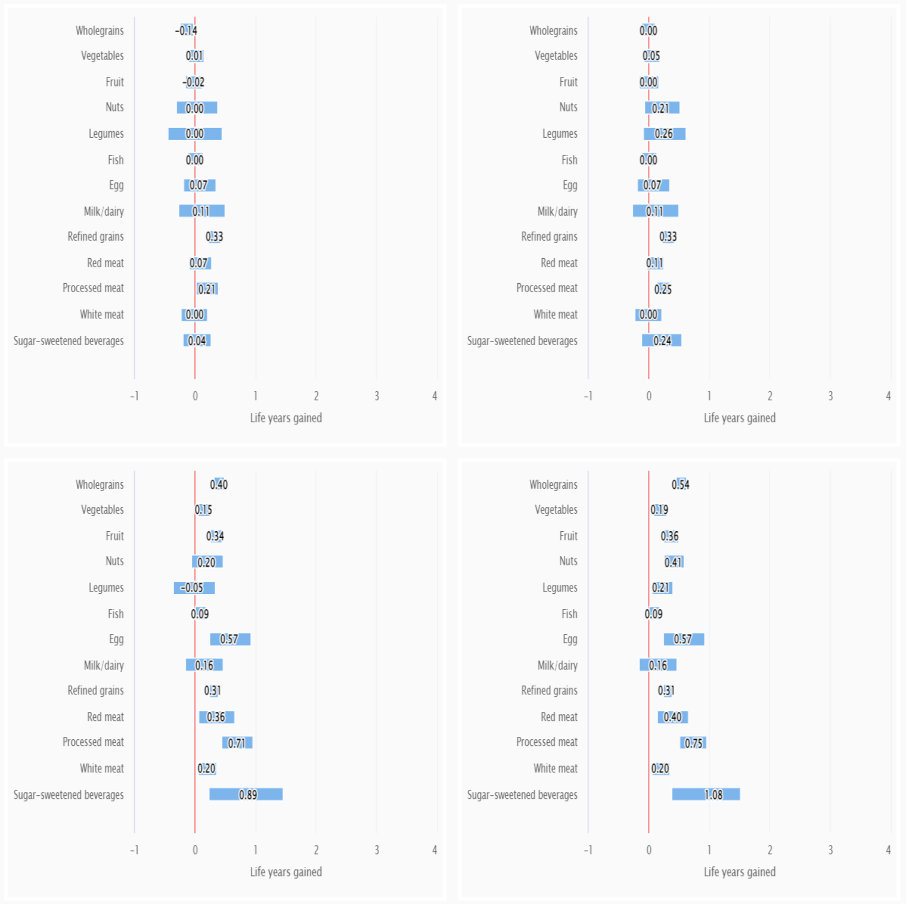

* Changes includes medium to high intake of whole grains, vegetables, fruits, nuts, legumes, fish, milk/dairy, and white meat, a low to medium intake of eggs, and a low intake of sugar-sweetened beverages, red and processed meats, and refined grains. For the last four groups, a reduction in the intake is associated with life expectancy gains. Estimated healthiest intake levels for legumes & nuts the 5th quintile, but due to wide confidence interval the second healthiest estimates for the 2nd quintile are presented.

Fig S5. Sensitivity analysis with expected life years gained per food group presented in forest plots with uncertain intervals for 40-year-old female adults from the UK who changes from median to healthiest diet patterns with a: time-to-full effect of 5 years (upper left), b: time-to-full effect of 50 years (lower left), c: conservative model with m=0.25 (upper right), d: model not assuming overlap with m=1.0.

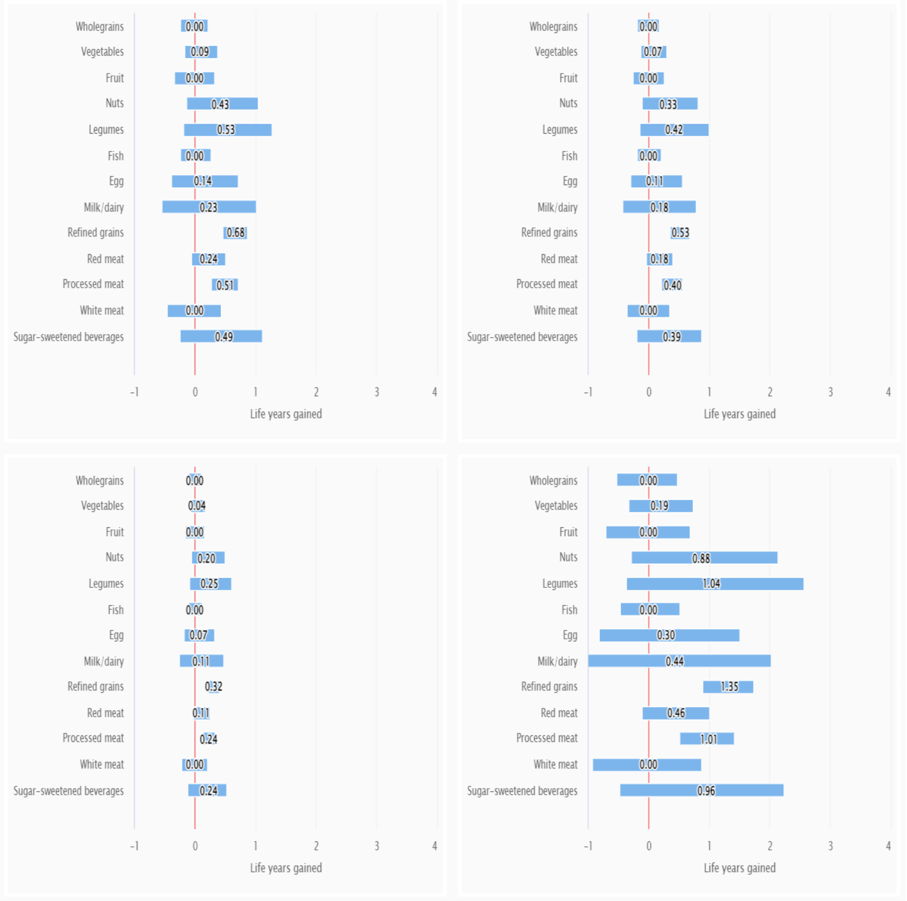

Fig S6. Sensitivity analysis with expected life years gained per food group presented in forest plots with uncertain intervals for 70-year-old male adults from the UK who changes from median to healthiest diet patterns with a: time-to-full effect of 5 years (upper left), b: time-to-full effect of 50 years (lower left), c: conservative model with m=0.25 (upper right), d: model not assuming overlap with m=1.0.

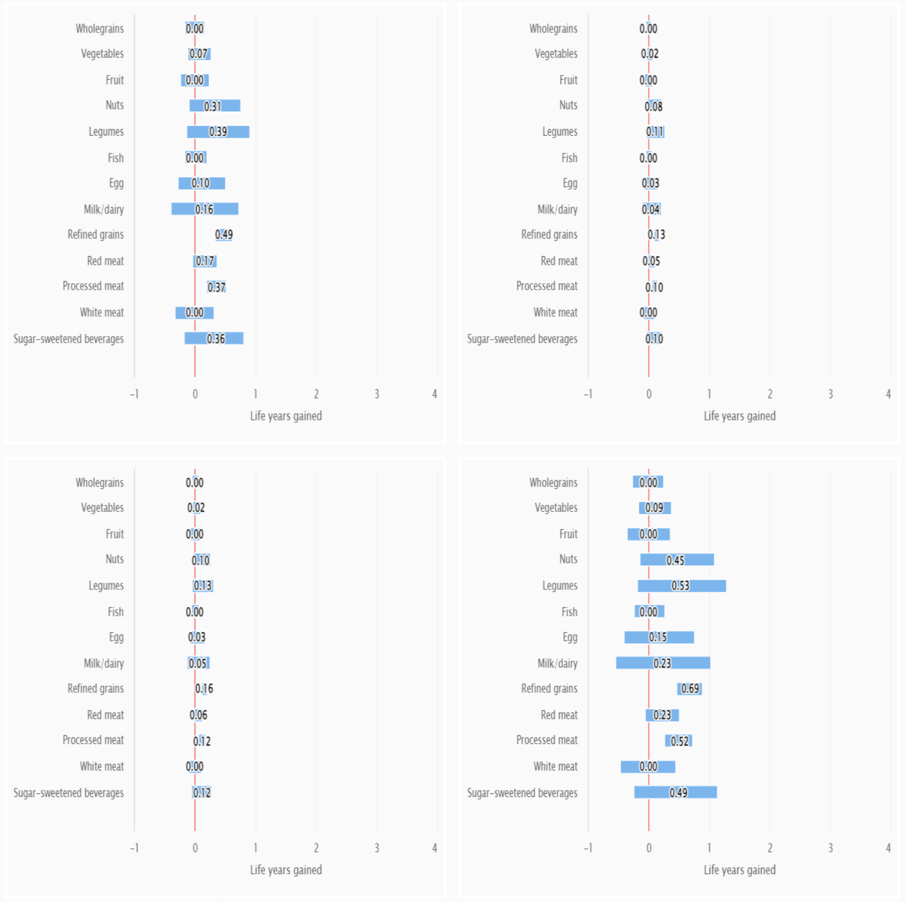

Fig S7. Expected life years gained per food group while adjusting for energy and body mass index presented in forest plots with uncertain intervals for 40-year-old female adults from the UK who changes from a: median to Eatwell Guide patterns (upper left), b: median to longevity-associated (lower left), c: unhealthy to Eatwell Guide patterns (upper right), d: unhealthy to longevity-associated (lower right).

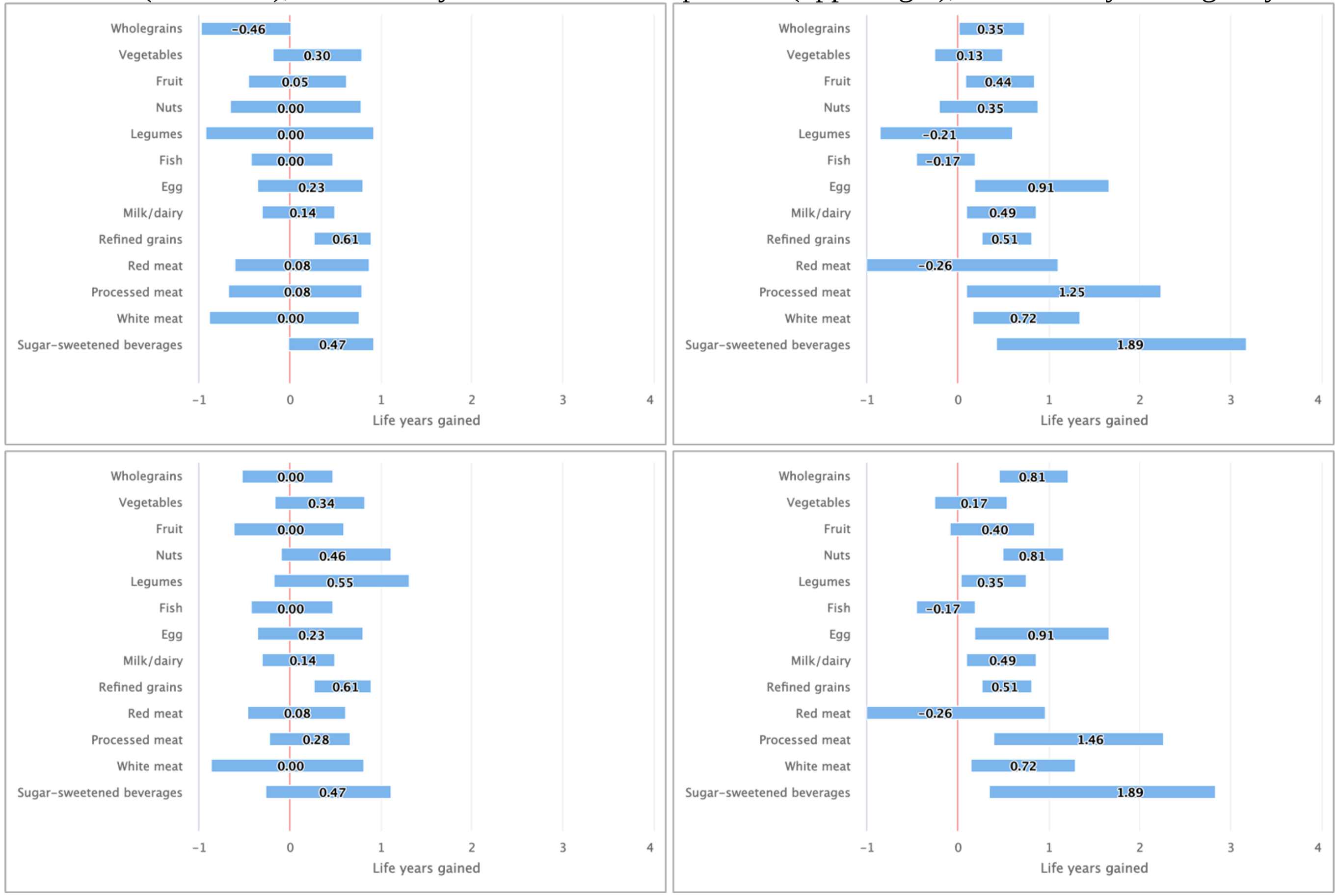

* Changes includes medium to high intake of whole grains, vegetables, fruits, nuts, legumes, fish, milk/dairy, and white meat, a low to medium intake of eggs, and a low intake of sugar-sweetened beverages, red and processed meats, and refined grains. For the last four groups, a reduction in the intake is associated with life expectancy gains.

Fig S8. Expected life years gained per food group while adjusting for energy and body mass index presented in forest plots with uncertain intervals for 70-year-old male adults from the UK who changes from a: median to Eatwell Guide patterns (upper left), b: median to longevity-associated (lower left), c: unhealthy to Eatwell Guide patterns (upper right), d: unhealthy to longevity-associated (lower right).

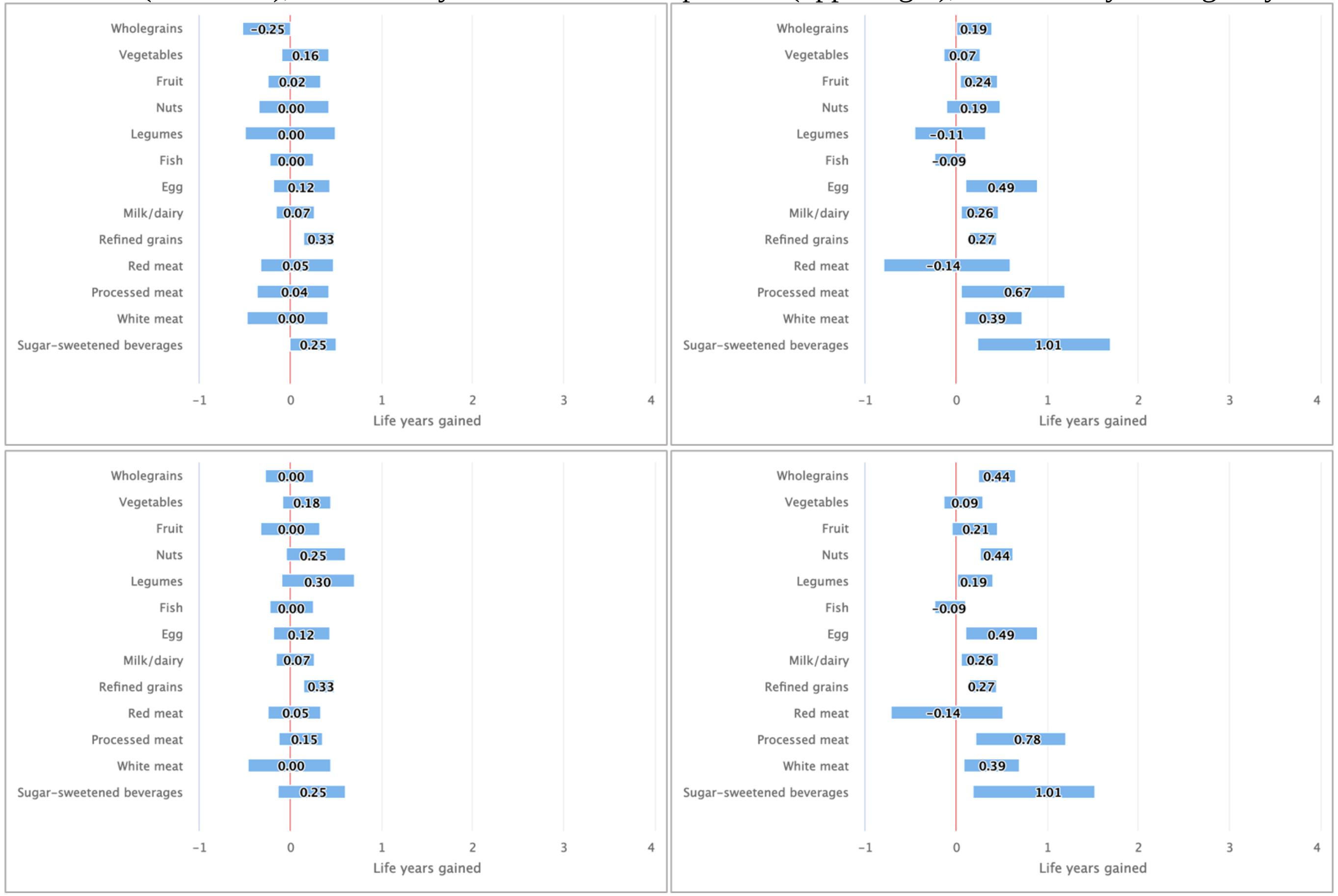

* Changes includes medium to high intake of whole grains, vegetables, fruits, nuts, legumes, fish, milk/dairy, and white meat, a low to medium intake of eggs, and a low intake of sugar-sweetened beverages, red and processed meats, and refined grains. For the last four groups, a reduction in the intake is associated with life expectancy gains.

Fig S9. Expected life years gained per food group while adjusting for energy and body mass index and energy presented in forest plots with uncertain intervals for 40-year-old female adults from the UK who changes from a: median to Eatwell Guide patterns (upper left), b: median to longevity-associated (lower left), c: unhealthy to Eatwell Guide patterns (upper right), d: unhealthy to longevity-associated (lower right).

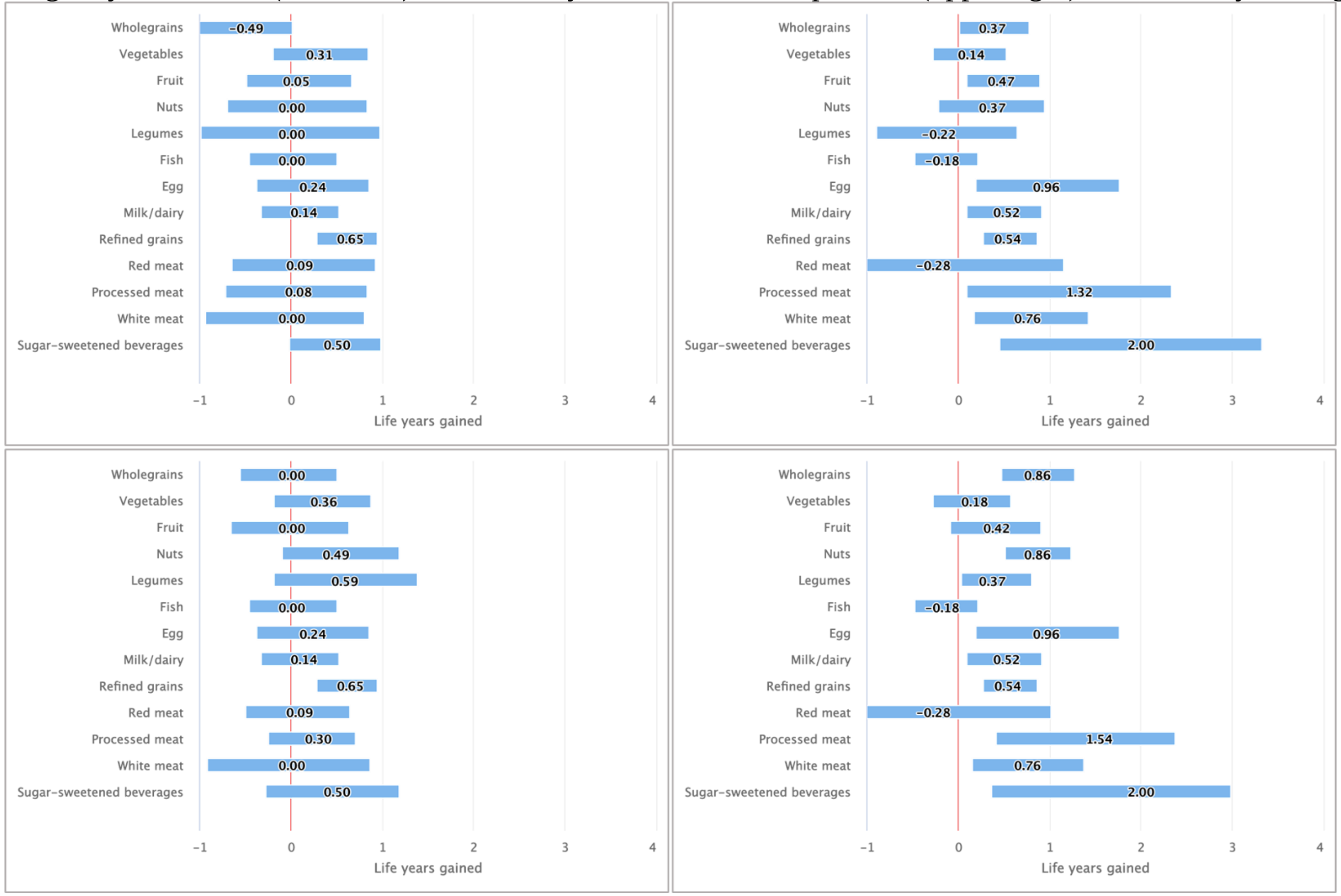

* Changes includes medium to high intake of whole grains, vegetables, fruits, nuts, legumes, fish, milk/dairy, and white meat, a low to medium intake of eggs, and a low intake of sugar-sweetened beverages, red and processed meats, and refined grains. For the last four groups, a reduction in the intake is associated with life expectancy gains.

Fig S10. Expected life years gained per food group while adjusting for energy and body mass index and energy presented in forest plots with uncertain intervals for 70-year-old male adults from the UK who changes from a: median to Eatwell Guide patterns (upper left), b: median to longevity-associated (lower left), c: unhealthy to Eatwell Guide patterns (upper right), d: unhealthy to longevity-associated (lower right).

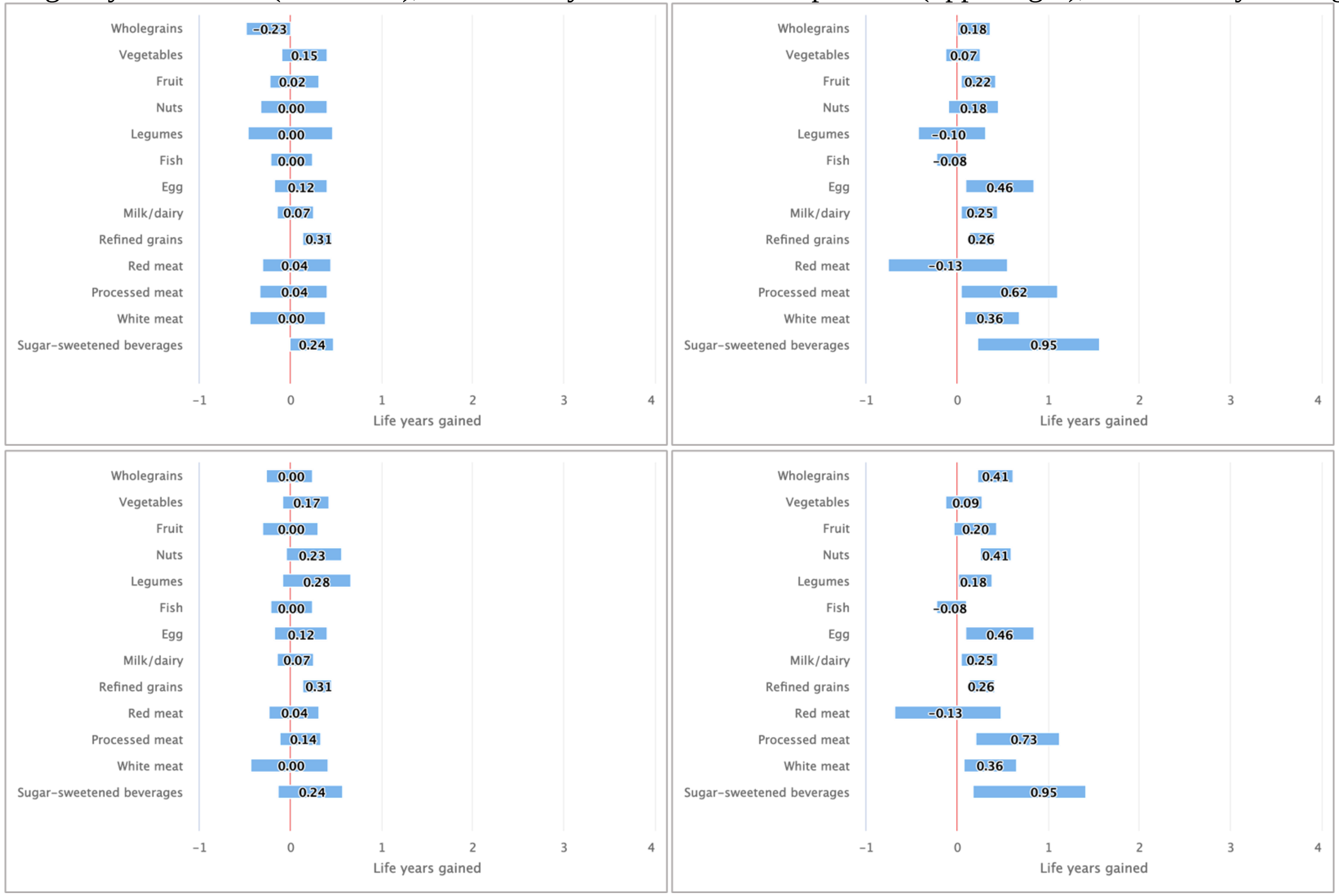

* Changes includes medium to high intake of whole grains, vegetables, fruits, nuts, legumes, fish, milk/dairy, and white meat, a low to medium intake of eggs, and a low intake of sugar-sweetened beverages, red and processed meats, and refined grains. For the last four groups, a reduction in the intake is associated with life expectancy gains.

Fig S11: Weight of mortality associated with “initial diet”.

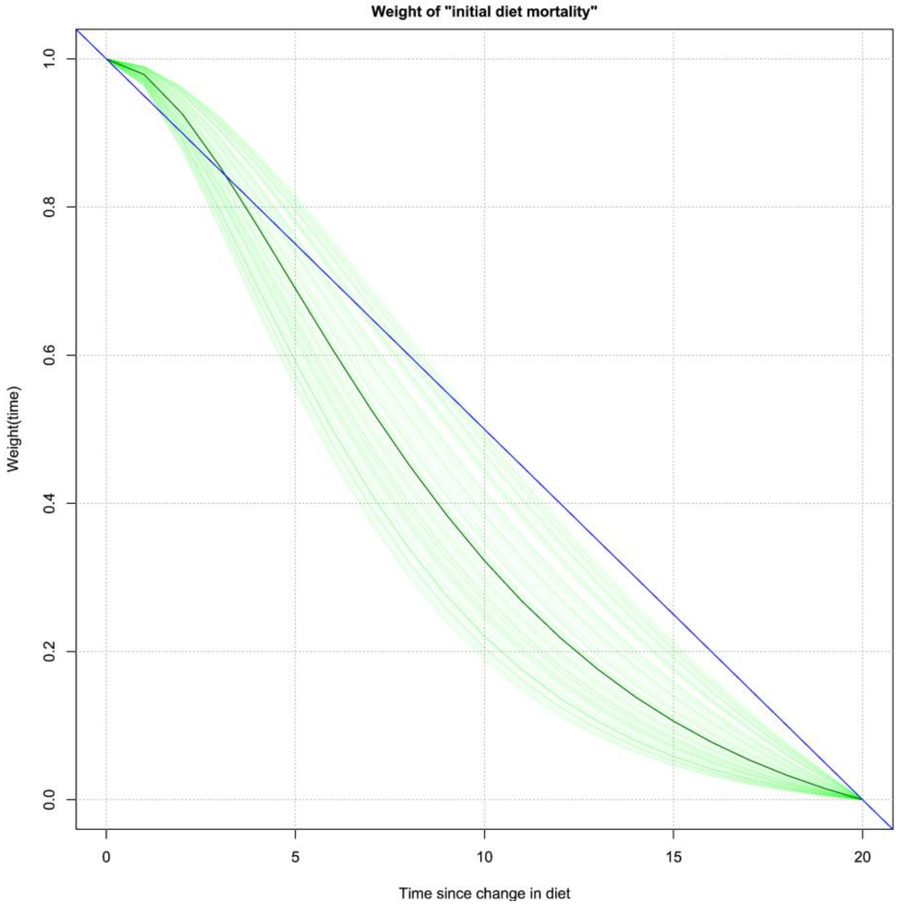

Mortality after t years is $\text{weight}(\text{t}) \times$ ”$\text{initial diet mortality}$” $+ \left(1 - \text{weight}(\text{t})\right) \times$ ”$\text{new diet mortality}$”. The dark green line is created by using the formula $\text{weight}(\text{time}) = \alpha + (\text{time} - \gamma) \times C \times e^{-\beta \times (\text{time} - \gamma)}$, and setting $\alpha = 0$, $\beta = 0.2125$, $C = 0.6244$, and $\gamma = -4.7059$. The light green lines are created by varying the coefficients in such a way that the function is anchored at 1 for time=0 and at 0 for time=20. The blue line corresponds to $\text{weight}(\text{time}) = \text{time}/20$. For more details about the weighting function, see Haaland *et al.* (2019).

Tripod Checklist S1.

TRIPOD Checklist: Prediction Model Development

Section/Topic	Item	Checklist Item	Page
**Title and abstract**			
Title	1	Identify the study as developing and/or validating a multivariable prediction model, the target population, and the outcome to be predicted.	1
Abstract	2	Provide a summary of objectives, study design, setting, participants, sample size, predictors, outcome, statistical analysis, results, and conclusions.	2
**Introduction**			
Background and objectives	3a	Explain the medical context (including whether diagnostic or prognostic) and rationale for developing or validating the multivariable prediction model, including references to existing models.	3
	3b	Specify the objectives, including whether the study describes the development or validation of the model or both.	3
**Methods**			
Source of data	4a	Describe the study design or source of data (e.g., randomized trial, cohort, or registry data), separately for the development and validation data sets, if applicable.	3+suppl.
	4b	Specify the key study dates, including start of accrual; end of accrual; and, if applicable, end of follow-up.	3+suppl.
Participants	5a	Specify key elements of the study setting (e.g., primary care, secondary care, general population) including number and location of centres.	suppl.
	5b	Describe eligibility criteria for participants.	suppl.
	5c	Give details of treatments received, if relevant.	
Outcome	6a	Clearly define the outcome that is predicted by the prediction model, including how and when assessed.	3+suppl.
	6b	Report any actions to blind assessment of the outcome to be predicted.	n.r.
Predictors	7a	Clearly define all predictors used in developing or validating the multivariable prediction model, including how and when they were measured.	3+suppl.
	7b	Report any actions to blind assessment of predictors for the outcome and other predictors.	n.r.
Sample size	8	Explain how the study size was arrived at.	3+suppl.
Missing data	9	Describe how missing data were handled (e.g., complete-case analysis, single imputation, multiple imputation) with details of any imputation method.	suppl.
Statistical analysis methods	10a	Describe how predictors were handled in the analyses.	suppl.
	10b	Specify type of model, all model-building procedures (including any predictor selection), and method for internal validation.	6+suppl.
	10d	Specify all measures used to assess model performance and, if relevant, to compare multiple models.	suppl.
Risk groups	11	Provide details on how risk groups were created, if done.	suppl.
**Results**			
Participants	13a	Describe the flow of participants through the study, including the number of participants with and without the outcome and, if applicable, a summary of the follow-up time. A diagram may be helpful.	3-4,suppl.
	13b	Describe the characteristics of the participants (basic demographics, clinical features, available predictors), including the number of participants with missing data for predictors and outcome.	4, suppl.
Model development	14a	Specify the number of participants and outcome events in each analysis.	suppl.
	14b	If done, report the unadjusted association between each candidate predictor and outcome.	4, suppl.
Model specification	15a	Present the full prediction model to allow predictions for individuals (i.e., all regression coefficients, and model intercept or baseline survival at a given time point).	4, suppl
	15b	Explain how to the use the prediction model.	4-5, suppl.
Model performance	16	Report performance measures (with CIs) for the prediction model.	4, suppl.
**Discussion**			
Limitations	18	Discuss any limitations of the study (such as nonrepresentative sample, few events per predictor, missing data).	5-6
Interpretation	19b	Give an overall interpretation of the results, considering objectives, limitations, and results from similar studies, and other relevant evidence.	5-6
Implications	20	Discuss the potential clinical use of the model and implications for future research.	5-6
**Other information**			
Supplementary information	21	Provide information about the availability of supplementary resources, such as study protocol, Web calculator, and data sets.	Suppl.
Funding	22	Give the source of funding and the role of the funders for the present study.	6-7

2 tables+figure+suppl.

We recommend using the TRIPOD Checklist in conjunction with the TRIPOD Explanation and Elaboration document.
